# Restoration of anti-bacterial innate immune cell function lags behind nutritional recovery among children convalescing from complicated severe acute malnutrition

**DOI:** 10.1038/s41467-026-75897-7

**Published:** 2026-07-28

**Authors:** Sandra Rukobo, Kuda Mutasa, Tracy N. Phiri, Margaret Govha, Patience Mushayanembwa, Simutanyi Mwakamui, Tafhima Haider, Ellen Besa, Kanekwa Zyambo, Cherlynn Dumbura, Joice Tome, Thompson Runodamoto, Florence D. Majo, Deophine Ngosa, Kanta Chandwe, Chanda Kapoma, Jonathan P. Sturgeon, Ruairi C. Robertson, Kusum Nathoo, Melanie Smuk, Robert Ntozini, Beatrice Amadi, Paul Kelly, Mutsa Bwakura-Dangarembizi, Andrew J. Prendergast, Claire D. Bourke

**Affiliations:** 1 Zvitambo Institute for Maternal and Child Health Research, Harare, Zimbabwe; 2 Tropical Gastroenterology and Nutrition group (TROPGAN), University of Zambia School of Medicine, Lusaka, Zambia; 3 School of Infection and Immunity, University of Glasgow, Glasgow, UK; 4 Blizard Institute, Queen Mary University of London, London, UK; 5 Department of Paediatrics and Child Health, University of Zimbabwe College of Health Sciences, Harare, Zimbabwe

**Keywords:** Translational research, Antimicrobial responses, Nutrition disorders, Monocytes and macrophages, Interleukins

## Abstract

Children recovering from complicated severe acute malnutrition (SAM) have a high risk of infectious mortality and morbidity, which could reflect impaired anti-microbial defence. We used blood samples from a cross-sectional cohort of children under 5 years’ old admitted to hospital with SAM in Zambia and Zimbabwe (cases, *n* = 125) and adequately nourished non-hospitalised children from the same communities (controls, *n* = 73) to characterise anti-bacterial innate immune cell function. We did not find evidence that inpatient immune function differed between cases who experienced subsequent adverse clinical outcomes (death, readmission, SAM at 48 weeks) versus those who did not. However, pro-inflammatory cytokine (IL-6, IL-8 and TNF) responses to *E. coli* lipopolysaccharide and heat-killed *Salmonella typhimurium* were positively associated with subsequent gains in mid-upper arm circumference, an indicator of nutritional recovery. We then characterised how innate immune cell function changed during post-discharge rehabilitation in a longitudinal sub-cohort of cases who provided repeat blood samples at discharge, 12, 24 and/or 48 weeks post-discharge (*n* = 83). Relative to inpatient immune function, bacterial binding capacity declined whilst anti-bacterial pro-inflammatory cytokine secretion increased in the cases over the post-discharge follow-up period with both normalising towards control ranges. These changes were also evident in cases who had recovered to a healthy nutritional status (weight-for-height *Z* score ≥ −2). Collectively, our data suggest that restoration of anti-bacterial innate immune cell function following complicated SAM lags behind nutritional recovery. Thus, children who are no longer wasted may continue to have deficient anti-bacterial innate immunity months after hospital admission for SAM.

## Abstract (web summary)

Children recovering after hospital admission with complicated severe acute malnutrition have a high risk of infectious mortality and morbidity. Here the authors analyse blood samples from a longitudinal cohort of children and show that restoration of innate immune cell function lags behind nutritional recovery over 48 weeks of post-discharge follow up.

## Introduction

Undernutrition is a major risk factor for mortality and morbidity among children who require hospitalisation in low-income settings. Affecting nearly twenty million children globally^1^, severe acute malnutrition (SAM, also termed severe malnutrition) is a form of undernutrition identified as severe wasting, nutritional oedema or both. Clinically, severe wasting is defined as low weight-for-height Z scores (WHZ) <−3 and/or mid-upper arm circumference (MUAC) < 115 mm. Children with SAM, particularly those requiring hospitalisation with clinical complications (complicated SAM), remain at high risk of mortality and morbidity after hospital discharge^2–7^. Complicated SAM is a heterogeneous condition with complications requiring hospitalisation defined as Integrated Management of Childhood Illness clinical danger signs, acute infection, severe oedema, or feeding problems^1^. When measured, mortality over the first year post-discharge can equal or exceed that reported during hospitalisation^8,9^. Mortality estimates from a systematic review of 7 studies among children with SAM who were considered “nutritionally cured” at discharge ranged from 0.1% to 10.4% over an average of 12 months of post-discharge follow-up^6^. Nutritional relapse rates are also high with estimates ranging from 0% to 37%^6,7^. A range of coincident risk factors, including persistent SAM at discharge despite resolution of clinical complications^2,3^, HIV infection^3^, disability^5^, and/or having been orphaned^7^ exacerbate poor outcomes during post-discharge rehabilitation. Mortality is predominantly due to severe disseminated infections and children with the most severe wasting (lowest WHZ or MUAC) are at higher risk of infectious mortality than children with moderate acute malnutrition (MAM, −3 < WHZ < −2) or a well-nourished (WN, WHZ ≥ −2) nutritional state^10,11^. Severe pneumonia^12^, persistent diarrhoea^12,13^, and bacteraemia^14^ are particularly common causes of infectious morbidity and mortality among children recovering from SAM. Despite significant improvements in clinical management^1,15^, and associated reductions in global prevalence of child undernutriton^16^, there remains an urgent need to understand the mechanisms that underlie persistent vulnerabilities among children recovering from SAM and how these might be targeted to improve their survival and health outcomes. Epidemiological evidence that infections are a predominant cause of death among these children^10,11^ and consistent observation that biomarkers of systemic inflammation and immune cell activation are highest in samples from those who die during hospitalisation and after discharge^17–20^, implicate a role for the immune system in SAM short- and long-term mortality.

We have previously shown in an observational cohort study of 745 children under 5 years’ old who were hospitalised with complicated SAM in Zambia and Zimbabwe (HOPE-SAM study), that mortality was 9.4% (95% CI: 7.4, 11.7%) during hospitalisation and 9.1% (95% CI: 6.9, 11.7%) over 52 weeks of post-discharge follow-up^13^. Hazards of death were highest during the first 12 weeks post-discharge but persisted throughout the year of follow-up after index hospital admission^3^. In a sub-study focused on immune cell function (IMMUNO-SAM), we assessed anti-bacterial responses of circulating immune cells in blood samples from a sub-set of these children (*n* = 141) during their hospital stay, identifying a distinct response compared to adequately-nourished non-hospitalised controls from the same communities (*n* = 92)^21^. Monocytes and neutrophils from children with SAM had higher capacity to bind to bacterial antigens in vitro and cross-sectional analyses showed that this difference reduced with time post-admission. Furthermore, children with the highest inpatient scores for bacterial binding capacity had higher odds of persistent SAM at discharge^21^, a risk factor for subsequent mortality and slower gains in MUAC and WHZ over a year of follow-up^2,3^. Collectively, these data suggested that shifts in anti-bacterial innate immune cell function may contribute to the pathophysiology of complicated SAM, do not appear to be resolved during inpatient rehabilitation, and may perpetuate health deficits among children who survive to hospital discharge relative to adequately nourished non-hospitalised controls. Few studies have evaluated immune cells or their function longitudinally in children with SAM, and those that have, have tended to focus on the inpatient period^22,23^. However, studies of soluble immune proteins suggest that systemic inflammation and nutritional rehabilitation are interrelated^17^. In our study among children in Zambia and Zimbabwe, there was a positive association between WHZ gains and a combination of high plasma levels of vascular growth/repair and myelopoiesis-promoting factors (epidermal growth factor (EGF), vascular endothelial growth factor (VEGF), D-dimer, granulocyte-colony stimulating factor (GCSF), and angiopoietin) and low plasma levels of acute phase response (C-reactive protein; CRP) and vascular inflammation (Vascular Cell Adhesion Molecule (VCAM)−1) mediators^24^. Similarly, among children hospitalised with complicated non-oedematous SAM in Bangladesh who transitioned to MAM, WHZ was positively associated with 215 plasma proteins associated with neurodevelopment, ossification, nutrient metabolism and extracellular matrix organisation but negatively associated with biomarkers of immune responses to infection and cellular stress, including antimicrobial peptides (granulysin) and complement proteins (C2, C4, C6 and C9)^25^. Plasma levels of pro-inflammatory mediators during inpatient treatment for SAM normalised to those of non-hospitalised children with MAM (*n* = 21) after 4 weeks of a novel legume-based feeding intervention^26^. The pro-inflammatory mediators, vascular activation and myelopoietic processes that these studies implicate in compromising recovery from SAM, could plausibly influence longitudinal systemic immune cell composition and responses to bacterial infection, as suggested by recent murine models of dietary macronutrient restriction^27–30^.

In this study we explored whether rehabilitation of anti-bacterial innate immune cell function (immune restoration) is associated with anthropometric recovery (nutritional recovery) using longitudinal blood samples collected from our existing IMMUNO-SAM cohort^21^. We tested 3 key hypotheses: 1) immune function during hospitalisation is associated with subsequent adverse clinical outcomes and/or ponderal growth gains post-discharge (ΔMUAC and ΔWHZ); 2) anti-bacterial innate immune cell function changes at discharge, 12, 24 and 48 weeks relative to their baseline inpatient immune function and compared to heathy controls; and, 3) anti-bacterial immune function of children who have made a full nutritional recovery from SAM continues to differ from that of healthy controls.

## Results

### Demographic and clinical characteristics of the study cohort

Children under 5 years old admitted to 3 peri-urban tertiary hospital sites in Zambia and Zimbabwe with complicated SAM (defined according to WHO criteria^1^) and a known HIV status were enroled in a longitudinal observational cohort study (Health Outcomes, Pathogenesis and Epidemiology of SAM; HOPE-SAM^31^). Children were evaluated at hospital admission and followed up at 5 pre-defined post-discharge visits (2, 4, 12, 24 and 48 weeks) with assessment of anthropometry and clinical outcomes at all visits. Full details of clinical outcomes from the full HOPE-SAM cohort (*n* = 745 cases) have been previously reported^2,3^. Briefly, mortality among cases was 9.4% during hospitalisation and 9.1% over the post-discharge follow-up period^2,3^; of the 649 children discharged from hospital, vital status at the end of follow-up was ascertained for 93.1%. Post-discharge morbidity was high with 48.8% of cases having ≥1 caregiver reported symptom by week 4 post-discharge and 15.4% of cases re-admitted to hospital^2^. SAM was still present in 43.9% of cases at discharge, 12.4% at week 12, 11.9% at week 24, and 4.5% by the end of follow-up^2^.

**Fig. 1:**
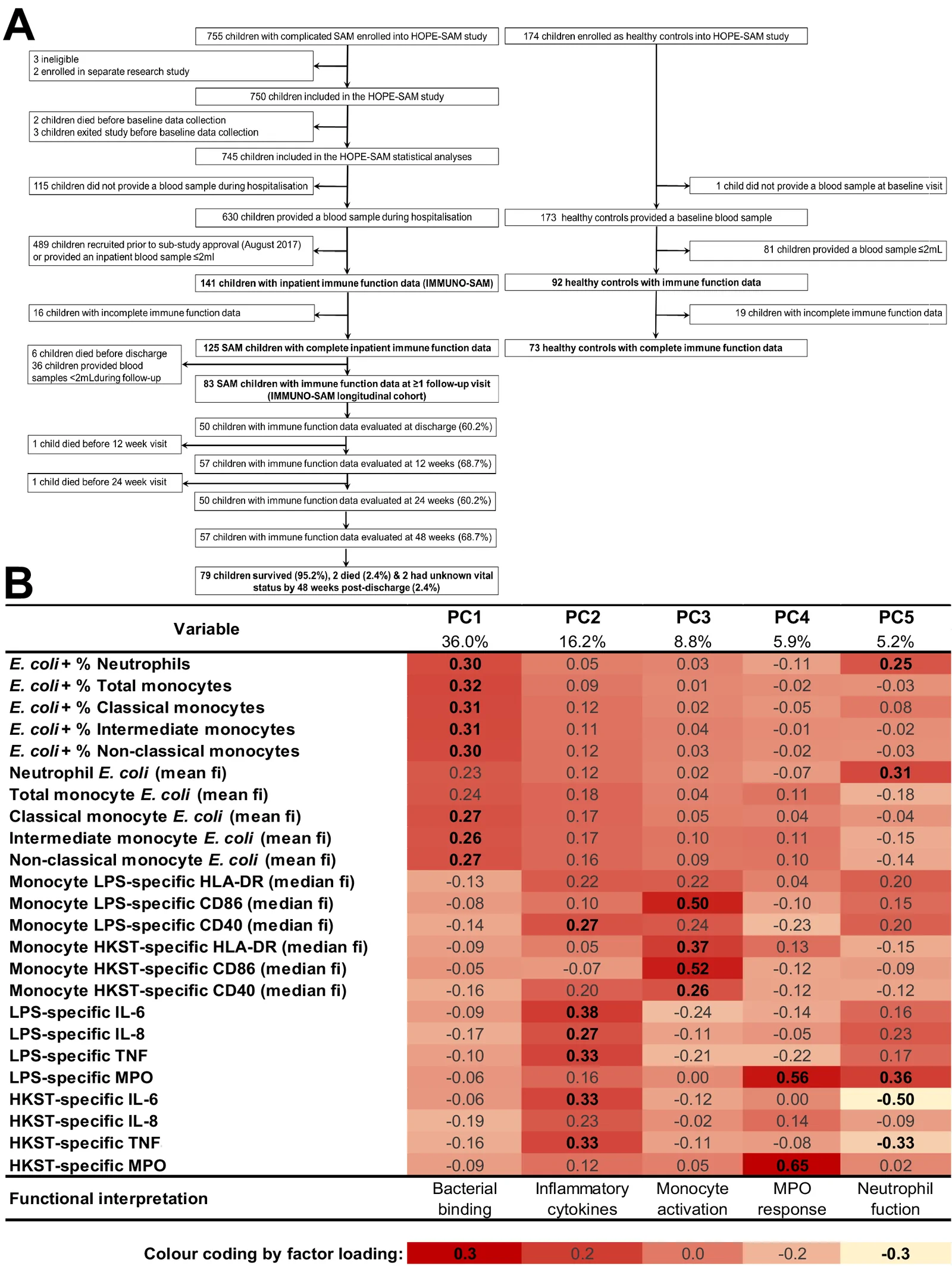
IMMUNO-SAM cohort of children hospitalised with complicated SAM (cases) and healthy controls evaluated for immune cell function cross-sectionally and longitudinally. **A** Diagram indicating cohort selection relative to the parent clinical study (HOPE-SAM; N = 750 cases, 174 controls)^3,24^ and previously-reported IMMUNO-SAM cross-sectional cohort among whom inpatient immune cell function was assessed in cases (*n *= 141) and controls (*n* = 92)^21^. All IMMUNO-SAM cases and controls with complete immune function assessment were included in this study as the cross-sectional cohort (cases *n* = 125, controls *n* = 73) and all cases in the cross-sectional cohort who had complete immune function data at ≥1 follow-up visit were included in the longitudinal cohort (*n* = 83); number and percentage of cases from the longitudinal cohort with available immune function data are indicated for each follow-up visit. **B** Factor loadings for individual antibacterial innate immune function variables included in a PCA of all children with complete data on all variables (125 cases, 73 controls); as previously reported, only data from the first immune function assessment for each child were included^21^. *E. coli* percentages reflect the cells positive for the *E. coli*-coated AF488-labelled bioparticles, and mean FI the amount of AF488 fluorescence detected for each cell type in the bacterial binding assay. LPS- and HKST-specific monocyte marker expression (median FI) and supernatant mediator concentration (pg/mL) are the values for each stimulus with the background levels in parallel cultures without stimulus subtracted (Δ). Table is colour-coded by strength and direction of association between the original variables and the resulting principal components (PC), with darker red indicating a stronger positive association; coefficients ≥ 0.3 (strong positive) or ≤ −3 (strong negative) are bolded. Column percentages correspond to the amount of variance in the dataset accounted for by each PC. Functional interpretation reflects the combination of the original variables captured by each PC.

The immunology sub-study (IMMUNO-SAM) included HOPE-SAM participants who provided a blood sample ≥2 mL for immune function assessment (bacterial binding assay and whole blood culture) during hospitalisation and had complete data on all immune function read-outs (cases *n* = 125; Fig. 1A). A group of adequately nourished non-hospitalised children who were not acutely unwell and had a known HIV status were recruited from the same sites for all HOPE-SAM sub-studies and evaluated at a single timepoint (baseline); those who provided ≥2 mL blood samples were included as healthy controls for the IMMUNO-SAM study (controls, *n* = 73; Fig. 1A). Demographic characteristics of the IMMUNO-SAM cases and controls at enrolment are summarised in Table 1. Given the requirement for an inpatient blood sample, cases included in the IMMUNO-SAM cohort tended to be healthier at hospital admission (i.e., lower mortality during hospitalisation, slightly older, with less severe wasting, and a lower percentage with SAM at discharge) than the HOPE-SAM cohort as a whole; a full comparison of clinical and demographic characteristics during the inpatient period has been previously reported^21^. Within the IMMUNO-SAM sub-study, fourteen cases (11.9% of 118 cases with mortality data) died, 22 (20.2% of 109 cases with re-admission data) experienced ≥1 hospital readmission (mean: 1.4 readmissions per child, Standard Deviation (SD): 0.7), and 58 (53.2% of 109 cases with clinic visit data) had ≥1 clinic visit outside their normal schedule (mean: 1.5, SD: 0.7), and 68 (62.4% of 109 cases with symptom data) experienced ≥1 caregiver-reported symptom consistent with an infection during the 48 week follow-up period. Post-discharge clinical outcomes for the IMMUNO-SAM cases are summarised in Table 2. Repeat blood samples were collected at hospital discharge, and at the 12, 24 and 48 week post-discharge study visits for longitudinal evaluation of immune cell function. Of the 125 cases with complete inpatient immune function assessment, 83 (66.4%) had a repeat assessment of immune function at ≥1 follow-up visit (longitudinal cohort; Fig. 1A).

**Table 1:** Summary of baseline demographic and clinical characteristics of SAM cases in the IMMUNO-SAM cross-sectional cohort during their index hospital admission and healthy controls at baseline

	Healthy Controls	SAM Cases
**N**^**1**^	**73**	**125**
Hospital site^2^		
- Harare Central Hospital, n (%)	30 (41.1%)	53 (42.4%)
- Parirenyatwa Hospital, n (%)	38 (52.1%)	53 (42.4%)
- University Teaching Hospital, n (%)	5 (6.9%)	19 (15.2%)
**Participant characteristics at baseline**^**3**^**:**		
Age (months), mean (SD)	30.3 (15.7)	18.6 (8.6)
Age group (months)^4^:		
0–11	10 (13.7%)	23 (18.4%)
12–23	23 (31.5%)	80 (64.0%)
24–59	40 (54.8%)	22 (17.6%)
Male, *n* (%)	38 (52.1%)	66 (52.8%)
Cerebral palsy, *n* (%)	0	5 (4.0%)
Birthweight (kg) N = 67, 115, mean (SD)	3.0 (0.5)	2.9 (0.5)
**Child HIV status:**		
HIV positive, N = 70, 125, *n* (%)	27 (38.6%)	25 (20.0%)
- Taking ART N = 27, 23, *n* (%)	27 (100.0%)	16 (69.6%)
HIV-exposed uninfected N = 70, 125, *n* (%)	22 (31.4%)	19 (15.2%)
HIV-unexposed uninfected, N = 70, 125, *n* (%)	24 (34.3%)	81 (64.8%)
**Nutritional status:**		
Baseline MUAC (mm), mean (SD)	151 (11.2)	120 (19.4)
Baseline WHZ score, mean (SD)	0.4 (0.9)	-2.6 (2.0)
Baseline oedema, *n* (%)	-	87 (69.6%)
Baseline HAZ score, mean (SD)	-1.8 (1.4)	-2.9 (1.6)
- Baseline stunting status (HAZ < -2), *n* (%)	32 (43.8%)	58 (46.4%)

^1^Total number of children included in the IMMUNO-SAM cohort with complete immune function data. Where data on a specific variable are missing, n refers to the number of participants positive for that variable and N to the total number of participants with available data for that variable in controls, cases.

^2^Immune function assessments were undertaken in centralised laboratories in Zimbabwe (Harare Central and Parirenyatwa) and Zambia (University Teaching Hospital).

^3^Individual clinical complications present alongside SAM at hospital admission in this cohort are reported elsewhere^21^.

^4^Healthy controls were recruited from age 6-60 months and SAM cases from 0-60 months per HOPE-SAM protocol^31^; age groupings for enrolment were pre-specified for HOPE-SAM recruitment and analysis of mortality outcomes^3,31^; the <6mo (cases *n* = 7; controls *n* = 0) and 6–11 (cases *n* = 16; controls *n* = 10) month age groups were combined for IMMUNO-SAM analyses.

**Table 2:** Summary of clinical outcomes for SAM cases in the IMMUNO-SAM cross-sectional cohort during 48 weeks of post-discharge follow-up

	SAM Cases
**N**^**1**^	**125**
Time to hospital discharge (days), mean (SD)	13.5 (9.1)
**Nutritional status:**	
SAM at follow up visits, n (%):	
- Discharge, N = 118	51 (43.2%)
- 2 weeks, N = 90	29 (32.2%)
- 4 weeks, N = 91	21 (23.1%)
- 12 weeks, N = 90	10 (11.1%)
- 24 weeks, N = 93	13 (14.0%)
- 48 weeks, N = 85	7 (8.2%)
**Clinical outcomes over 48 weeks of post-discharge follow-up:**	
Mortality^2^, N = 118, n (%)	14 (11.9%)
Children re-admitted to hospital^3^ N = 109, n (%)	22 (20.2%)
- Number of re-admissions per child, N = 22, mean (SD)	1.4 (0.7)
Children who attended clinic for reasons outside normal schedule^3^ N = 109 n (%)	58 (53.2%)
- Number of clinic visits per child, N = 58, mean (SD)	1.5 (0.7)
Children with a caregiver reported symptom of infection^4^ N = 109, n (%)	68 (62.4%)

^1^Total number of children included in the IMMUNO-SAM cohort with complete immune function data. Where data on a specific variable is missing, n refers to the number of participants positive for that variable and N to the total number of participants with available data for that variable.

^2^Child vital status was determined from caregiver interview or hospital records. Follow-up phone calls were made to reschedule missed appointments, and home visits were undertaken for chronic defaulters where possible. Vital status was ascertained by telephone calls for those who had defaulted follow-up.

^3^Hospital re-admission and unscheduled clinic visits were reported by caregivers at each study visit. Details were verified from caregivers’ hand-held child health cards, where available. Hospital admissions solely for pre-existing congenital or developmental conditions (e.g., congenital heart disease) or due to traumatic injury not plausibly associated with immunological differences (e.g., burns, broken bones) are not included. Full details of all-cause re-admissions previously reported^2^.

^4^Caregiver-reported symptoms in the previous 2 weeks were recorded at each study visit^.^ Illnesses identified at follow-up visits were managed by study doctors, with referral to hospital, if needed. Symptoms associated with pre-existing congenital or developmental conditions (e.g., congenital heart disease) or due to traumatic injury not plausibly associated with immunological differences (e.g., burns, broken bones) are not included.

### Inpatient immune function did not differ between children who went on to have adverse clinical outcomes post-discharge

**Fig. 2:**
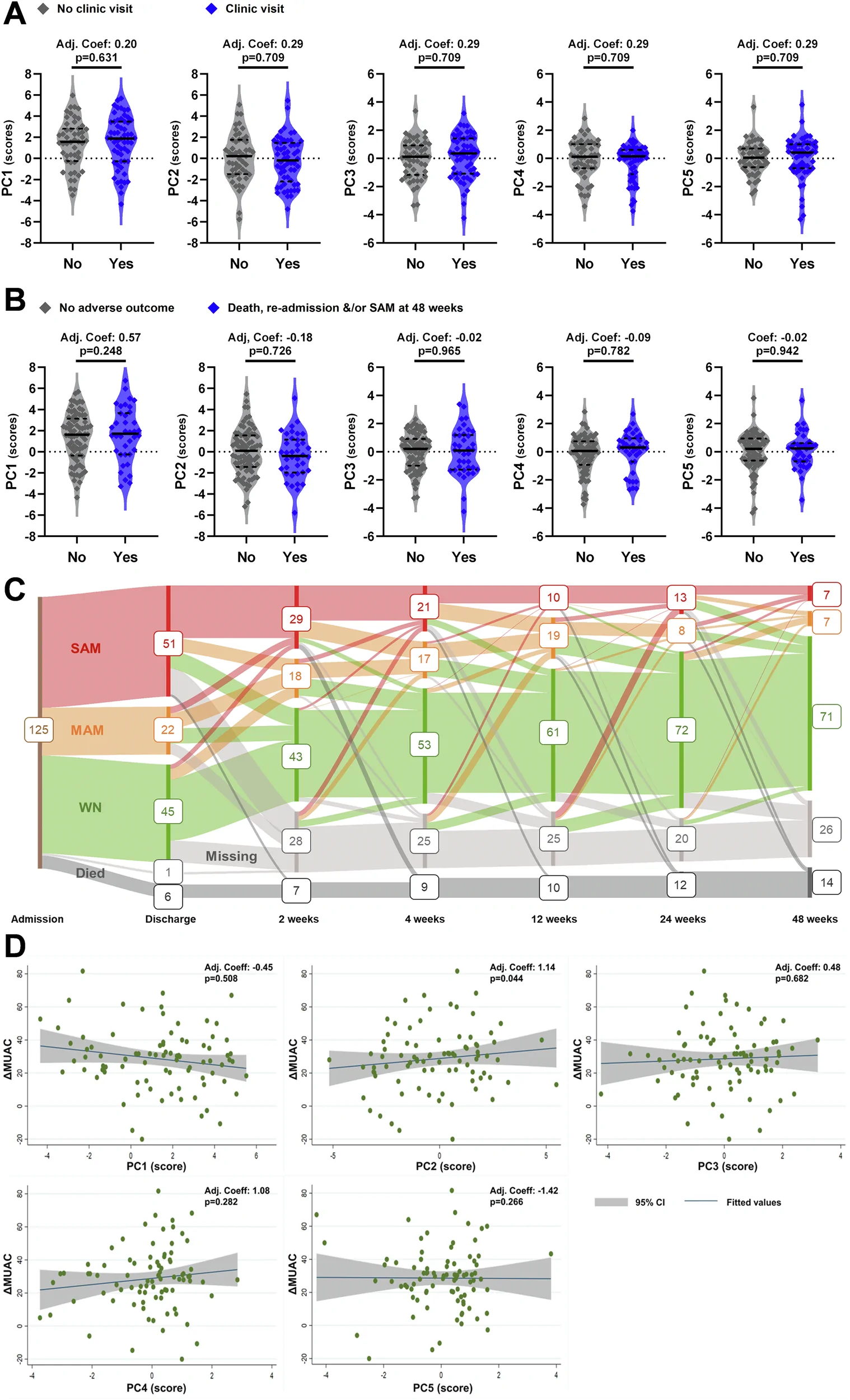
Anti-bacterial innate immune cell function during hospitalisation is positively associated with gains in MUAC but does not differ between children who attended additional clinic visits or experienced adverse clinical events over 48 weeks of post-discharge follow-up. **A** Violin plots of inpatient PC1-5 scores between cases whose caregiver reported ≥1 clinic visit outside of routine postnatal care (blue; *n* = 58/109 (53.2%)) versus did not attend clinic (grey; *n* = 51/109 (46.8%)) at any post-discharge follow-up visit. **B** Comparison of inpatient PC1-5 scores between cases who experienced (blue; *n* = 38/116 (32.8%)) versus did not experience (grey; *n *= 78/116 (67.2%)) death, ≥1 readmission to hospital, and/or had SAM at the 48-week post-discharge visit during follow-up. **C** Sankey diagram showing the number of cases in the IMMUNO-SAM cross-sectional cohort who were classified as SAM (red), MAM (orange), of well-nourished (green) or who had missing data (light grey) at each follow-up visit; cumulative numbers of children who had died by each visit (dark grey) are indicated at the bottom. **D** Scatterplots of change (Δ) in MUAC between admission and 48 week visit versus PC1-5 scores of the cases at initial inpatient immune function assessment (*n *= 85); blue lines indicate fitted values and grey shading indicates 95% CI. **A**, **B** Results of multivariable linear regression of PC scores by clinical outcome, adjusted for sex, age group at enrolment, HIV status, cerebral palsy, country of recruitment, baseline oedema, HAZ and MUAC, and SAM status at discharge are indicated by adjusted coefficient (Adj. Coeff) and two-sided *p* value. **D** Results of multivariable linear regression of PC scores by ΔMUAC, adjusted for sex, age group at enrolment, HIV status, cerebral palsy, country of recruitment, baseline oedema and MUAC, and SAM status at discharge are indicated by Adj. Coeff, *p* value, fitted values and 95% CI.

An effective cellular immune response to bacterial challenge reflects a balance of different innate immune cell functions, as has been reported for monocyte/macrophage responses in human sepsis^32–34^. We have previously reported Principal Component Analysis (PCA) of all individual anti-bacterial innate immune function read-outs from whole blood culture and bacterial binding assays for IMMUNO-SAM cases during their hospitalisation and controls at baseline, identifying 5 key domains of combined innate anti-bacterial immune function (Principal Components (PC); factor loadings for all individual variables included in the PCA are shown in Fig. 1B)^21^. These composite variables have the advantage of reducing the number of variables for statistical analyses and better reflect the interacting nature of anti-bacterial immune responses in vivo than evaluating individual immune functions one at a time. PC scores correspond to patterns of the immune function variables with which they are loaded (e.g., PC2 is positively loaded with tumour necrosis factor (TNF), interleukin (IL)−6 and IL-8 in response to bacterial antigens (LPS and HKST), therefore higher scores for PC2 reflect higher pro-inflammatory cytokine responses to bacterial antigens). Our first hypothesis for the longitudinal clinical dataset was whether cases who experienced adverse clinical outcomes post-discharge would have distinct patterns of innate anti-bacterial immune function during index hospitalisation compared to those who did not experience adverse outcomes. We compared inpatient scores for PC1-5 between cases with versus without caregiver-reported clinic visits outside of routine postnatal care at any of the scheduled post-discharge follow-up visits (Fig. 2A; Supplementary Table 1; 109 cases had available clinic visit data for at least one follow-up visit (87.2%)) and between children who experienced adverse clinical outcomes (i.e., death, readmission to hospital and/or persistent SAM at 48 weeks post-discharge) versus those who did not (Fig. 2B; and Supplementary Table 2; 116 cases had available adverse outcome data (92.8%)). We did not find evidence to support a difference in inpatient immune cell function between children with or without clinic visits nor between children with or without adverse clinical outcomes after hospital discharge in univariable regression analysis or when regression models were adjusted for baseline characteristics previously identified to be associated with adverse clinical outcomes in the HOPE-SAM cohort (sex, age group, oedema, HIV status, MUAC, cerebral palsy, height-for-age Z score), country of recruitment or persistent SAM at discharge^2,3,21^. This was also true of the relationship between PC scores and more severe adverse outcomes when evaluated separately (Supplementary Fig. 1; and Supplementary Table 3).

### Post-discharge MUAC and WHZ are positively associated with inpatient pro-inflammatory cytokine responses to bacterial antigens

After baseline assessment, cases had a heterogeneous course of nutritional recovery, but the majority had nutritionally recovered from SAM by the 48 week visit (78/85 cases with nutritional status data at 48 week visit (91.8%); Fig. 2C). Of these, 7 cases (8.2%) had progressed from SAM to MAM and 71 cases (83.5%) to WN (Fig. 2C). In the HOPE-SAM cohort, baseline wasting severity, indicated by low MUAC and/or WHZ, was a predictor of post-discharge nutritional rehabilitation^2^ and was positively associated with the capacity of blood immune cells to produce pro-inflammatory cytokines, collectively characterised in PC2^21^. We therefore evaluated whether patterns of anti-bacterial innate immune cell function were associated with the change in MUAC (Fig. 2D; Supplementary Table 4) or WHZ (Supplementary Fig. 2; and Supplementary Table 5) in cases from baseline to the 48 week post-discharge visit. We adjusted analyses for variables previously shown to be associated with ponderal growth gains after discharge (country, sex, age group at enrolment, HIV status, cerebral palsy, SAM at discharge, baseline HAZ and WHZ or MUAC). We identified a positive association between baseline PC2 and increase in MUAC (Adjusted Coefficient (Adj. Coef.): 1.14, 95% Confidence Interval (95%CI): 0.03, 2.25, p = 0.044). We saw a similar pattern, albeit not statistically significant, between baseline PC2 and increase in WHZ (Adj. Coef: 0.10, 95%CI: -0.01, 0.21, p = 0.065). This reflected positive associations between increase in MUAC (Supplementary Fig. 3; Supplementary Table 6) and increase in WHZ (Supplementary Fig. 4; and Supplementary Table 7) and the individual inpatient LPS- and HKST-induced cytokines loaded onto PC2.

Monocytes are a major source of pro-inflammatory cytokines in response to bacterial antigens in the blood and differentiate along a continuum from the classical (CD14 + CD16-) to intermediate (CD14 + CD16 + ) to non-classical (CD14^lo^CD16 + ) phenotypes with distinct capacities to respond to pathogen antigens^35^. We therefore undertook supplementary unadjusted linear regression of the relationship between proportions of total monocytes (Lineage- (i.e., CD3-CD19-CD20-CD56-) CD66b- HLA-DR + ) within all blood leucocytes and monocyte subsets within the total monocyte population and gains in MUAC from baseline to 48 weeks among the cases. We did not find evidence to support a significant association between log-transformed proportions of total monocytes (Coef: 53.72, 95%CI: -191.09, 298.52, *p* = 0.664) or monocyte subsets (classical Coef: 25.32, 95%CI: -10.63, 61.28, *p* = 0.165; intermediate Coef: -31.15, 95%CI: -99.20, 36.90, *p* = 0.365; non-classical Coef: -17.04, 95%CI: -57.21, 23.13, *p* = 0.401) and gains in MUAC. Collectively, our data indicate that cases who have a heightened blood immune cell capacity to secrete pro-inflammatory cytokines in response to bacterial antigens during hospitalisation may go on to have a fuller nutritional recovery post-discharge. This pattern was not solely due to differences in monocyte composition.

### Proportions of monocytes and monocyte HLA-DR expression are persistently lower in children recovering from SAM

The longitudinal cohort of cases (*n* = 83) allowed us to assess how circulating immune cell composition and anti-bacterial function of cases changed over 48 weeks of post-discharge follow-up. For our longitudinal mixed effects linear regression of immune function variables by visit and case/control status, we refer to immune restoration where cases had distinct scores for an immune function variable compared to controls at baseline, but these scores subsequently converged to be within control ranges during post-discharge follow-up. Normalisation was indicated by scores becoming more like those of the control group, even they it remained significantly different at post-discharge visits.

Proportions of unstimulated blood lymphocytes (mixed population of CD3 + T cells, CD19+ and CD20 + B cells and CD56 + NK cells) and neutrophils (CD66b + CD16 + ) were similar between cases and controls at baseline and did not significantly differ in cases relative to their baseline proportions at post-discharge follow-up visits (Supplementary Fig. 5; Supplementary Table 8). In contrast, proportions of unstimulated total blood monocytes were significantly lower in the cases relative to the controls at baseline (Coef: -0.01, 95%CI: -0.02, 0.00, *p* = 0.030) and did not change relative to baseline in the cases post-discharge (Supplementary Fig. 5; Supplementary Table 8). Within the total monocyte population, cases had similar proportions of classical, higher proportions of intermediate and lower proportions of non-classical monocytes compared to controls at baseline. However, in cases, proportions of intermediate monocytes in blood samples were significantly lower at discharge, 12, 24 and 48 week follow-up visits relative to baseline and non-classical monocytes were significantly higher from week 12 onwards relative to baseline proportions (Supplementary Fig. 5; Supplementary Table 8).

We further investigated longitudinal changes in monocyte surface marker expression (Supplementary Fig. 6; Supplementary Table 9) and spontaneous cytokine secretion in unstimulated whole blood cultures in the cases (Supplementary Fig. 7; and Supplementary Table 9). At baseline, monocytes from cases had lower surface expression of the major histocompatibility class II molecule HLA-DR, and higher expression of the costimulatory molecule CD86 than controls. Within cases, HLA-DR expression increased relative to baseline at 12, 24 and 48 weeks post-discharge (Supplementary Fig. 6A). Monocyte CD86 expression decreased relative to baseline at discharge, 12, and 24 weeks but was similar to baseline at 48 weeks post-discharge (Supplementary Fig. 6B). Concentrations of pro-inflammatory cytokines (IL-6, IL-8 and TNF) in unstimulated cultures were similar and myeloperoxidase (MPO) was higher in cases versus controls at baseline (Supplementary Fig. 7). By 48 weeks, levels of pro-inflammatory cytokines were higher than those at baseline in cases. MPO production increased at 12 and 24 weeks relative to baseline levels in cases but was similar to baseline by 48 weeks. Thus, dysregulated circulating monocyte phenotypes and pro-inflammatory cytokine secretion in cases at hospital admission appear to normalise with time post-discharge.

### Bacterial binding capacity normalises over the course of nutritional rehabilitation from SAM

**Fig. 3:**
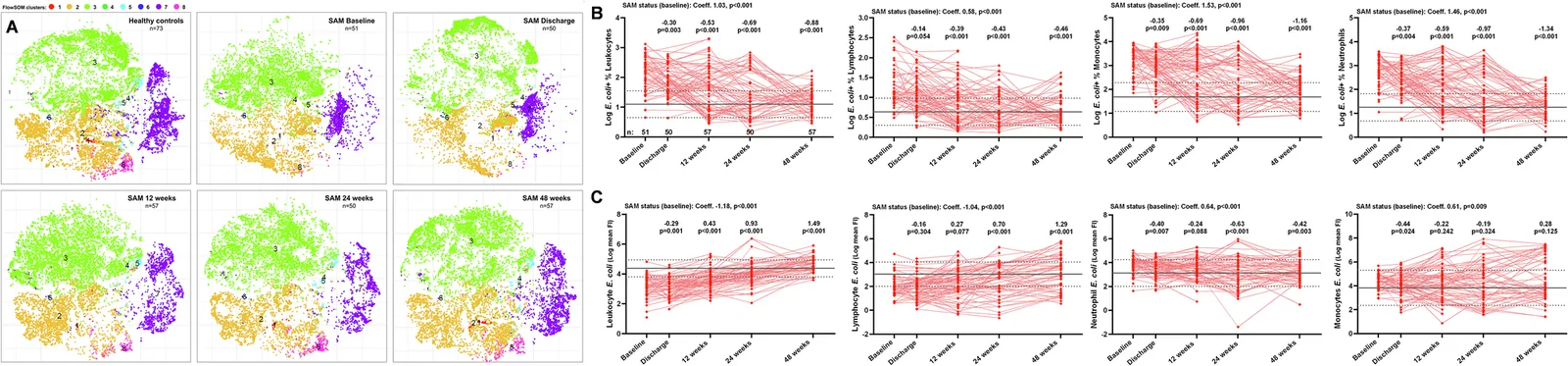
Bacterial binding capacity of blood immune cells from children recovering from complicated SAM declines but remains distinct from that of healthy controls months after hospital discharge. Bacterial binding capacity was assessed in whole blood samples from 83 cases (50 at baseline, 32 at discharge) and repeated samples were collected at discharge, 24 and 48 weeks post-discharge and 73 controls. **A** tSNE plot of 60,000 *E. coli*+ single leucocytes derived from controls and cases at each follow-up visit (10,000 events per visit sampled from 200 events per blood sample) colour-coded by immunophenotype (FlowSOM clusters summarised in Figure S8). **B** Spaghetti plots of longitudinal *E. coli*+ cells as a percentage of total leucocytes (left), Lineage (CD3, CD19, CD20, CD56)+ mixed lymphocytes (centre left), Lineage-CD66b-HLA-DR+ total monocytes (centre right), and CD66b + CD16+ neutrophils (right) relative to percentages in healthy controls (*n* = 73; mean and standard deviation indicated in grey for a single sampling time point) and, **C** Longitudinal mean AF488 mean FI for each cell type of cases relative to controls. Red lines link longitudinal data from the same child. **B**, **C** Results of unadjusted mixed regression models of log(*n *+ 1) transformed percentages or log(*n* + 1) transformed mean FI by baseline SAM status, visit and the interaction between SAM status and visit as fixed effects and participant identifier and SAM status as random effects are indicated. Coefficients (Coef.) and two-sided *p* values above each graph indicate model estimates for cases versus controls at baseline and those above each subsequent visit indicate model estimates for difference relative to baseline within the cases.

During hospitalisation, we identified a higher capacity for immune cells to bind to fluorescently-labelled *Escherichia coli*-coated bioparticles (bacterial binding capacity) as a dominant feature of the anti-bacterial immune function of cases relative to controls^21^. We also provided cross-sectional evidence that bacterial binding capacity was negatively associated with the time after admission when blood samples were collected (i.e., children had had a longer period of rehabilitation), suggesting that this feature of SAM immune function may normalise towards that of healthy controls over time^21^. Here we tested that hypothesis using longitudinal bacterial binding data. We first evaluated longitudinal flow cytometry data to determine whether the cell types that bound to *E. coli* bioparticles (*E. coli* + ) varied with time post-discharge using a combination of t-distributed stochastic neighbour embedding (tSNE) and FlowSOM analyses (Fig. 3A). We identified 8 FlowSOM clusters (Supplementary Fig. 8). Based on the surface markers used for immune cell phenotyping (Lineage (CD3, CD19, CD20, CD56), CD66b, CD14, CD16 and HLA-DR), cluster 2 corresponded to a mixed monocyte phenotype (CD14^+/lo^ CD16^+/lo^), cluster 3 to a neutrophil phenotype (SSC^hi^CD66b + CD16 + ), and clusters 7 and 8 to a mixed lymphocyte phenotype (SSC^lo^Lineage+HLA-DR + /-). Clusters 1, 4, 5 and 6 did not clearly correspond to an identifiable immune cell phenotype. Longitudinal tSNE plots demonstrate that, whilst clusters 2, 3, 7 and 8 are all evident in *E. coli*+ leucocytes from controls, lymphocyte clusters 7 and 8 are relatively less abundant than other clusters in cases at baseline and discharge, and incrementally increase in abundance at 12, 24 and 48 weeks post-discharge (Fig. 3A). Thus, innate immune cells (monocyte cluster 2 and neutrophil cluster 3) are the dominant *E. coli*-binding cells at early stages of SAM, becoming less dominant with time post-discharge. We then used unadjusted mixed regression models of longitudinal proportions of *E. coli*+ immune cell types by SAM status and visit code, including child and case/control status as random effects to quantify these dynamics. Cases had higher overall proportions of *E. coli*+ leucocytes, lymphocytes, monocytes and neutrophils at baseline compared to controls, but *E. coli*+ proportions of all cell types decreased post-discharge relative to baseline proportions in the cases (Fig. 3B, Supplementary Fig. 9; and Supplementary Table 10). The amount of *E. coli* binding per cell type, measured by the mean fluorescence intensity (FI), increased with time post-discharge for total leucocytes and lymphocytes, but declined with time post-discharge for monocytes and neutrophils (Fig. 3C; and Supplementary Table 11). Similar to the tSNE analysis, these data suggest that bacterial binding capacity and bacterial binding by innate immune cell types, in particular, declines as cases recover from SAM; this appears to coincide with restoration of lymphocytes with high per-cell bacterial binding intensity (mean FI) at later post-discharge timepoints.

### Monocyte activation and cytokine secretion normalise over the course of nutritional rehabilitation from SAM

**Fig. 4:**
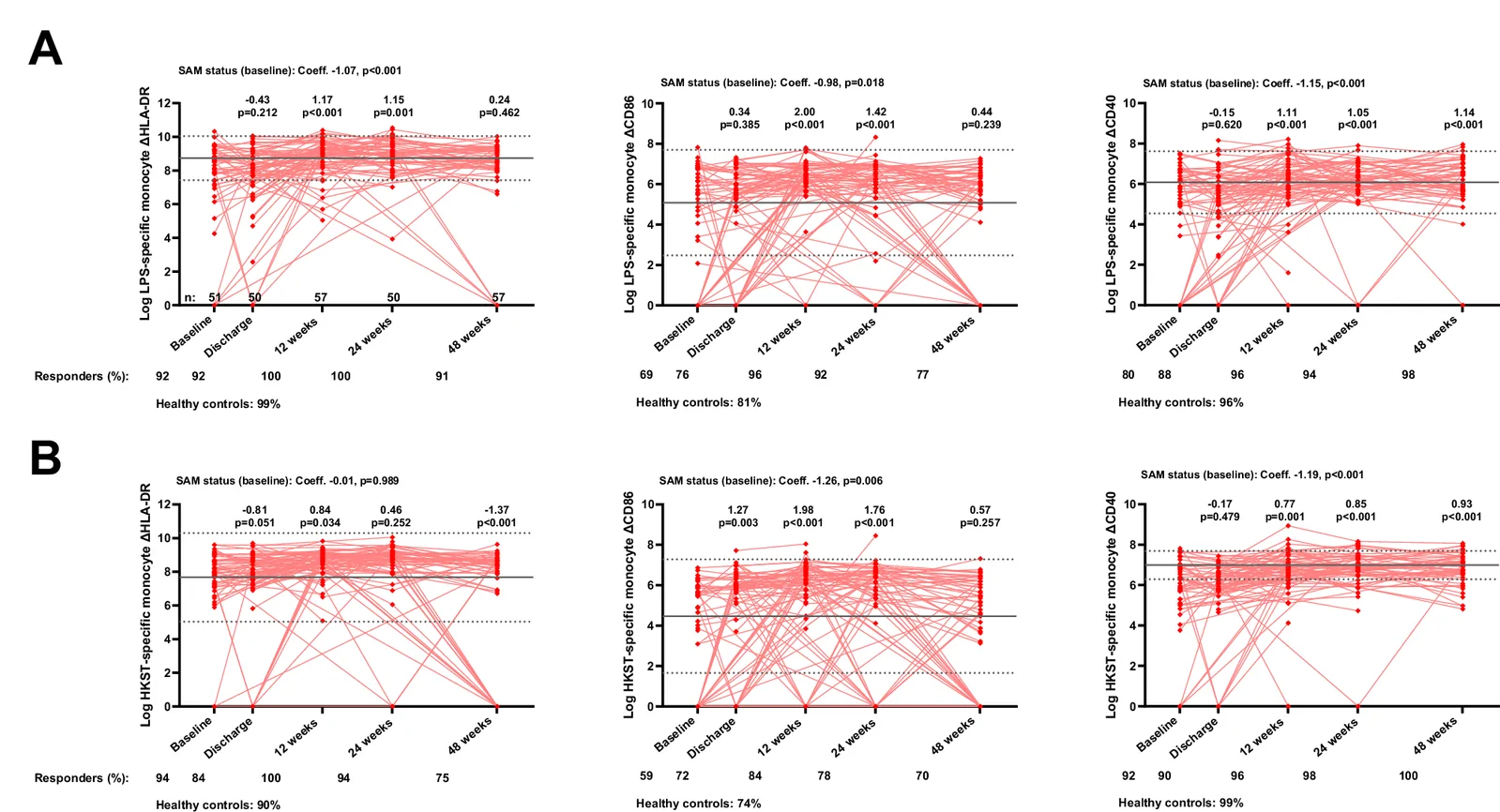
Capacity of blood monocytes to mature in response to bacterial antigen challenge normalises with time after hospitalisation with SAM. Whole blood from each child (SAM cases *n* = 83, healthy control *n *= 73) was cultured for 24 h without stimulus, with LPS and with HKST. Cultured immune cells were phenotyped by flow cytometry and median FI in unstimulated cultures were subtracted from those in antigen-stimulated cultures to give LPS- and HKST-specific responses (Δ). Spaghetti plots of longitudinal log(*n *+ 1)-transformed (**A**) LPS-specific HLA-DR, CD86, and CD40 and (**B**) HKST-specific HLA-DR, CD86, and CD40 expression (median FI) on total monocytes (Lineage-CD66b-HLA-DR + ). Red lines link longitudinal responses of the same child in the SAM group. Mean and standard deviation are indicated in grey for the healthy control group measured at baseline only. **A**, **B** Results of unadjusted mixed regression models of log(*n *+ 1) transformed Δmedian FI by baseline SAM status, visit and the interaction between SAM status and visit as fixed effects and participant identifier and SAM status as random effects are indicated. Coefficients (Coef.) and two-sided *p* values above each graph indicate model estimates for cases versus controls at baseline and those above each subsequent visit indicate model estimates for difference relative to baseline within the cases. Percentage of children at each visit who had a detectable response to LPS or HKST after subtraction of background values in unstimulated (negative control) cultures (responders) is indicated under each plot.

In addition to binding to bacteria, key functions of innate anti-bacterial immune function include upregulation of antigen presenting and co-stimulatory capacity to activate the adaptive immune system and release of pro-inflammatory cytokines and antimicrobials. We used unadjusted mixed regression models of median FI of surface marker expression (HLA-DR, CD86, CD40) by monocytes after 24 h whole blood culture with LPS (Fig. 4A; Supplementary Table 12) or HKST (Fig. 4B; and Supplementary Table 13) to characterise their relationships with case/control status and visit, including child and SAM status as random effects. The capacity of monocytes to upregulate HLA-DR, CD86 and CD40 expression in response to both LPS and HKST (after subtraction of background levels of expression in unstimulated cultures (Δ)) was significantly lower in cases relative to controls at baseline. Post-discharge, LPS- and HKST-induced monocyte activation marker expression was higher at all visits (indicated by positive coefficients) compared to cases at baseline; these patterns were significant for LPS-induced HLA-DR and CD86 at 12 and 24 weeks and CD40 at 12, 24 and 48 weeks (Fig. 4A), and for HKST-induced CD86 at discharge, 12 and 24 weeks, and HLA-DR and CD40 at 12, 24 and 48 weeks (Fig. 4B).

**Fig. 5:**
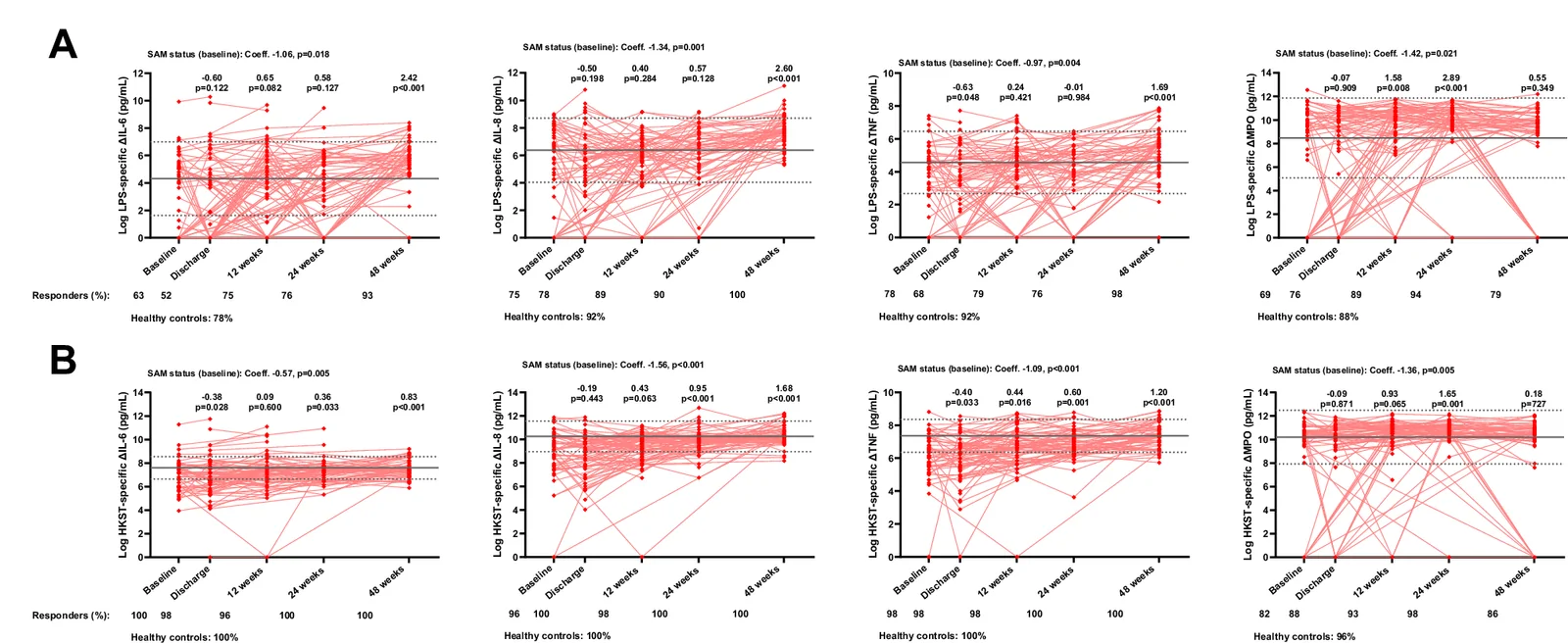
Capacity of blood monocytes to secrete pro-inflammatory cytokines in response to bacterial antigen challenge normalises with time after hospitalisation with SAM. Whole blood from each child (SAM cases *n* = 83, healthy control *n *= 73) was cultured for 24 h without stimulus, with LPS and with HKST. Soluble proteins were quantified in culture supernatants by ELISA and concentrations in unstimulated cultures were subtracted from those in antigen-stimulated cultures to give LPS- and HKST-specific responses (Δ). Spaghetti plots of longitudinal log(n + 1)-transformed (**A**) LPS-specific IL-6, IL-8, TNF, and MPO and (**B**) HKST-specific IL-6, IL-8, TNF, and MPO concentrations (pg/mL) in whole blood culture supernatants. Red lines link longitudinal responses of the same child in the SAM group. Mean and standard deviation are indicated in grey for the healthy control group measured at baseline only. **A**, **B** Results of unadjusted mixed regression models of log(n + 1) transformed Δpg/mL by baseline SAM status, visit and the interaction between SAM status and visit as fixed effects and participant identifier and SAM status as random effects are indicated. Coefficients (Coef.) and two-sided *p* values above each graph indicate model estimates for cases versus controls at baseline and those above each subsequent visit indicate model estimates for difference relative to baseline within the cases. Percentage of children at each visit who had a detectable response to LPS or HKST after subtraction of background values in unstimulated (negative control) cultures (responders) is indicated under each plot.

Longitudinal patterns of LPS- and HKST-induced cytokine and MPO secretion quantified in whole blood culture supernatants by ELISA were similar to those for upregulation of monocyte activation markers. All responses (TNF, IL-6, IL-8 and MPO) to LPS (Fig. 5A; and Supplementary Table 12) and HKST (Fig. 5B; and Supplementary Table 13) were lower in SAM cases than controls at baseline, remained similar or lower than baseline responses at the discharge visit (indicated by negative coefficients), but increased post-discharge (indicated by positive coefficients). Collectively, these data suggest that impairment in monocyte activation marker expression and mediator secretion in response to bacterial antigen stimulation during hospitalisation for SAM is restored with time post-discharge.

### Bacterial binding capacity and bacterial antigen-induced cytokine secretion have reciprocal dynamics during post-discharge recovery from SAM

**Fig. 6:**
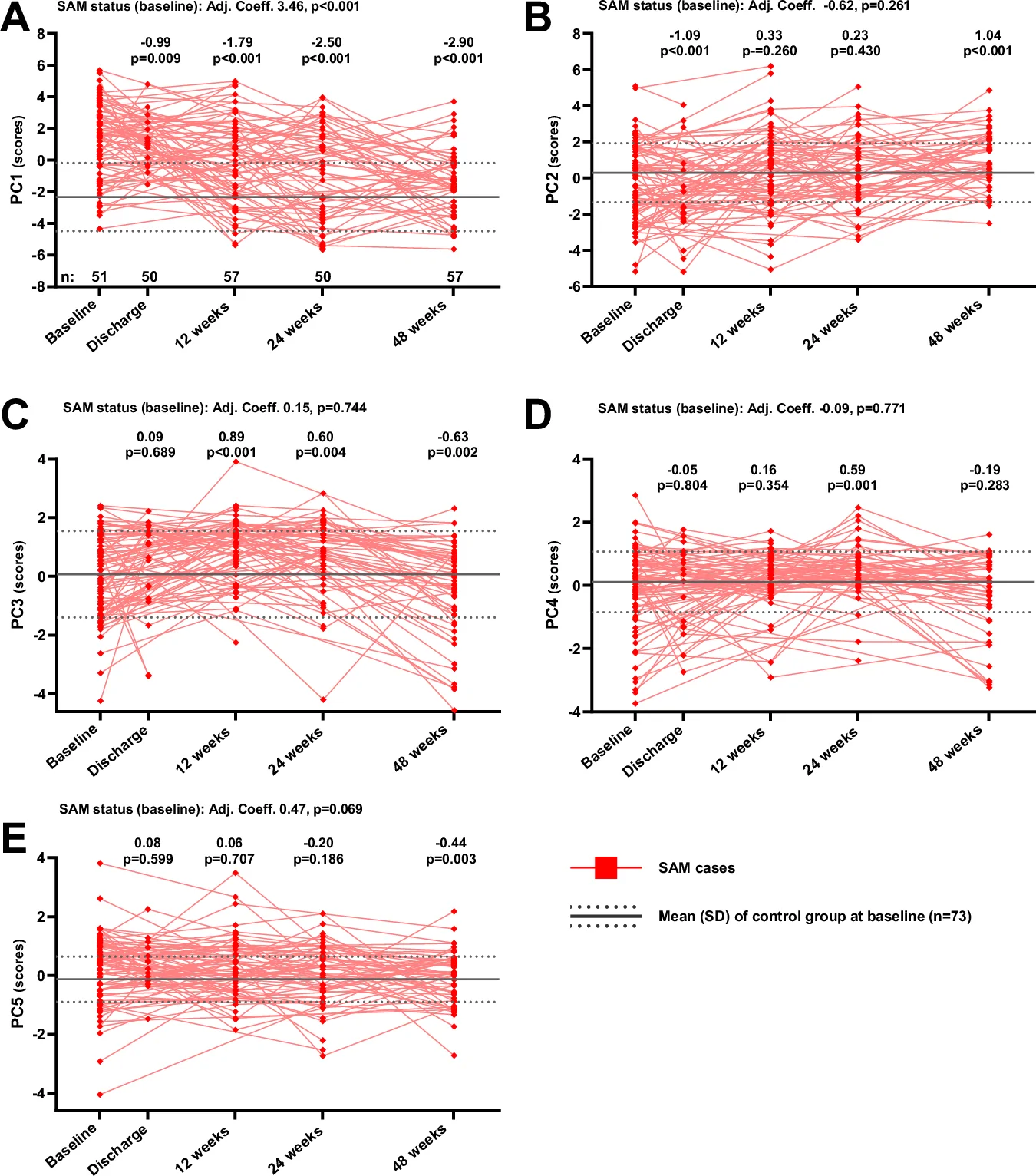
Innate immune cell bacterial binding capacity decreases whilst pro-inflammatory cytokine responses to bacterial antigens increase over the course of post-discharge recovery from complicated SAM. PC scores for previously identified patterns of anti-bacterial immune cell function in SAM cases and healthy controls (Fig. 1B^21^) were estimated for all follow up visits via post-estimation. Spaghetti plots of longitudinal scores for (**A**) PC1(Bacterial binding), (**B**) PC2 (Inflammatory cytokines), (**C**) PC3 (Monocyte activation), (**D**) PC4 (MPO response), and (**E**) PC5 (Neutrophil function) in the longitudinal SAM cohort (*n *= 83). Red lines link longitudinal responses of the same child in the SAM group. Mean and standard deviation are indicated in grey for the healthy control group measured at baseline only (*n* = 73). **A**–**E** Results of multivariable mixed regression models of PC scores by baseline SAM status, visit, the interaction between SAM status and visit, sex, age group at enrolment, country of recruitment, HIV status, cerebral palsy, baseline oedema and WHZ as fixed effects and participant identifier and SAM status as random effects are indicated. Adjusted coefficients (Adj. Coef.) and two-sided *p* values above each graph indicate model estimates for cases versus controls at baseline and those above each subsequent visit.

Young children convalescing after an episode of complicated SAM experience dynamic recovery patterns in the first year after discharge from hospital, as we have previously demonstrated for mortality, morbidity, body composition, nutritional status, clinic visits and hospital readmissions^2,3,13,36^. We hypothesised that these factors may also influence post-discharge patterns of rehabilitation in the 5 key domains of anti-bacterial innate immune function identified by PCA of cross-sectional immune function data (Fig. 1B). We therefore applied the same PCA approach to generate scores for PC1-5 to longitudinal immune function at discharge, 12, 24 and 48 weeks post discharge using post-estimation. We fitted mixed linear regression models, adjusted for characteristics known to influence post-discharge mortality, morbidity and nutritional recovery (i.e., sex, age group at enrolment, country of recruitment, HIV status, cerebral palsy, baseline oedema and baseline WHZ), to characterise changes in these patterns over time. The longitudinal case cohort had higher PC1 (bacterial binding) scores relative to controls at baseline (Adj. Coef. 3.46, 95%CI: 2.14, 4.77, p < 0.001) and, within the cases, scores incrementally reduced relative to baseline from discharge onwards (-0.99, 95%CI: -1.73, -0.25, p = 0.009; 12 weeks -1.79, 95%CI: -2.50, -1.07, *p* < 0.001; 24 weeks -2.50, 95%CI: -3.22, -1.77, p < 0.001; 48 weeks -2.90, 95%CI: -3.60, -2.19, *p* < 0.001; Fig. 6A), but remained significantly higher than those of controls across all timepoints. PC2 (pro-inflammatory cytokine response) scores tended to be lower for cases than controls at baseline (-0.62, 95%CI: -1.70, 0.46, *p *= 0.261), and were significantly lower than baseline scores for cases at discharge (-1.09, 95%CI: -1.68, -0.50, *p* < 0.001), before increasing with time post-discharge to be significantly higher than baseline at 48 weeks post-discharge in the cases (12 weeks 0.33, 95%CI: -0.24, 0.89, *p* = 0.260; 24 weeks 0.23, 95%CI: -0.35, 0.81, *p* = 0.430; 48 weeks 1.04, 95%CI: 0.48, 1.61, *p* < 0.001; Fig. 6B; and Supplementary Table 14). PC3 (monocyte activation) and PC4 (MPO release) scores did not significantly differ between cases and controls at baseline, nor did they have a consistent trajectory relative to baseline within the cases post-discharge (Fig. 6C, D; and Supplementary Table 14). PC5 (neutrophil response) scores tended to be higher for cases than controls at baseline (0.47, 95%CI: -0.04, 0.98, *p* = 0.069), remained similar to baseline scores at discharge, 12 and 24 weeks, but reduced to be significantly lower than baseline scores at 48 weeks post-discharge in the cases (-0.44, 95%CI: -0.73, -0.15, *p* = 0.003; Fig. 6E; and Supplementary Table 14). Linear predictions for each PC from the adjusted mixed models are plotted for cases and controls in Supplementary Fig. 10. Collectively, these results indicate that the abnormally high bacterial binding capacity (PC1) of cases during hospitalisation normalises towards that seen in controls with time post-discharge but remains distinct at all timepoints. In contrast, the abnormally low pro-inflammatory cytokine response (PC2), most evident at discharge, increases towards a healthy range post-discharge.

### Children who nutritionally recover from SAM continue to have a distinct innate immune response to bacteria

**Fig. 7:**
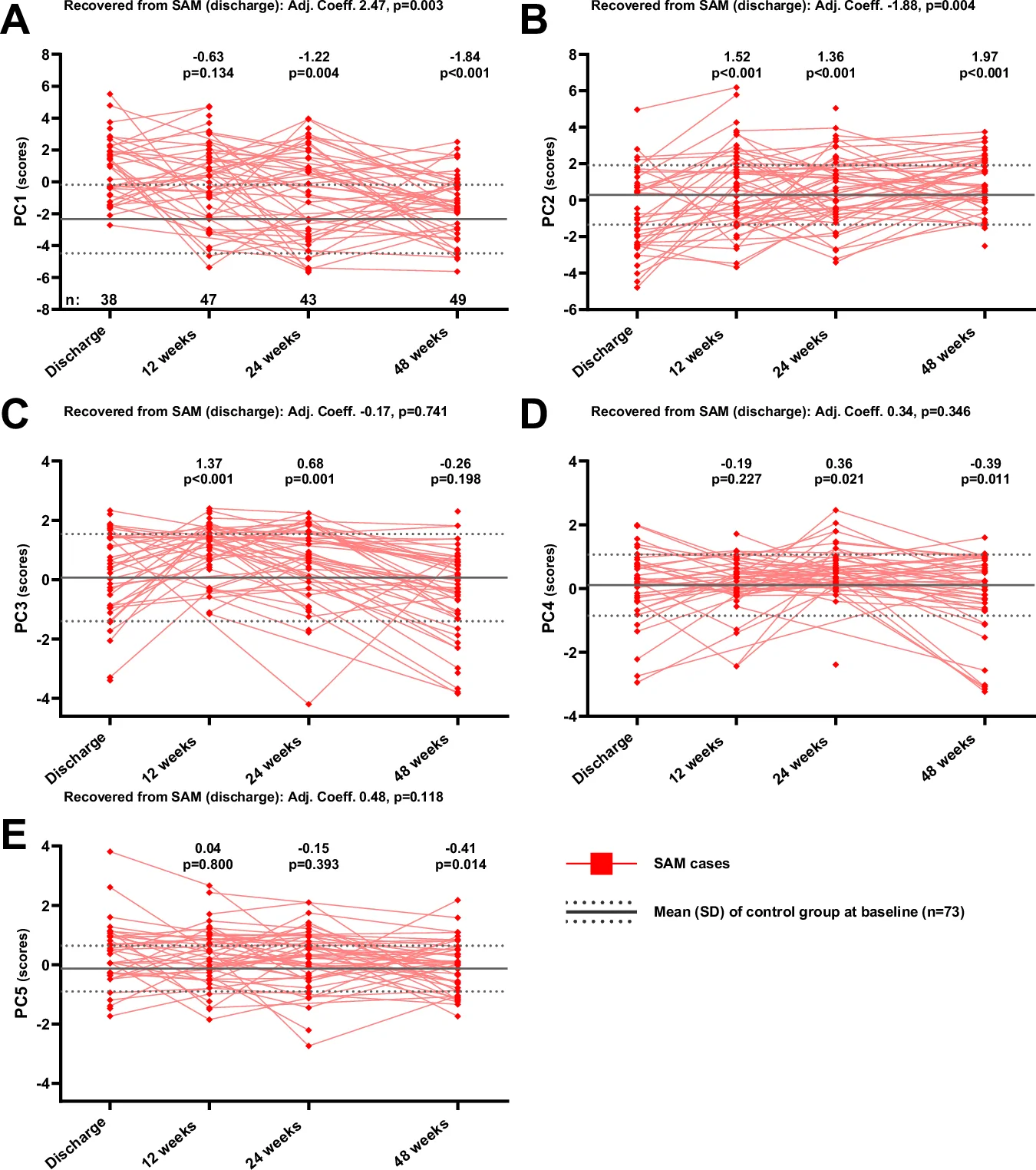
Anti-bacterial innate immune cell function of children hospitalised with complicated SAM continues to be distinct from that of healthy controls even after recovery of a healthy nutritional status. Spaghetti plots of longitudinal scores for (**A**) PC1(Bacterial binding), (**B**) PC2 (Inflammatory cytokines), (**C**) PC3 (Monocyte activation), **D** PC4 (MPO response), and (**E**) PC5 (Neutrophil function) in children from the longitudinal SAM cohort who were adequately-nourished according to their WHZ at discharge (*n* = 38), 12 (*n* = 47), 24 (*n *= 43) and, 48 weeks post-discharge (*n *= 49). Red lines link longitudinal responses of the same child in the SAM group. Mean and standard deviation are indicated in grey for the healthy control group measured at baseline only (*n* = 73). (**A**-**C**) Results of multivariable mixed regression models of PC scores by baseline SAM status, visit, the interaction between SAM status and visit, sex, age group at enrolment, country of recruitment, HIV status, cerebral palsy, baseline oedema and WHZ as fixed effects and participant identifier and SAM status as random effects are indicated. Adjusted coefficients (Adj. Coef.) and two-sided *p* values above each graph indicate model estimates for cases who had recovered versus controls at discharge and those above each subsequent visit indicate model estimates for difference relative to discharge within the cases.

Our third hypothesis was that complicated SAM would leave an imprint on anti-bacterial innate immune function even after a child has made a full nutritional recovery. We repeated the adjusted mixed linear regression model analyses of PC1-5 scores in only cases who were well-nourished (WHZ > -2) at each of the follow-up visits. We found that cases who had recovered by the time of discharge continued to have distinct scores for PC1 (Adj. Coef. 2.47, 95%CI: 0.84, 4.11, *p* = 0.003; Fig. 7A) relative to controls and showed a similar pattern of incrementally decreasing scores at 12, 24 and 48 weeks post-discharge to those seen including cases with SAM and MAM. Thus, bacterial binding capacity continues to normalise towards that of controls over time, even after a child has returned to a healthy weight-for-height range. PC2 scores for the recovered cases were significantly lower than those of controls at discharge (-1.88, 95%CI: -3.17, -0.59, *p* = 0.004; Fig. 7B) and increased relative to discharge scores at 12, 24 and 48 weeks. PCs 3, 4 and 5 scores of recovered cases did not significantly differ from those of controls at discharge (Fig. 7C–E; Supplementary Table 15). Linear predictions for each PC from the adjusted mixed models of recovered cases and controls are plotted in Supplementary Fig. 11. These analyses provide further evidence that restoration of anti-bacterial innate immune cell function may lag behind nutritional recovery among children recovering from SAM.

## Discussion

This study demonstrates that basal anti-bacterial pro-inflammatory cytokine responses of children hospitalised with complicated SAM are associated with subsequent ponderal growth gains, that differences in anti-bacterial innate immune cell function persist throughout 48 weeks of post-discharge follow-up, and remain distinct from controls even among children who are no longer wasted. Thus, delayed recovery of anti-bacterial innate immune cell functions may be an underlying factor in the susceptibility of both wasted and non-wasted children to infectious mortality and morbidity in the year following their original hospitalisation with complicated SAM^2,3,12^. Our work builds on the growing evidence that levels of circulating inflammatory mediators are profoundly dysregulated during hospitalisation for complicated SAM and highest in children who experience the worst clinical outcomes pre- and post-hospital discharge (mortality, nutritional relapse and hospital readmission)^18–20,24,37^. Such patterns imply associated differences in immune cell function for which data is far more limited. We have previously observed that cases within the IMMUNO-SAM cross-sectional cohort have higher basal bacterial binding capacity than controls but lower monocyte activation and pro-inflammatory cytokine responses to bacterial antigens in vitro^21^; we now demonstrate that these basal differences may have underappreciated implications for mortality and morbidity in the year post-discharge^2,3,12^.

We did not find strong evidence to support an association between inpatient immune function and subsequent clinic visits nor with severe adverse clinical outcomes (death, hospital readmission or persistent SAM) over the 48 week follow-up period. However, cases who had the greatest capacity to produce pro-inflammatory cytokines in response to bacterial antigens during hospitalisation also made the greatest ponderal growth gains (ΔMUAC, ΔWHZ). This is consistent with our previous cross-sectional observation that PC2 scores were positively correlated with MUAC at hospital admission^21^. Anti-bacterial innate immune cell function and wasting severity therefore appear to be interlinked in children with SAM during and after hospital discharge.

During the inpatient period, we found that monocytes and neutrophils from cases had a greater capacity to bind bacteria in vitro than those of controls^21^. In contrast, the capacity of monocytes to upregulate surface activation markers and release pro-inflammatory cytokines in response to bacterial antigens was lower in cases than controls^21^. Here we found that bacterial binding capacity declines whilst anti-bacterial pro-inflammatory cytokine responses increase incrementally towards the healthy reference range over 48 weeks of follow-up, showing capacity for immune restoration. However, bacterial binding capacity, in particular, remained distinct from healthy controls throughout the follow-up period and this was also true of cases who had nutritionally recovered. Our observations are consistent with our previously reported cross-sectional evidence that children sampled later during their hospital stay had healthier bacterial binding capacities than those sampled on the day of acute admission^21^. A previous pilot study of blood leucocyte function among children admitted with SAM in Mexico (*n* = 10) also found that phagocytic function, reactive oxygen species (ROS) production and lymphocyte numbers were higher at discharge compared to admission^38^. Circulating dendritic cells from children hospitalised with complicated SAM in Zambia (*n* = 27) were also higher in number, LPS-specific HLA-DR expression and unstimulated IL-12 secretion at discharge than at admission^39^. However, it had not been previously demonstrated how such anti-bacterial functions might change in the longer term after discharge. Given the now well documented evidence that heightened levels of “sepsis-like” systemic pro-inflammatory cytokines are associated with mortality in children hospitalised with complicated SAM^18–20,24,37^, it is plausible that dampened responsiveness of innate immune cells to bacterial antigen stimulation might protect against immunopathology in the short-term, whilst gradual return of cytokine responsiveness would restore defence against new infections, as has been observed in sepsis. However, bacterial binding capacity has not been previously evaluated in SAM. In murine models, monocytes from protein deficient animals uptake more microbes and pathogen-associated molecular patterns (PAMP) in the periphery due to increased trafficking between the site of infection/PAMP injection and the lymph nodes^40^. Similarly, in gram negative sepsis, human monocytes undergo a functional shift away from cytokine production in response to LPS and in favour of phagocytosis and bactericidal functions^41^. Were a similar dynamic change in monocyte function and trafficking to occur in children with SAM, this could be beneficial in terms of infection containment, as we have previously posited for the inpatient period^21^. However, it is also possible that such changes could accelerate dissemination of infections to secondary lymphoid organs if microbes are not efficiently killed after uptake by monocytes. Whilst the longitudinal patterns we observe suggest that the bacterial binding capacity of nutritionally recovered children is more similar to healthy controls post-discharge than at baseline, we did not evaluate monocyte bactericidal capacity meaning that it remains to be determined whether this characteristic of immune function in children with complicated SAM helps or hinders their recovery.

Differences in longitudinal anti-bacterial innate immune cell function reflect both cell-intrinsic and compositional changes in circulating immune cell populations. Total monocytes were proportionally lower in cases versus controls at baseline and throughout the follow-up period. Within the monocyte population, higher proportions of intermediate and lower proportions of non-classical subsets at baseline decreased and increased respectively during follow-up. These changes suggest that myelopoiesis in the bone marrow may not be able to keep up with demand for circulating monocytes which appear to be differentiating from classical to intermediate subsets and potentially extravasating into peripheral tissues more rapidly in children recovering from SAM. Our previously reported plasma biomarker data support such a hypothesis in principle; circulating levels of cell adhesion molecules associated with immune cell interactions with the vascular endothelium (VCAM-1, P-selectin, L-selectin) were significantly elevated in cases relative to controls throughout the HOPE-SAM follow-up period^24^. Murine models of malnutrition in which dietary protein or overall food intake are restricted suggest that monocytes traffic more rapidly to the spleen^40^, and myelopoiesis^27,30^ and granulopoiesis^28^ in response to bacterial challenges are perturbed in the bone marrow relative to mice fed unrestricted diets. One study found that refeeding (10% extra food by weight daily for 2 weeks) dietary-restricted mice before returning them to an *ad libitum* diet for 6-8 weeks restored body weight and CD8 + T cell IFNγ and TNF responses to OVA antigen and reversed lymphoid organ atrophy (also recognised as a feature of human underutrition^42–44^) but failed to restore circulating neutrophil or myeloid precursor numbers or murine capacity to contain *Listeria monocytogenes* infection in vivo^27^. Changes in the bone marrow structure such as depletion of mesenchymal stem cells and myeloid precursors, increased adiposity and accumulation of pro-inflammatory signals observed in mice with dietary deficiency followed by bacterial challenge^28,30^, could plausibly contribute to the delayed restoration of the blood monocyte pool and abnormal monocyte and neutrophil functions we observe in children recovering from SAM. We have previously reported that plasma concentrations of granulocyte colony-stimulating factor (G-CSF), which promotes expansion of bone marrow granulocyte and stem cell populations, also tended to be lower (though not significantly so) in cases than controls throughout follow-up^24^. However, these mechanisms have not been directly explored in children with SAM. Investigation in relevant primary tissues (endothelium and bone marrow) and/or laboratory models are needed to explore these functional paradigms in human undernutrition were, unlike in diet-only animal models, dietary deficiencies coincide with dysbiosis and infections^45,46^. More detailed evaluation of blood neutrophil and myeloid precursor subsets in children recovering SAM is also warranted considering recent evidence that neutrophil immaturity and mitochondrial dysfunction shape responses to LPS challenge in murine dietary deficiency^28^.

Whist our study provides valuable insights into longitudinal immune cell function in children recovering from SAM and is the largest of its kind to date, our results should be interpreted in the context of several study limitations. First, given the requirement for a blood sample of ≥2 mL which is not possible from the most critically unwell children at hospital admission, children included in our cohort were healthier than the typical SAM inpatient and experienced lower morbidity and mortality than that of the HOPE-SAM cohort as a whole^2,3,13^. As such, incidence of adverse clinical outcomes was low; evaluation of their associations with immune cell function has low statistical power and should be considered exploratory. Furthermore, the longitudinal changes we observe in immune function may underestimate those seen in more extreme cases of complicated SAM. Whilst post-discharge nutritional relapse, mortality and hospital readmission among children recovering from SAM are frequently associated with infections^10–12,47^, we did not have diagnostic data to more directly assess relationships between immune cell function and infection risk. As is typical for hospital-based longitudinal follow-up studies of children recovering from SAM^4,48^, not all cases provided a sample at all visits, and some were lost to follow-up. Such missing data is unlikely to be at random and this bias likely reflects recognised socio-economic challenges faced by affected families, as reported by caregivers of HOPE-SAM participants^49^. Our results are therefore most generalisable to easier to reach children who are likely less affected by barriers to accessing healthcare. Whilst inclusion of relevant confounders as fixed effects and individual participant as a random effect in our mixed effects models go some way to addressing the influence of inter-individual heterogeneity within our cohort, a larger sample size with less missingness would have given greater power to detect longitudinal patterns of immune function and explore previously identified clinical risk groups in more depth^2,3,13^. Although both the SAM and healthy control groups were recruited across a range of ages and seasons, reducing the likelihood that observed differences were driven by systematic age or seasonal bias, we recognise that a potential small effect of age and seasonal variations on healthy immune function cannot be fully excluded^50,51^. As discussed here and previously^21,23,45,46^, there remain many potentially relevant circulating and tissue immune cell types and functions to explore in longitudinal SAM cohorts that were beyond the scope of the current study or emerged after the study was completed. For example, pre-specified blood sampling regimens meant that we did not evaluate immune function between the discharge and 12 week visit, during which the majority of adverse clinical outcomes occurred during HOPE-SAM follow-up^2,3^. Furthermore, distinct pre-specified selection criteria for the IMMUNO-SAM cohort^31^ meant that we did not have sufficient overlap to explore associations between immune cell function and previously-reported longitudinal plasma and stool biomarkers^24^.

Collectively, our study suggests that complicated SAM, similar to other severe acute illnesses requiring hospitalisation (e.g., multi-system inflammatory syndrome in children^52^, severe febrile illness^53–55^, sepsis^34,56^, or all cause hospitalisations^57^), is associated with long-term differences in cellular immune responses against infection which are not resolved by current treatment interventions over 48 weeks of post-discharge follow-up. Current convalescent care for children with SAM includes linkage to community management of acute malnutrition programmes with clinical monitoring and provision of ready-to-use therapeutic food per WHO guidelines^1^. However, interviews with caregivers of HOPE-SAM participants in Zimbabwe, suggest that multiple factors can compromise a family’s ability to access optimal community care^49^. Therefore, context-relevant adjunctive interventions against infections should be considered alongside existing nutritional approaches during the early phase of post-discharge convalescence ( ≤ 12 weeks) when both nutritional and immune rehabilitation are incomplete and the risk of infectious mortality and morbidity is greatest^2,3,47^. Further studies are needed to identify whether interventions targeting innate immune cell function could contribute to a fuller recovery of children after hospitalisation with complicated SAM.

## Methods

### Ethics

Ethical approval for HOPE-SAM was obtained from the University of Zambia Biomedical Research Ethics Committee, and the Medical Research Council of Zimbabwe. The ethics committee of Queen Mary University of London provided an advisory review (QMUL reference: QMREC2015/17). Procedures for IMMUNO-SAM were added as an amendment to the HOPE-SAM protocol and approved on the 18^th^ August 2017 (HOPE-SAM protocol version 3, 10/07/2017). This study adhered to all relevant ethical regulations. Caregivers provided written informed consent for the participation of their children in this study.

### Clinical study design

Design of the IMMUNe function Of children with SAM (IMMUNO-SAM) study, which was nested within the HOPE-SAM longitudinal observational cohort study have been published previously^21,31^. The HOPE-SAM protocol, procedures, and case report forms are available at https://osf.io/29uaw/. Briefly, HOPE-SAM enroled children under 5 years old who were hospitalised for complicated SAM at three hospitals in Lusaka (University Teaching Hospital), Zambia and Harare (Harare Central Hospital and Parirenyatwa Hospital), Zimbabwe between August 2016 and March 2018. SAM was defined per WHO definitions as WHZ  < − 3, MUAC  < 115 mm, nutritional oedema or both for children above 6 months of age, or WHZ  < − 3, nutritional oedema or both for those aged under 6 months^1^. Children of caregivers who provided written-informed consent, were enroled as soon as possible after hospital admission (baseline) and reviewed daily during hospitalisation. Children were discharged from hospital according to WHO-adapted country guidelines, which specify that discharge is appropriate when a child’s medical complications, including oedema, are resolving, they are clinically well, alert and their appetite has returned^1^. Children had follow-up clinical visits with the HOPE-SAM study team at discharge, 2, 4, 12, 24 and 48 week visits after discharge. Biological samples were collected at the baseline, discharge, 12, 24 and 48 weeks visits. Full data on clinical procedures performed at each study visit are reported elsewhere^2,3,31^.

Children aged 6-59 months who were adequately-nourished (WHZ > –1), not admitted to hospital and clinically well (i.e., no symptoms of acute illness or current infections, no underlying chronic gastrointestinal disease) were recruited from the same hospitals as the cases as a context-relevant control group; children were not excluded from the control group on the basis of their HIV status or linear growth (e.g., height-for-age Z score). Biological samples were collected from controls at enrolment (baseline) only. To be eligible for enrolment as a case or control, children needed to have a known HIV status.

IMMUNO-SAM was conducted using longitudinal blood samples (1 mL/kg up to a maximum of 5.4 mL) collected into EDTA-treated endotoxin-free blood collection tubes at the baseline, discharge, 12, 24 and 48 week study visit. Blood was not collected from children known to have severe anaemia (haemoglobin <60 g/L). For inclusion in the IMMUNO-SAM sub-study, HOPE-SAM participants needed to have enroled into HOPE-SAM after ethical approval of IMMUNO-SAM procedures and provided a blood sample ≥ 2 mL during their hospitalisation (cases) or at enrolment (controls). For cases, it was not always possible to collect blood samples on the day of hospital admission due to critical illness, therefore baseline immune function assessment was conducted on the first blood sample ≥2 mL collected from a participant which could be at any time during their hospital stay, as previously reported^21^. Cases and controls were not matched for the IMMUNO-SAM study; statistical analyses included adjustment for characteristics that differed between the two groups at baseline (i.e., age group at enrolment and HIV status; Table 1). Selection of the IMMUNO-SAM cohort is summarised in Fig. 1A. All eligible participants were included in statistical analyses to maximise sample size. All laboratory work for children recruited in Zambia was conducted at TROPGAN laboratories, Lusaka and for those recruited in Zimbabwe at Zvitambo laboratories, Harare.

### Clinical outcomes

Clinical outcomes were defined according to the HOPE-SAM clinical study. Nutritional status was classified according to anthropometry at each visit: adequately nourished (WHZ  ≥ − 2 and/or MUAC ≥ 125 mm), MAM (WHZ between -2 and −3 and/or MUAC of 115–124 mm) or SAM (as above)^1^. The lower nutritional status category was used if WHZ and MUAC classifications differed.

Caregiver-reported symptoms in the previous 2 weeks were recorded at each study visit. Illnesses identified at follow-up visits were managed by study clinicians, with referral to hospital, if needed. Hospital readmission, clinic and hospital visits were caregiver-reported at each study visit and verified using caregivers’ hand-held child health cards where available. Caregiver reported symptoms, hospital admissions, and clinic visits solely for pre-existing congenital or developmental conditions (e.g., congenital heart disease) or due to traumatic injury not plausibly influenced by preceding immunological differences (e.g., burns, fractures) were excluded from analyses for the IMMUNO-SAM study. Full details of all-cause re-admissions have been previously reported^2^.

Vital status of each participant was ascertained by daily inpatient assessment during hospitalisation and caregiver interview, and review of hand-held medical records, where available, at post-discharge follow-up visits. Vital status was determined via phone call where caregivers could not attend study visits.

### Bacterial binding assay

Full details of the bacterial binding assay procedure are reported elsewhere^21^. 100ul of freshly collected undiluted whole blood was added to a pre-prepared cryovial with culture medium only (RPMI 1640 1% v/v penicillin-streptomycin; GIBCO, USA) and to a pre-prepared cryovial with 5 × 10^5^ Alexa Fluor 488-conjugated *E. coli* (K-12 strain) coated bioparticles diluted in culture medium (Life Technologies, UK). Both vials were cultured for 1 h at 37 ⁰C, 6% CO_2_ in the CO2Gen compact atmosphere generation system (Oxoid, UK). After 1 h, cultured cells were treated with 1x FACS lysing and fixative buffer (BD Biosciences, UK) for 10 mins, washed in phosphate-buffered saline (PBS), resuspended in PBS with 10% v/v dimethyl sulfoxide (DMSO) 40% v/v heat-inactivated foetal bovine serum (FBS), and gradually frozen at 1 °C/min to -80 ⁰C using a Mr. Frosty freezing container.

### Whole blood culture with bacterial PAMP

Full details of the whole blood culture procedure are reported elsewhere^21^. 500ul of freshly collected diluted (1 in 3 in culture medium) whole blood was added to pre-prepared culture wells containing medium alone, HKST (final concentration: 1×10^8^cells/mL; Invivogen, UK) and ultrapure *E. coli* LPS (final concentration: 0.25EU/mL; Invivogen, UK). Cultures were incubated for 24 h at 37 ⁰C, 6% CO_2_ using the CO2Gen compact atmosphere generation system (Oxoid, UK). Cell-free culture supernatants were harvested and cryopreserved at -80⁰C. Cultured blood cells were subjected to red blood cell lysis, fixed and cryopreserved as described above.

### Enzyme-linked immunosorbent assay (ELISA)

TNF, IL-6, IL-8 and MPO concentrations were quantified in whole blood culture supernatants by ELISA (Duoset ELISA kits; Biotechne, UK; Catalogue numbers: DY210, DY208 and DY3174; and OptEIA kit; BD Biosciences, Catalogue number: 555220). Concentrations below the assay detection limit (TNF - 15.6 pg/ml, IL-6 - 4 pg/ml, IL-8 - 31.2 pg/ml and MPO - 62.5 pg/ml) were censored at the limit of detection.

### Flow cytometry

Stored bacterial binding assay cell samples were labelled with: Lin (CD3, CD19, CD20, CD56)-APC (10 µL/tube, clones: UCHT1, HIB19, 2H7, 5.1H11; Cat# 363601), CD66b-PerCPCy5.5 (0.5 µL/tube, clone: G10F5; Cat#305108), CD16-APCCy7 (2.5 µL/tube, clone: 3G8; Cat# 302018), CD14-PE (2.5 µL/tube, clone: HCD14; Cat# 325606, all Biolegend, UK) and HLA-DR-PECy7 (2.5 µL/tube, clone: L243 (G46-6); Cat# 560651, BD Biosciences, UK). Whole blood culture cell samples were labelled with: Lin (CD3, CD19, CD20, CD56)-APC, CD66b-APC (0.5 µL/tube, clone: G10F5; Cat# 305118), CD16-APCCy7, CD14-PE, CD86-FITC (5 µL/tube, clone: BU63; Cat# 374204), CD40-PerCPCy5.5 (2.5 µL/tube, clone: 5C3; Cat# 334316, all Biolegend, UK) and HLA-DR-PECy7 (BD Biosciences, UK). Cells were analysed on a 6-colour BD Biosciences FACSVerse flow cytometer (488 nm and 633 nm lasers). Samples were processed for flow cytometry in batches with all visits for an individual participant included in the same batch and each batch including samples from both cases and controls. Compensation controls (beads and cells labelled with a single fluorochrome) and fluorescence-minus-one controls (FMO) were run alongside every sample batch to adjust for spectral overlap and identify positive/negative staining, respectively. Flow cytometry files were analysed using FlowJo version 10.8.1 (FlowJo, LLC, USA); gating strategies implemented for each sample type are shown in Supplementary Fig. 12 and have been previously published^21^.

### Statistical analyses

*E. coli*+ proportions and mean AF488 FI values from bacterial binding assays were analysed without subtraction as there was no background detected in paired cultures without bioparticles. To quantify the inducible response of immune cells to bacterial antigens above background levels in unstimulated cultures, median FI of HLA-DR, CD86 and CD40 on monocytes and soluble mediator concentrations in each participant’s unstimulated whole blood culture were subtracted from those in their LPS- and HKST-stimulated cultures (Δ). Immune function variables were left-skewed (zero-inflated); distributions were suitable for parametric testing after log(*n* + 1)-transformation. Only participants with complete data on bacterial binding, whole blood culture cells and supernatants were included in statistical analyses; participants excluded due to missing data from one assay were assumed to be missing at random.

We conducted factor reduction (PCA) to identify a minimal number of composite variables reflecting the greatest variation in our combined immune function dataset (PC1-5), as previously described^21^. Briefly, all individual log-transformed anti-bacterial innate immune cell function variables for the first inpatient immune function assessment for all cases and controls with complete data were standardised and entered into a single PCA (full variable list in Fig. 1B). Five inpatient PCs were identified to have an eigenvalue > 1. Standardised individual immune function variables with a factor loading ≥0.3 (positive correlation) or ≤-0.3 (negative correlation) for a PC were considered to contribute to the pattern of immune function expressed by scores for that PC. Scores for PC1-5 were predicted for each case at each follow-up visit via post-estimation. The relationship between inpatient immune function and clinical outcomes was evaluated among cases by univariable and then multivariable linear regression of PC scores by clinical group (i.e., cases with versus without a clinical event), adjusting for participant characteristics at admission (sex, age group, oedema status, HIV status, MUAC, cerebral palsy, height-for-age Z score), country of recruitment and SAM status at discharge which were previously identified as predictors of clinical outcomes in HOPE-SAM^2,3,13^. The relationship between immune function and ponderal growth gains was evaluated by univariable and then multivariable linear regression of PC scores by change (Δ) in MUAC and WHZ between baseline and 48 weeks, adjusting for sex, age group at enrolment, HIV status, baseline oedema, cerebral palsy, country of recruitment, SAM status at discharge and baseline MUAC/WHZ.

T-distributed stochastic neighbour embedding (tSNE) plots for all compensated cell surface markers (Lineage, CD14, CD16, CD66b, HLA-DR) were generated for 10,000 singlet leucocyte *E. coli*+ events per visit including equal sampling of 200 events per sample for controls (*n* = 73), and cases at baseline (*n* = 51), discharge (*n* = 50), 12 (*n* = 57), 24 (*n* = 50) and 48 week (*n* = 57) visits using the cytofkit2 package in R (https://github.com/JinmiaoChenLab/cytofkit/). FlowSOM was applied to tSNE to identify clusters of *E. coli*+ cells according to their shared surface marker expression^58^.

To characterise longitudinal changes in immune cell phenotype (unstimulated cultures) and function variables (bacterial binding and response to LPS and HKST), we fitted mixed-effects linear regression models of individual immune function variables by follow-up visit, case/control status and the interaction between visit and case/control status. Such models use Maximum Likelihood to analyse all available data from cases rather than only participants with complete follow-up, thereby reducing potential bias from missing data at one or more visits. A random intercept was included for each child, restricted to cases only, since controls contributed data at a single baseline visit. The models gave coefficients for cases versus controls at the baseline visit and for changes in the case group at each post-discharge visit relative to their response at the baseline visit. We applied the same mixed effects linear regression models to PC1-5 scores, adjusting for variables associated with nutritional recovery: sex, age group at enrolment, HIV status, baseline oedema, cerebral palsy, country of recruitment, and baseline WHZ. Fixed effects were selected for inclusion in adjusted models to reflect imbalance between cases and controls at baseline (age group at enrolment and HIV status; Table 1) and variables that we have previously been found to influence immune function (age group at enrolment, HIV status^21^) and/or clinical outcomes (sex, age group at enrolment, HIV status, baseline oedema, cerebral palsy, country and baseline WHZ^2,3,13,36^) in children with SAM. Models were repeated, including only cases who were adequately nourished at each post-discharge follow-up visit; these models gave coefficients for cases who had recovered by hospital discharge versus controls at the baseline visit and for changes in the case group at each post-discharge visit relative to their response at the discharge visit.

Statistical analyses were planned to address our 3 key hypotheses with primary inference based on coefficients and *p* values from adjusted models for PCs. All analyses were two-sided and we report statistical significance for all analyses at uncorrected *p* values < 0.05.

All statistical analyses were performed using STATA version 18 (StataCorp LLC, USA). Outcome variables were plotted using Prism version 10 (GraphPad Software Inc., USA), STATA version 18, and R version 4. Nutritional trajectories were plotted using SankeyMATIC (https://sankeymatic.com/build/).

### Reporting summary

Further information on research design is available in the Nature Portfolio Reporting Summary linked to this article.

## Supplementary information


Supplementary Information
Reporting Summary
Transparent Peer Review file


## Source data


Source Data


## Data Availability

The dataset from the immunology sub-study of HOPE-SAM (IMMUNO-SAM) reported in this article is available at (https://doi.org/10.6084/m9.figshare.32678214). Source Data is provided for each figure. All other data are available in the article and its Supplementary files or from the corresponding author upon request. Source data are provided with this paper.
