# Supplementary material for: Restoration of anti-bacterial innate immune cell function lags behind nutritional recovery among children convalescing from complicated severe acute malnutrition
Source: Nat Commun. 2026 Jul 28;17:9172. doi: 10.1038/s41467-026-75897-7 (PMC13521902; doi:10.1038/s41467-026-75897-7)
Supplement: Supplementary file 1 [file 41467_2026_75897_MOESM1_ESM.pdf]

SUPPLEMENTARY FIGURES

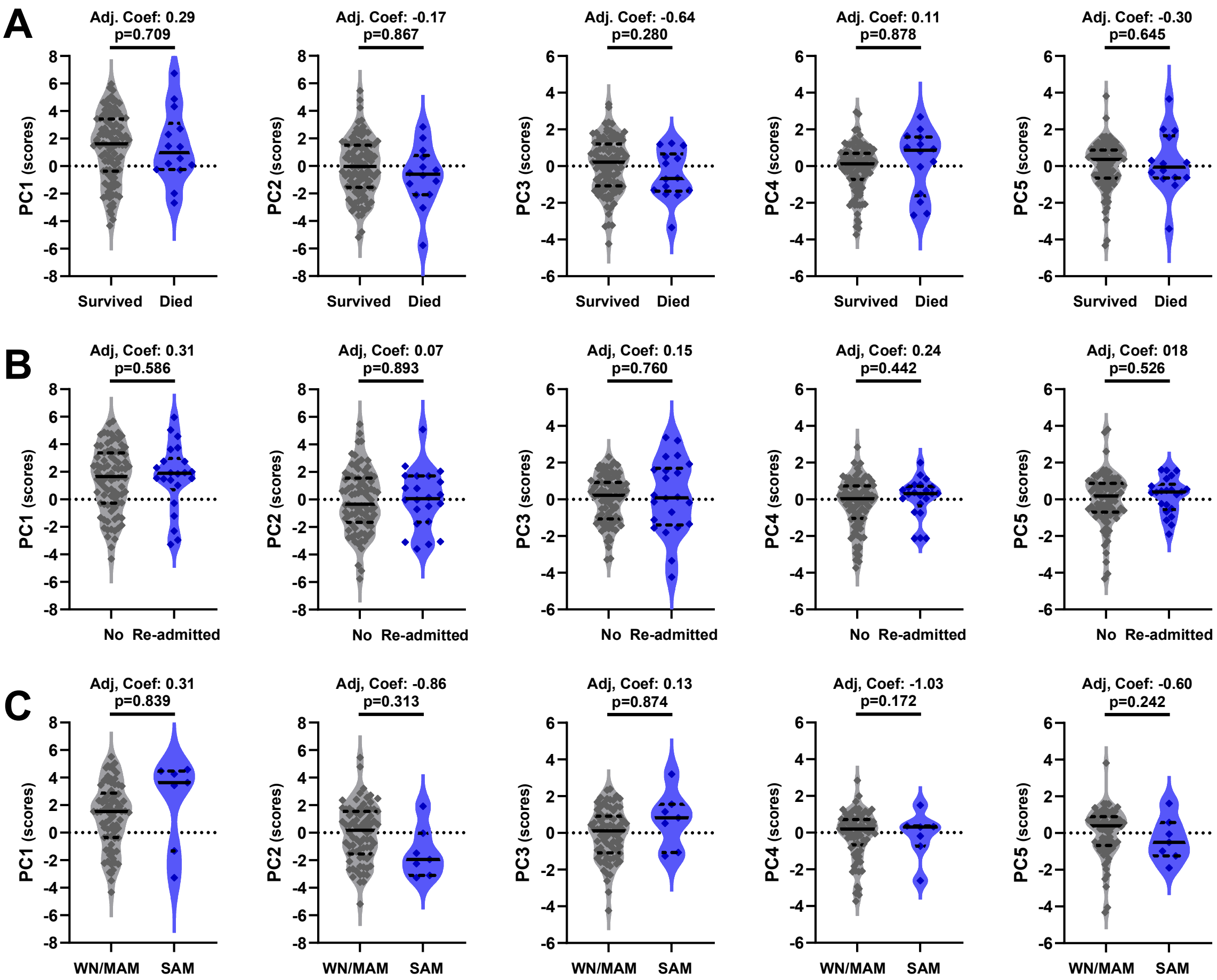

Supplementary Figure 1. No evidence for differences in inpatient anti-bacterial innate immune cell function of children who died, were re-admitted to hospital or had persistent SAM at the 48 week post-discharge visit. Violin plots of inpatient PC1-5 scores between children who (A) died (blue; n=14) versus survived (grey; n=104); (B) were re-admitted to hospital (blue; n=22) versus not re-admitted (grey; n=87); or had persistent SAM (blue; n=7) versus MAM or adequately-nourished at the 48 week study visit (n=78). Results of unadjusted linear regression of PC scores by clinical outcome are indicated by coefficient (Coeff) and p-value. Comparison of inpatient PC scores by combined clinical outcome (death, readmission and/or SAM at 48 weeks), adjusting for relevant confounder variables, is shown in **Figure 2B**.

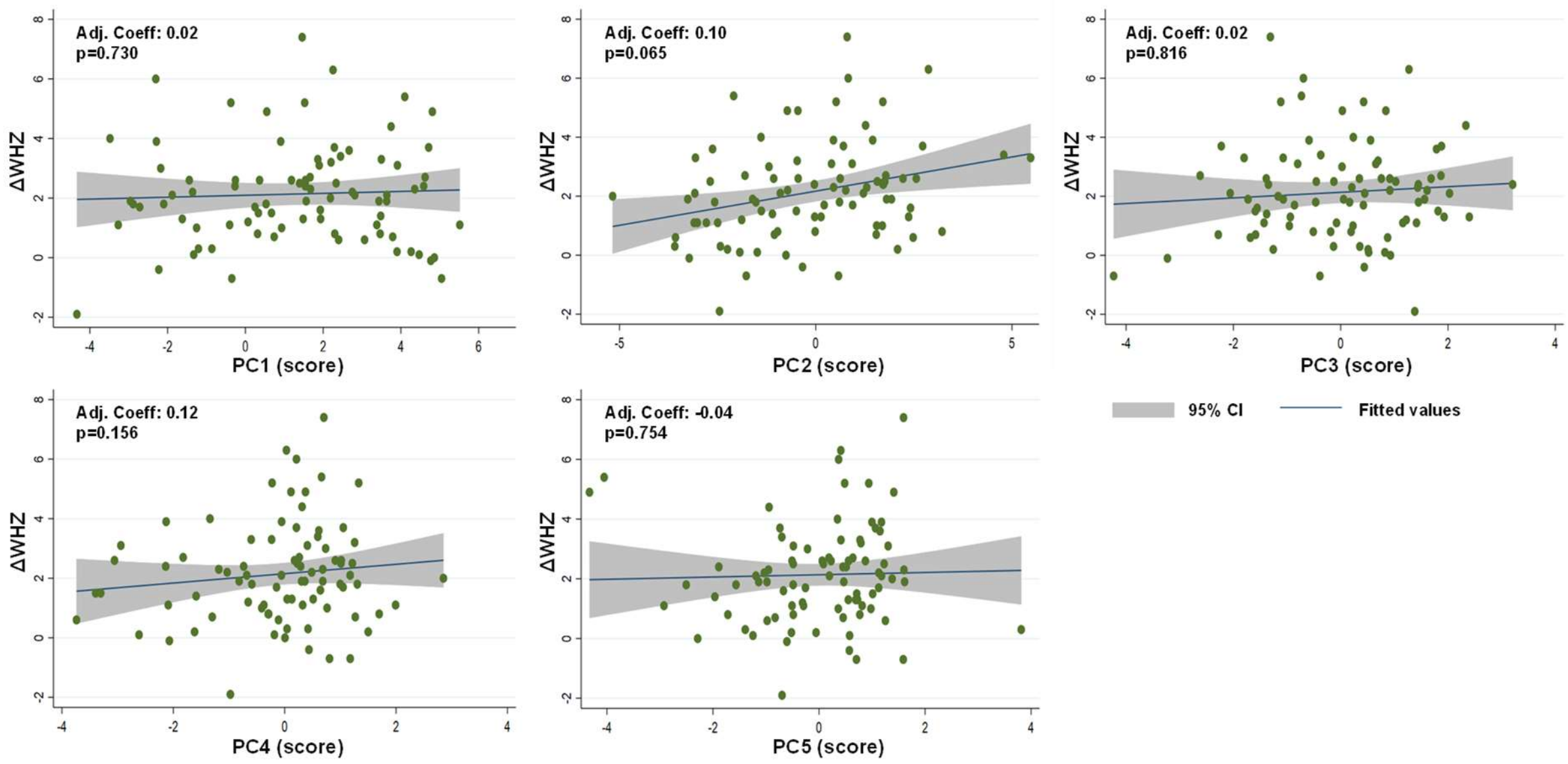

Supplementary Figure 2. Association between inpatient anti-bacterial innate immune cell function and gains in WHZ over the course of recovery. Scatterplots of change (Δ) in WHZ between admission and 48 week visit versus PC1-5 scores of the SAM cases at initial inpatient immune function assessment (n=85). Results of multivariable linear regression of PC scores by ΔWHZ, adjusted for sex, age group at enrolment, HIV status, cerebral palsy, country of recruitment, baseline oedema and WHZ, and SAM status at discharge are indicated by Adj. Coeff, p-value, fitted values and 95% CI.

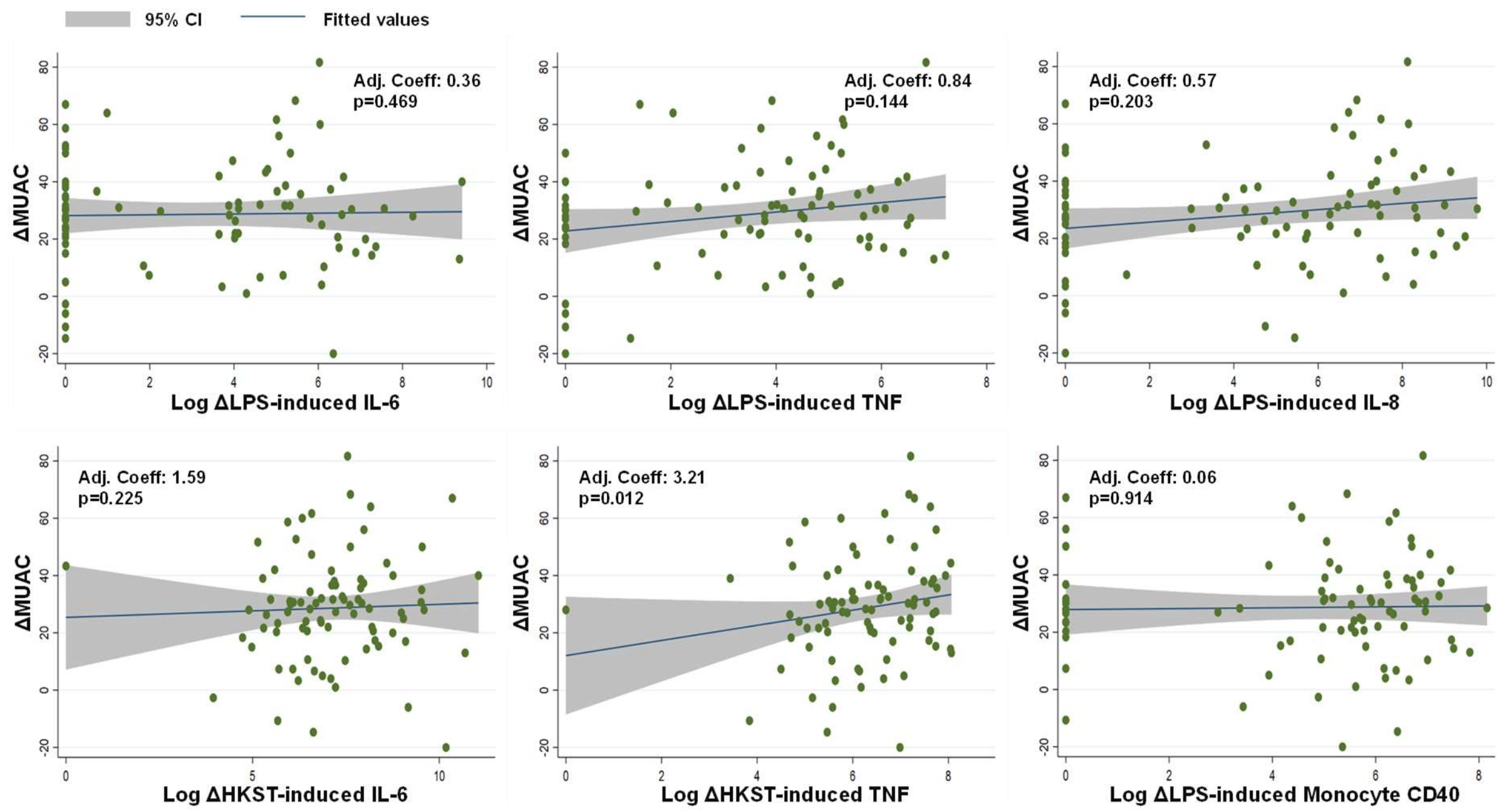

Supplementary Figure 3. Positive association between inpatient HKST-induced TNF and gains in MUAC over the course of recovery. Scatterplots of change (Δ) in MUAC between admission and 48 week visit for the SAM cases at initial inpatient immune function assessment versus their log(n+1) transformed values for each of the individual immune function variables loaded onto PC2 (n=85). Results of multivariable linear regression of PC scores by ΔMUAC, adjusted for sex, age group at enrolment, HIV status, cerebral palsy, country of recruitment, baseline oedema and WHZ, and SAM status at discharge are indicated by Adj. Coeff, p-value, fitted values and 95% CI.

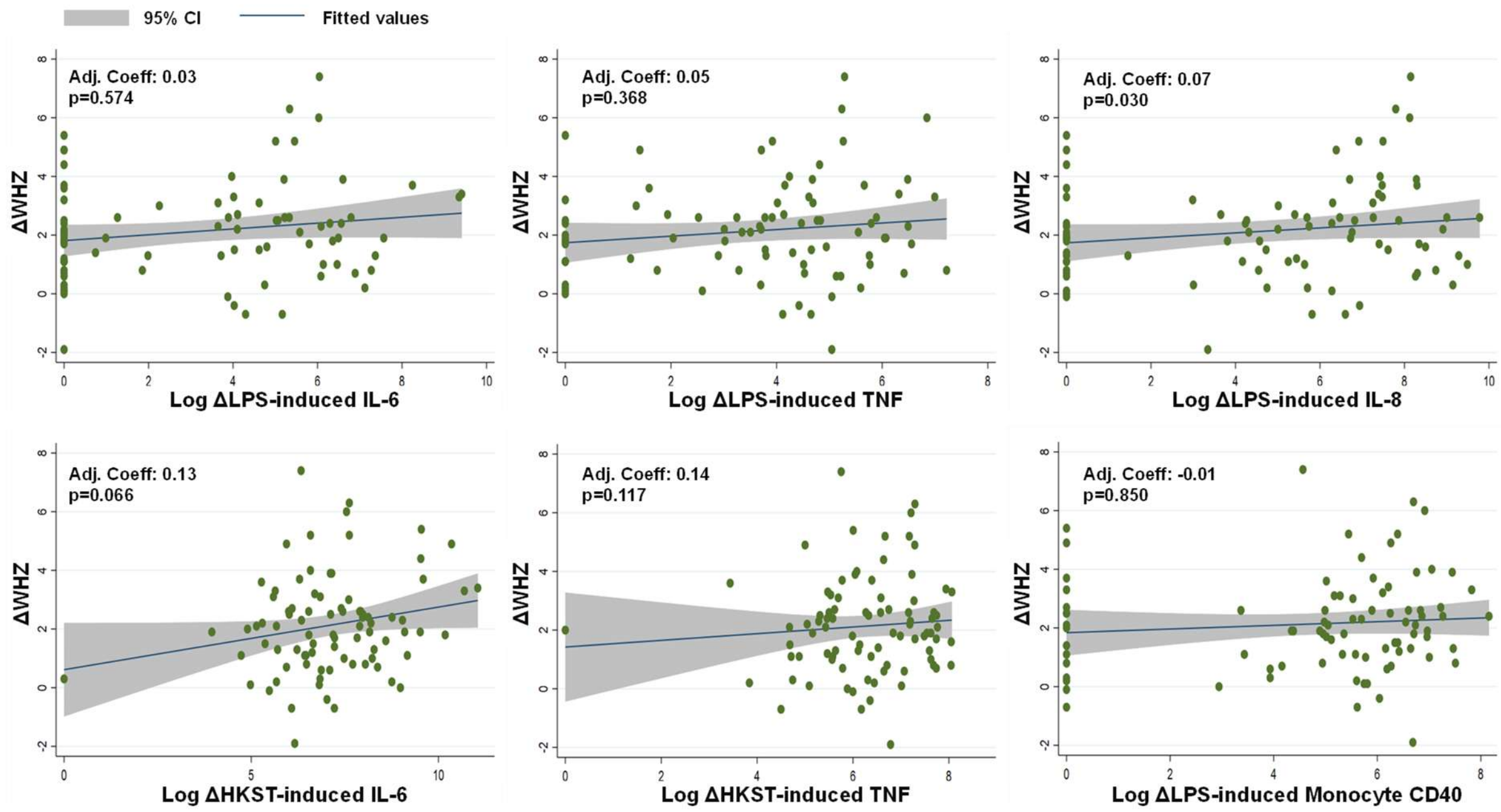

Supplementary Figure 4. Positive association between inpatient LPS-induced IL-8 and gains in WHZ over the course of recovery. Scatterplots of change (Δ) in WHZ between admission and 48 week visit for the SAM cases at initial inpatient immune function assessment versus their log(n+1) transformed values for each of the individual immune function variables loaded onto PC2 (n=85). Results of multivariable linear regression of PC scores by ΔWHZ, adjusted for sex, age group at enrolment, HIV status, cerebral palsy, country of recruitment, baseline oedema and WHZ, and SAM status at discharge are indicated by Adj. Coeff, p-value, fitted values and 95% CI.

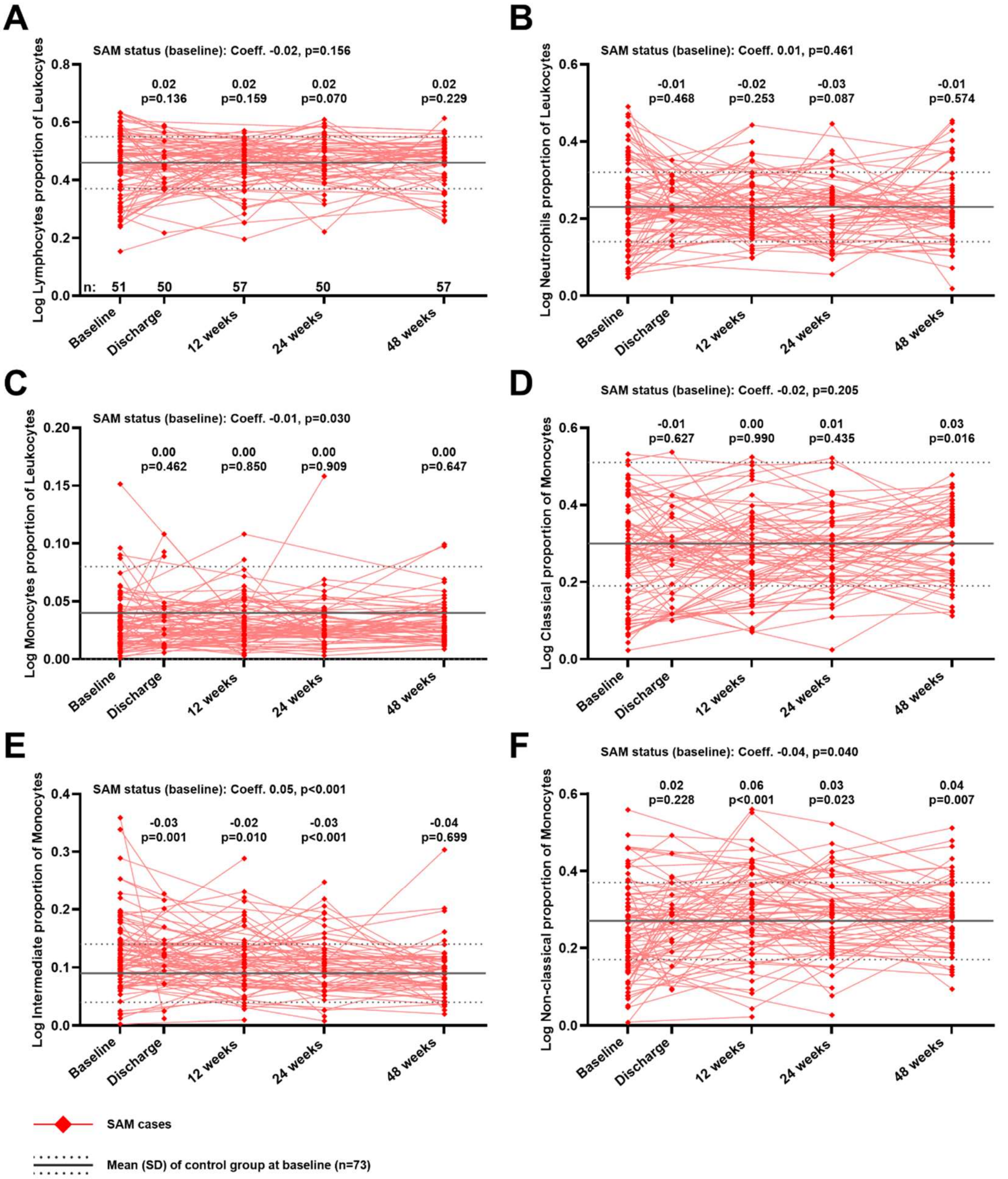

Supplementary Figure 5. Circulating immune cell composition changes over the course of 48 weeks of post-discharge follow-up after hospitalisation with SAM. Spaghetti plots of longitudinal log(n+1) transformed proportions of blood (A) lymphocytes (Lineage+(CD3, CD19, CD20, CD56), (B) neutrophils (Lineage-CD66b+CD16+), and (C) monocytes (Lineage-CD66b-HLA-DR+) of total single leukocytes from unstimulated blood samples. Spaghetti plots of longitudinal proportions of blood (D) classical (CD14+CD16-), (E) intermediate (CD14+CD16+), and (F) non classical ($CD14^{lo}CD16+$) subsets of total monocyte population. Red lines link longitudinal responses of the same child in the longitudinal SAM cohort (n=83). Mean and standard deviation are indicated in grey for the healthy control group measured at baseline only (n=73). Results of unadjusted mixed regression models of log(n+1) transformed proportions by baseline SAM status, visit, the interaction between SAM status and visit as fixed effects and participant identifier and SAM status as random effects are indicated. Coefficients (Coef.) and p-values above each graph indicate model estimates for cases versus controls at baseline and those above each subsequent visit indicate model estimates for difference relative to baseline within the cases.

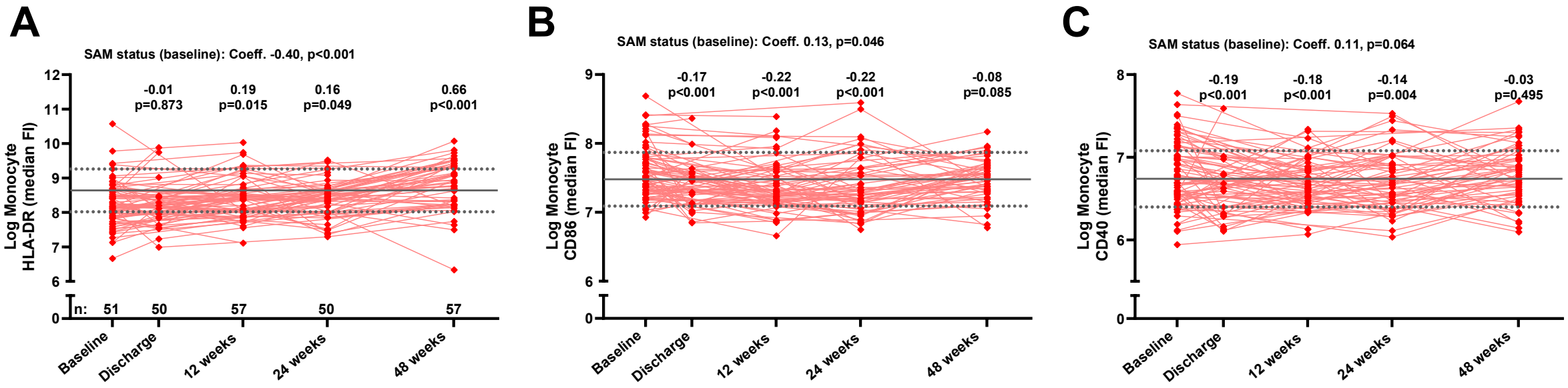

Supplementary Figure 6. Longitudinal monocyte surface activation marker expression in unstimulated blood samples cultured for 24h. Spaghetti plots of longitudinal log(n+1) transformed median FI of (A) HLA-DR, (B) CD86, and (C) CD40 on total blood monocytes (Lineage-CD66b-HLA-DR+) from unstimulated 24h whole blood culture. Red lines link longitudinal responses of the same child in the longitudinal SAM cohort (n=83). Mean and standard deviation are indicated in grey for the healthy control group measured at baseline only (n=73). Results of unadjusted mixed regression models of log(n+1) transformed proportions by baseline SAM status, visit, the interaction between SAM status and visit as fixed effects and participant identifier and SAM status as random effects are indicated. Coefficients (Coef.) and p-values above each graph indicate model estimates for cases versus controls at baseline and those above each subsequent visit indicate model estimates for difference relative to baseline within the cases.

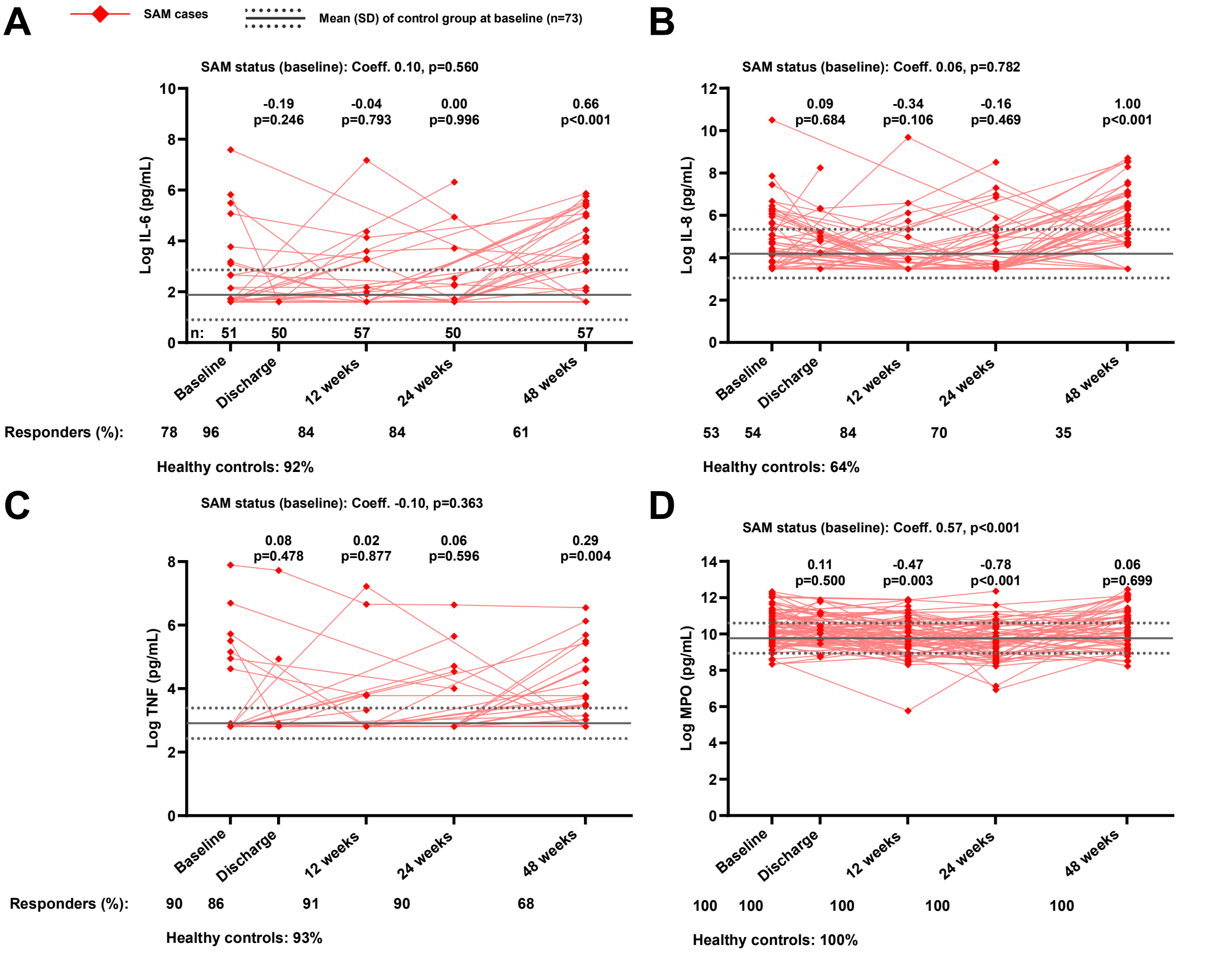

Supplementary Figure 7. Longitudinal secreted mediator concentrations in unstimulated 24h whole blood culture supernatants. Spaghetti plots of longitudinal log(n+1) transformed concentrations (pg/mL) of (A) IL-6, (B) IL-8, (C) TNF, and (D) MPO in supernatants from unstimulated 24h whole blood culture. Red lines link longitudinal responses of the same child in the longitudinal SAM cohort (n=83). Mean and standard deviation are indicated in grey for the healthy control group measured at baseline only (n=73). Results of unadjusted mixed regression models of log(n+1) transformed proportions by baseline SAM status, visit, the interaction between SAM status and visit as fixed effects and participant identifier and SAM status as random effects are indicated. Coefficients (Coef.) and p-values above each graph indicate model estimates for cases versus controls at baseline and those above each subsequent visit indicate model estimates for difference relative to baseline within the cases. Percentage of children at each visit who had a detectable response above the ELISA assay limit of detection (‘responders’) is indicated under each plot.

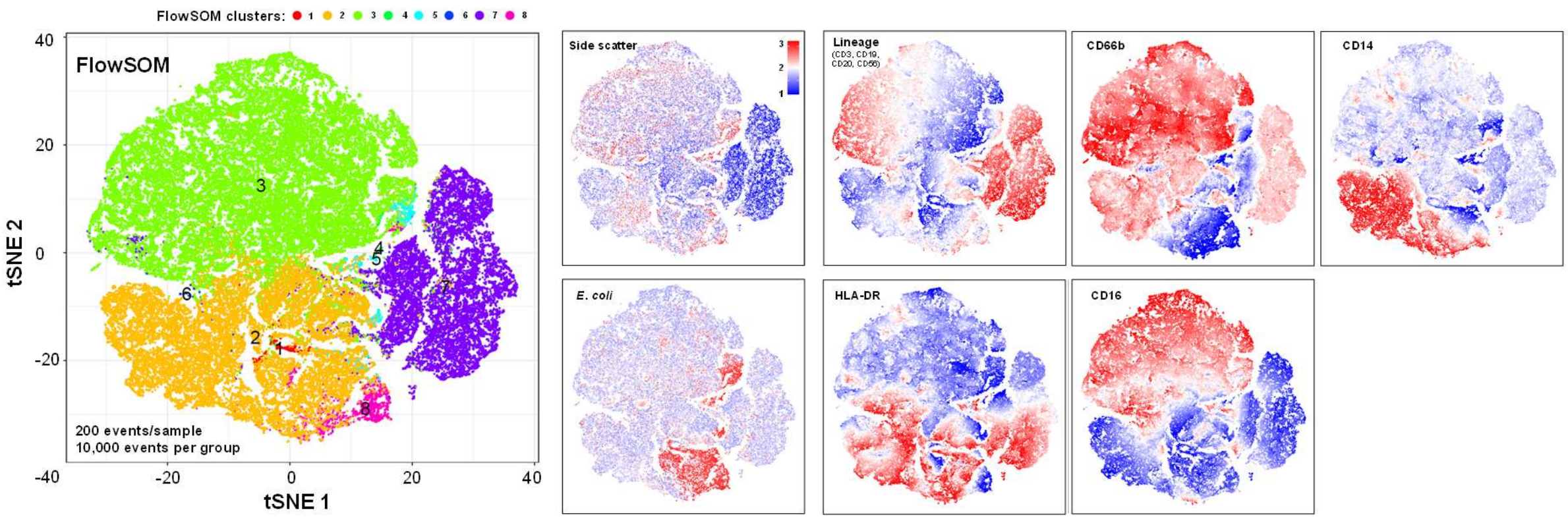

Supplementary Figure 8. FlowSOM clustering of longitudinal *E. coli*+ singlet blood leukocytes from 1h bacterial binding assays. tSNE plot of 10,000 singlet *E. coli*+ leukocyte events from each of healthy controls (n=73), SAM baseline (n=51), discharge (n=50), 12 (n=57), 24 (n=50) and 48 weeks (n=57) with equal sampling of 200 events per sample. Analyses included compensated fluorescence parameter for Lineage, CD66b, CD14, HLA-DR and CD16 cell surface marker staining. FlowSOM was applied to tSNE to identify clusters[59]; clusters are indicted by colour-coding (far left). Relative expression of each surface marker by tSNE region is indicated (red = high, blue = low) and relevant parameters excluded from tSNE and FlowSOM analyses (side-scatter and *E. coli*-AF488) are shown for reference.

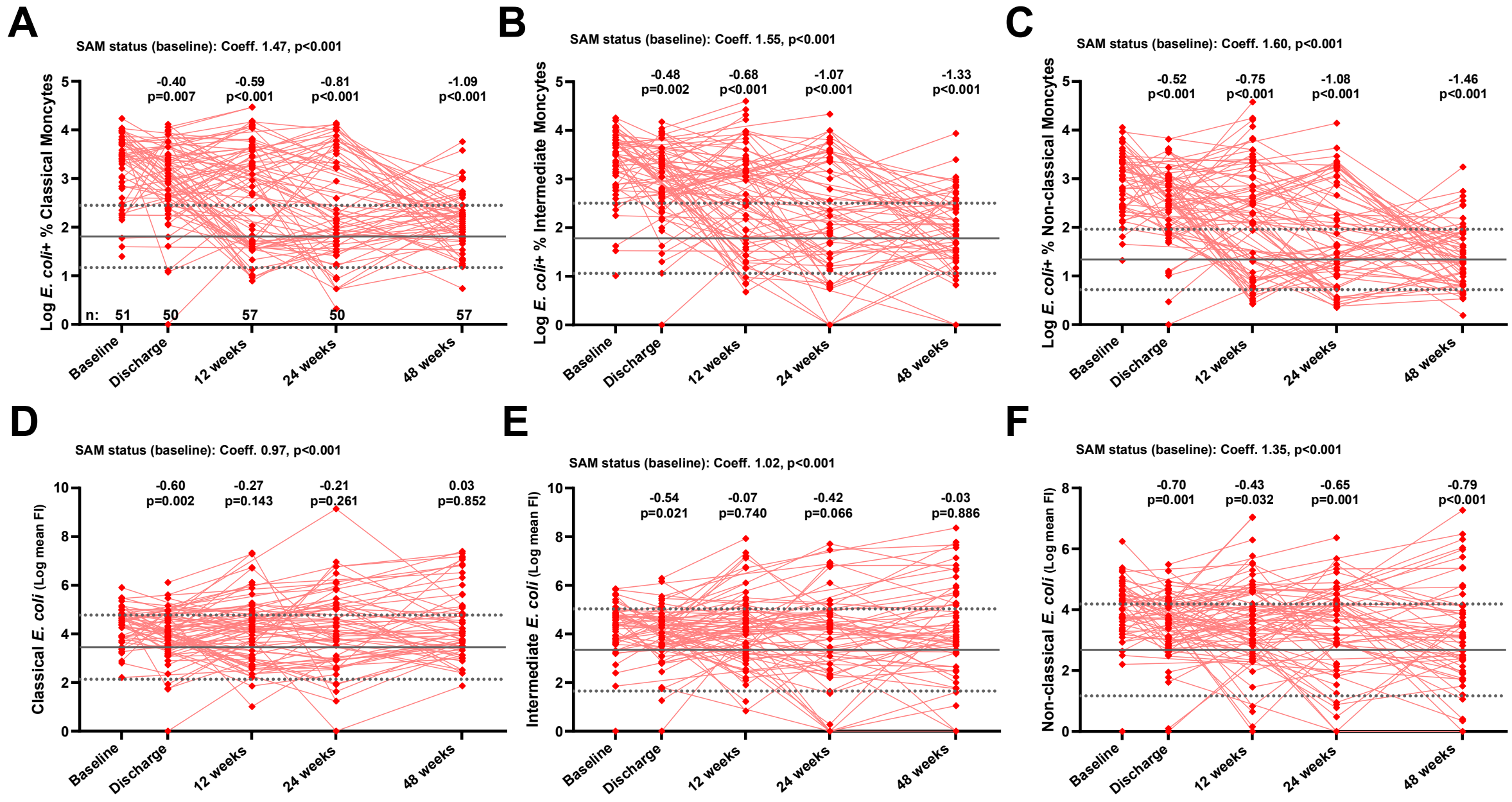

Supplementary Figure 9. Longitudinal changes in monocyte subset bacterial binding capacity. Spaghetti plots of longitudinal log(n+1) transformed proportions of *E. coli*+ cells within (A) classical (CD14+CD16-), (B) intermediate (CD14+CD16+), and (C) non classical (CD14^{lo}CD16+) monocyte subsets after 1h bacterial binding assay. Spaghetti plots of longitudinal log(n+1) transformed mean FI of *E. coli*-AF488 within (A) classical (CD14+CD16-), (B) intermediate (CD14+CD16+), and (C) non classical (CD14^{lo}CD16+) monocyte subsets after 1h bacterial binding assay. Red lines link longitudinal responses of the same child in the longitudinal SAM cohort (n=83). Mean and standard deviation are indicated in grey for the healthy control group measured at baseline only (n=73). Results of unadjusted mixed regression models for monocyte subset-specific bacterial binding assay by baseline SAM status, visit, the interaction between SAM status and visit as fixed effects and participant identifier and SAM status as random effects are indicated. Coefficients (Coef.) and p-values above each graph indicate model estimates for cases versus controls at baseline and those above each subsequent visit indicate model estimates for difference relative to baseline within the cases.

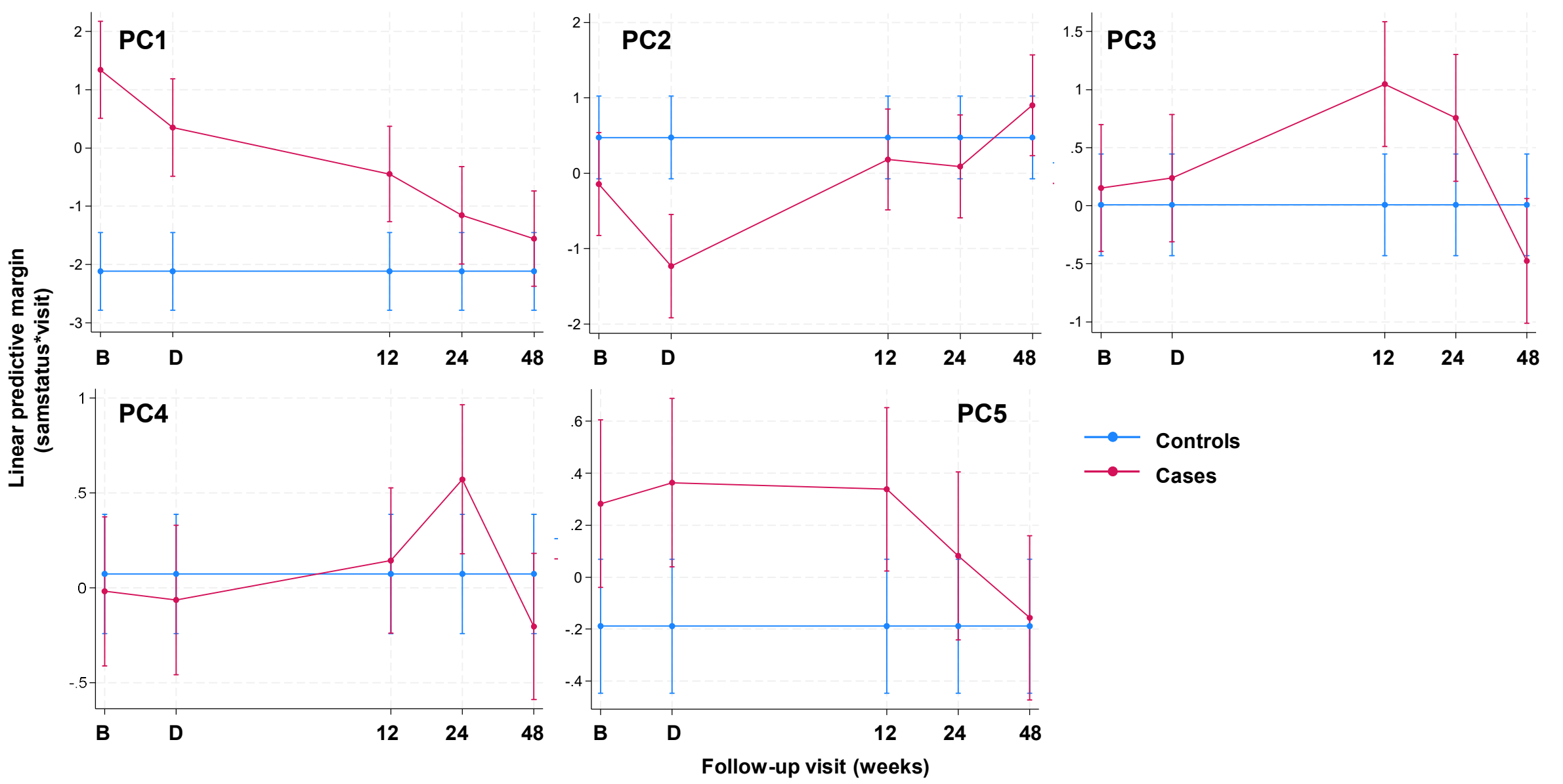

Supplementary Figure 10. Longitudinal predicted values for anti-bacterial innate immune cell function of cases and controls during follow-up from adjusted mixed models. Margins plot of linear predicted values for Principal Components (PC) 1-5 of cases (red; error bars indicate 95% Confidence interval) at baseline (B; n=51), discharge (D; n=50), 12 weeks (n=57), 24 weeks (n=50) and 48 weeks (n=57) from fitted mixed-effects linear regression models to assess the interaction between follow-up visit and case/control status, adjusted for country, sex, age group at enrolment, HIV, cerebral palsy, baseline oedema & WHZ. A random intercept was included for cases only. Controls (n=73, blue; error bars indicate 95% Confidence interval) contributed data at a single baseline visit; duplicate values for each visit for comparison. PC scores plotted in **Figure 6** and reported in **Supplementary Table 14**.

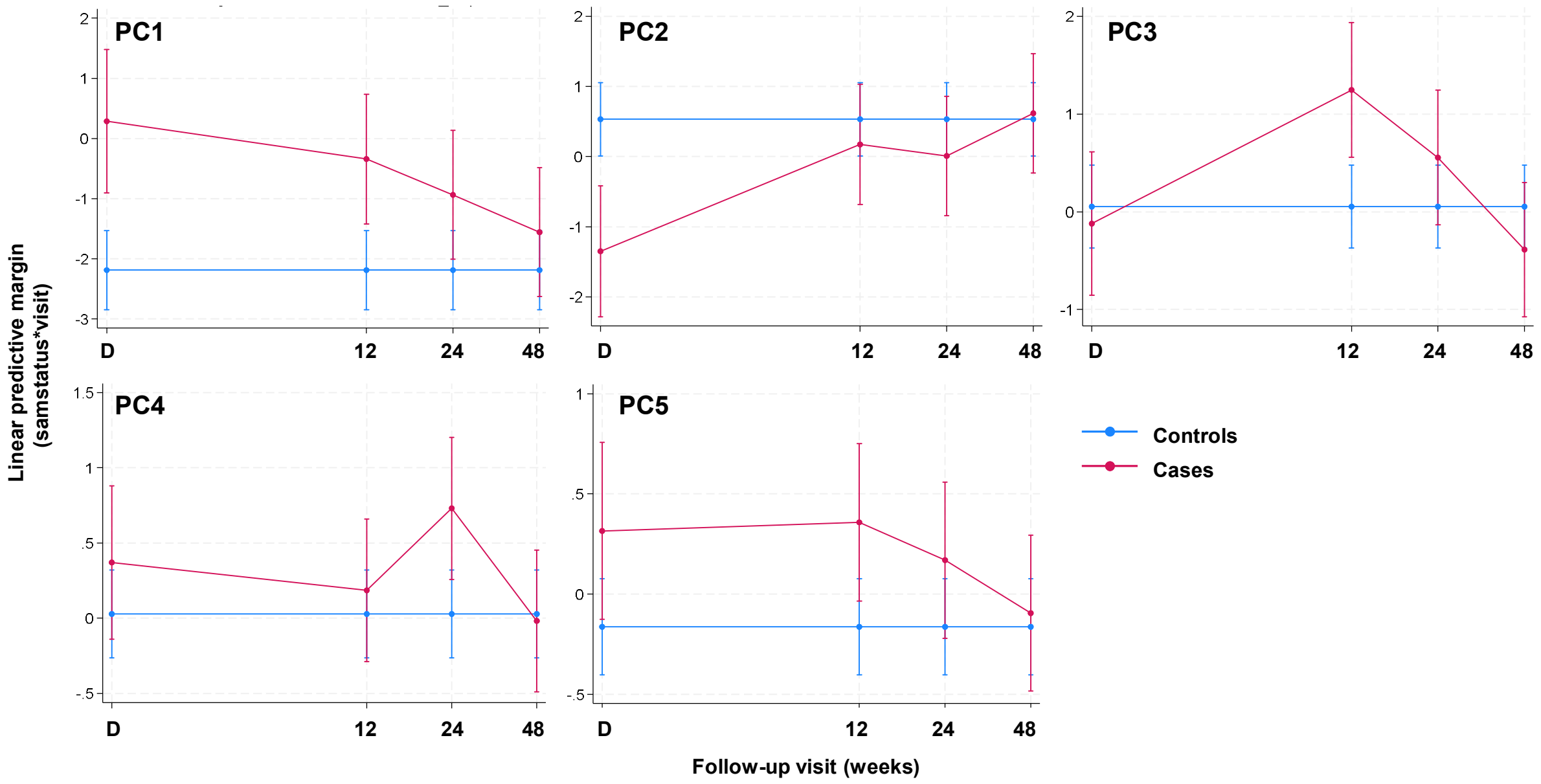

Supplementary Figure 11. Longitudinal predicted values for anti-bacterial innate immune cell function of recovered cases and controls during follow-up from adjusted mixed models. Margins plot of linear predicted values for Principal Components (PC) 1-5 of cases (red; error bars indicate 95% Confidence interval) who had recovered to a well-nourished nutritional state (WHZ> -2) at discharge (D; n=38), 12 weeks (n=47), 24 weeks (n=43) and 48 weeks (n=49) from fitted mixed-effects linear regression models to assess the interaction between follow-up visit and case/control status, adjusted for country, sex, age group at enrolment, HIV, cerebral palsy, baseline oedema & WHZ. A random intercept was included for cases only. Controls (n=73, blue; error bars indicate 95% Confidence interval) contributed data at a single baseline visit; duplicate values for each visit for comparison. PC scores plotted in **Figure 7** and model results reported in **Supplementary Table 15**.

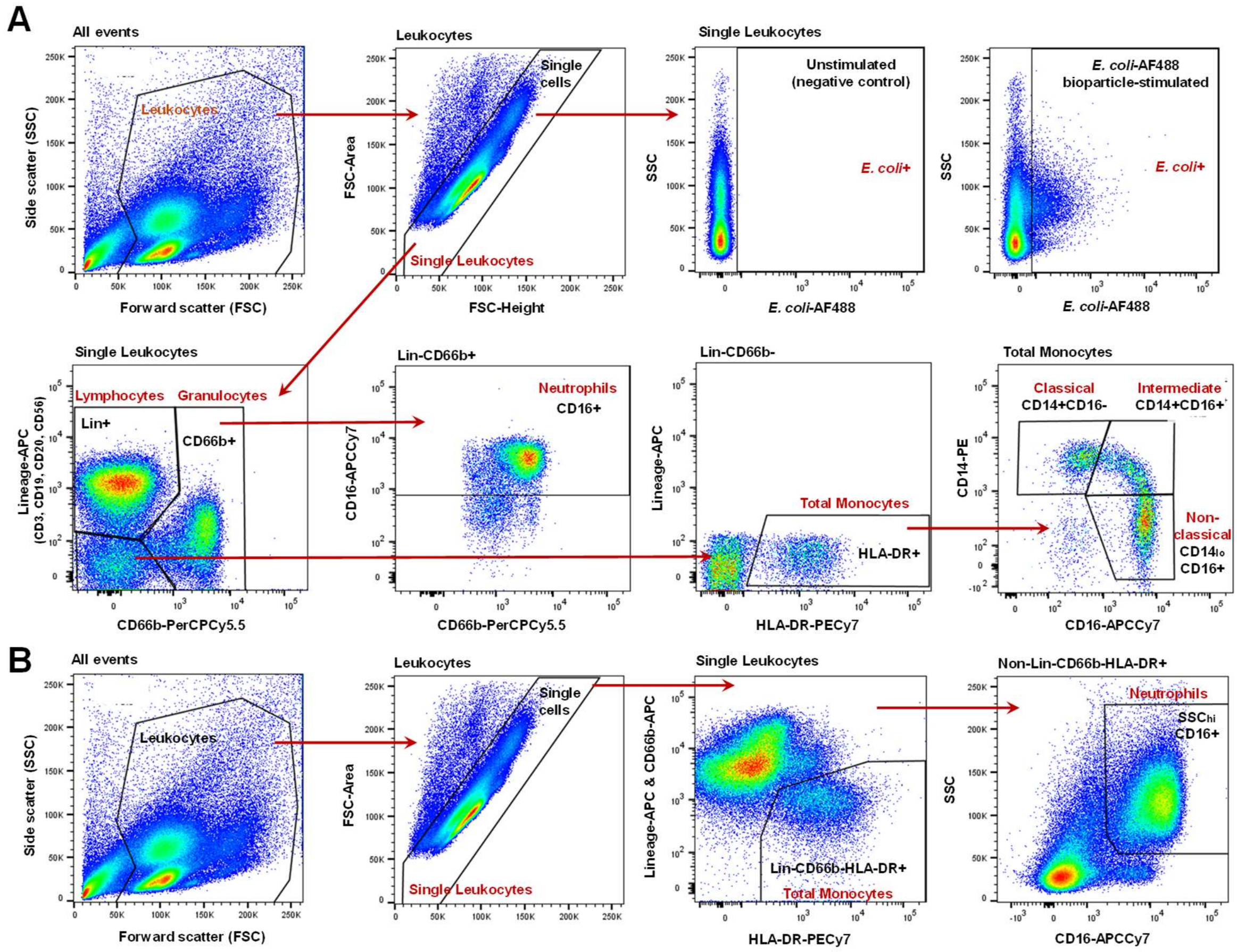

Supplementary Figure 12. Flow cytometry gating strategy for assays of innate immune cell function. A) Cell samples from 1h bacterial binding assays were first gated on side-scatter and forward-scatter to identify single leukocytes; sub-populations of single leukocytes were then identified by expression of mixed lymphocyte lineage (Lin) markers (CD3, CD19, CD20, CD56), CD66b, HLA-DR, CD14 and CD16. To identify cells that had bound and/or phagocytosed to *E. coli*-coated AF488-labelled bioparticles (*E. coli*+), single leukocytes, neutrophils, total monocytes and monocyte sub-populations were gated on AF488 fluorescence relative to paired cultures for each participant without bioparticles (negative control). The gated single leukocyte population was used for tSNE analyses of bacterial binding assays. B) Cell samples from 24h whole blood culture assays were first gated on side-scatter and forward-scatter to identify single leukocytes; sub-populations of single leukocytes were then identified by expression of Lin, CD66b, HLA-DR, granularity (side-scatter) and CD16. Activation marker (HLA-DR, CD86, CD40) expression was quantified as median fluorescence intensity within the total monocyte population (Lin-CD66b-HLA-DR+) population. For participants with samples available at multiple visits (baseline, discharge, and 12, 24, and 48 weeks), samples from each functional assay were analysed by flow cytometry on the same day and gated consistently across all timepoints to minimise batch effects.

SUPPLEMENTARY TABLES

Supplementary Table 1. Linear regression of inpatient PC scores by any caregiver-reported clinic visit outside of routine postnatal care during 48 weeks of post-discharge follow-up (n=109).

	Unadjusted			Adjusted (country, sex, age group at enrolment, HIV status, cerebral palsy, baseline oedema, MUAC & HAZ, SAM at discharge)		
	**Coef.**	**p**	**95% CI**	**Adj. Coef.**	**p**	**95% CI**
**PC1**	0.14	0.748	-0.74, 1.03	0.20	0.631	-0.62, 1.01
**PC2**	-0.31	0.464	-1.16, 0.53	-0.42	0.323	-1.27, 0.42
**PC3**	0.20	0.472	-0.35, 0.75	0.25	0.389	-0.32, 0.82
**PC4**	-0.22	0.382	-0.72, 0.28	-0.18	0.510	-0.70, 0.35
**PC5**	0.04	0.874	-0.45, 0.53	0.06	0.824	-0.44, 0.55

Supplementary Table 2. Linear regression of inpatient PC scores by adverse clinical outcome (death, readmission or SAM at 48 weeks) during 48 weeks of post-discharge follow-up (n=116).

	Unadjusted			Adjusted (country, sex, age group at enrolment, HIV status, cerebral palsy, baseline oedema, MUAC & HAZ, SAM at discharge)		
	**Coef.**	**P**	**95% CI**	**Adj. Coef.**	**p**	**95% CI**
**PC1**	0.27	0.576	-0.69, 1.24	0.57	0.248	-0.40, 1.55
**PC2**	-0.42	0.323	-1.26, 0.42	-0.18	0.726	-1.20, 0.84
**PC3**	0.00	0.995	-0.61, 0.60	-0.02	0.965	-0.84, 0.81
**PC4**	0.21	0.430	-0.32, 0.74	-0.09	0.782	-0.72, 0.54
**PC5**	0.08	0.763	-0.42, 0.58	-0.02	0.942	-0.61, 0.57

Supplementary Table 3. Linear regression of inpatient PC scores by individual adverse clinical outcomes during 48 weeks of post-discharge follow-up

	Unadjusted			Adjusted (country, sex, age group at enrolment, HIV status, cerebral palsy, baseline oedema, MUAC & HAZ, SAM at discharge)		
**Died, n=118**						
	**Coef.**	**p**	**95% CI**	**Adj. Coef.**	**p**	**95% CI**
**PC1**	0.02	0.978	-1.41, 1.45	0.29	0.709	-1.24, 1.82
**PC2**	-0.60	0.322	-1.78, 0.59	-0.17	0.867	-2.16, 1.82
**PC3**	-0.54	0.154	-1.28, 0.20	-0.64	0.280	-1.82, 0.53
**PC4**	0.35	0.455	-0.58, 1.28	0.11	0.878	-1.26, 1.48
**PC5**	0.15	0.746	-0.76, 1.05	-0.30	0.645	-1.59, 0.99
**Re-admitted to hospital, n=109**						
	**Coef.**	**p**	**95% CI**	**Adj. Coef.**	**p**	**95% CI**
**PC1**	0.18	0.749	-0.94, 1.31	0.31	0.586	-0.80, 1.41
**PC2**	0.11	0.825	-0.90, 1.13	0.07	0.893	-0.97, 1.11
**PC3**	0.13	0.766	-0.75, 1.02	0.15	0.760	-0.82, 1.11
**PC4**	0.32	0.235	-0.21, 0.86	0.24	0.442	-0.37, 0.85
**PC5**	0.20	0.439	-0.31, 0.70	0.18	0.526	-0.39, 0.76
**SAM at 48 weeks, n=85**						
	**Coef.**	**p**	**95% CI**	**Adj. Coef.**	**p**	**95% CI**
**PC1**	1.02	0.382	-1.29, 3.33	0.31	0.839	-2.74, 3.36
**PC2**	-1.41	0.044	-2.79, -0.04	-0.86	0.313	-2.55, 0.83
**PC3**	0.76	0.186	-0.37, 1.88	0.13	0.874	-1.56, 1.83
**PC4**	-0.04	0.925	-0.99, 0.90	-1.03	0.172	-2.51, 0.46
**PC5**	-0.39	0.379	-1.28, 0.49	-0.60	0.242	-1.61, 0.41

Supplementary Table 4. Linear regression of inpatient PC scores by ΔMUAC between baseline and 48 week post-discharge visit (n=85).

	Unadjusted			Adjusted (country, sex, age group at enrolment, HIV status, cerebral palsy, baseline oedema, MUAC & HAZ, SAM at discharge)		
	**Coef.**	**p**	**95% CI**	**Adj. Coef.**	**p**	**95% CI**
**PC1**	-1.38	0.093	-2.98, 0.23	-0.45	0.508	-1.78, 0.89
**PC2**	1.16	0.192	-0.59, 2.90	1.14	0.044	0.03, 2.25
**PC3**	0.67	0.584	-1.76, 3.11	0.48	0.682	-1.83, 2.79
**PC4**	1.86	0.175	-0.84, 4.57	1.08	0.282	-0.90, 3.06
**PC5**	-0.10	0.963	-4.27, 4.07	-1.42	0.266	-3.93, 1.10

Supplementary Table 5. Linear regression of individual variables loaded onto PC2 by ΔMUAC between baseline and 48 week post-discharge visit (n=85).

	Unadjusted			Adjusted (country, sex, age group at enrolment, HIV status, cerebral palsy, baseline oedema, MUAC & HAZ, SAM at discharge)		
	**Coef.**	**p**	**95% CI**	**Adj. Coef.**	**p**	**95% CI**
**LPS-specific[1] monocyte CD40**	0.16	0.841	-1.38, 1.70	0.06	0.914	-1.03, 1.15
**LPS-specific IL-6**	0.14	0.836	-1.24, 1.53	0.36	0.469	-0.63, 1.34
**HKST-specific[2] IL-6**	0.46	0.742	-2.29, 3.20	1.59	0.225	-0.99, 4.17
**LPS-specific IL-8**	1.09	0.085	-0.15, 2.33	0.57	0.203	-0.31, 1.45
**LPS-specific TNF**	1.65	0.096	-0.30, 3.61	0.84	0.144	-0.29, 1.98
**HKST-specific TNF**	2.65	0.102	-0.54, 5.83	3.21	0.012	0.72, 5.71

[1]Difference (Δ) between median FI (CD40) or pg/mL (TNF, IL-6, IL-8) in LPS-stimulated cultures and unstimulated cultures. Censored at 0 and log(n+1) transformed for regression analysis.

[2]Difference (Δ) between median FI (CD40) or pg/mL (TNF, IL-6, IL-8) in HKST-stimulated cultures and unstimulated cultures. Censored at 0 and log(n+1) transformed for regression analysis.

Supplementary Table 6. Linear regression of inpatient PC scores by ΔWHZ between baseline and 48 week post-discharge visit (n=85).

	Unadjusted			Adjusted (country, sex, age group at enrolment, HIV status, cerebral palsy, baseline oedema, WHZ & HAZ, SAM at discharge)		
	**Coef.**	**p**	**95% CI**	**Adj. Coef.**	**p**	**95% CI**
**PC1**	0.03	0.712	-0.14, 0.21	0.02	0.730	-0.10, 0.14
**PC2**	0.23	0.003	0.08, 0.38	0.10	0.065	-0.01, 0.21
**PC3**	0.09	0.460	-0.16, 0.35	0.02	0.816	-0.16, 0.20
**PC4**	0.16	0.167	-0.07, 0.38	0.12	0.156	-0.05, 0.28
**PC5**	0.04	0.837	-0.33, 0.41	-0.04	0.754	-0.28, 0.20

Supplementary Table 7. Linear regression of individual variables loaded onto PC2 by ΔWHZ between baseline and 48 week post-discharge visit (n=85).

	Unadjusted			Adjusted (country, sex, age group at enrolment, HIV status, cerebral palsy, baseline oedema, WHZ & HAZ, SAM at discharge)		
	**Coef.**	**p**	**95% CI**	**Adj. Coef.**	**p**	**95% CI**
**LPS-specific[1] Monocyte CD40**	0.06	0.396	-0.08, 0.21	-0.01	0.850	-0.12, 0.10
**LPS-specific IL-6**	0.10	0.102	-0.02, 0.22	0.03	0.574	-0.06, 0.12
**HKST-specific[2] IL-6**	0.21	0.010	0.05, 0.37	0.13	0.066	-0.01, 0.27
**LPS-specific IL-8**	0.08	0.126	-0.02, 0.19	0.07	0.030	0.01, 0.13
**LPS-specific TNF**	0.11	0.154	-0.04, 0.27	0.00	0.368	-0.06, 0.17
**HKST-specific TNF**	0.11	0.314	-0.11, 0.34	0.14	0.117	-0.03, 0.3

[1]Difference (Δ) between median FI (CD40) or pg/mL (TNF, IL-6, IL-8) in LPS-stimulated cultures and unstimulated cultures. Censored at 0 and log(n+1) transformed for regression analysis.

[2]Difference (Δ) between median FI (CD40) or pg/mL (TNF, IL-6, IL-8) in HKST-stimulated cultures and unstimulated cultures. Censored at 0 and log(n+1) transformed for regression analysis.

Supplementary Table 8. Mixed linear regression of longitudinal proportions of blood leukocyte sub-sets in unstimulated 1h blood cultures by SAM group and visit as fixed effects and participant identifier and SAM group as random effects (SAM group n=83, healthy controls n=73).

	SAM status (baseline)				SAM status*Visit			
		Coef.	p	95% CI		Coef.	p	95% CI
**Total leukocyte count**	**Healthy Controls**	*Reference*			**Baseline**	*Reference*		
	**SAM**	22779	0.068	-1665, 47224	**Discharge**	-23758	0.037	-46030, -1487
					**12 weeks**	-37079	0.001	-58402, -15755
					**24 weeks**	-29142	0.009	-50932, -7353
					**48 weeks**	-38530	<0.001	-59626, -17435
**Total lymphocyte proportions[1]**	**Healthy Controls**	*Reference*			**Baseline**	*Reference*		
	**SAM**	-0.02	0.156	-0.05, 0.01	**Discharge**	0.02	0.136	-0.01, 0.05
					**12 weeks**	0.02	0.159	-0.01, 0.04
					**24 weeks**	0.02	0.070	0.00, 0.05
					**48 weeks**	0.02	0.229	-0.01, 0.04
**Neutrophil proportions[1]**	**Healthy Controls**	*Reference*			**Baseline**	*Reference*		
	**SAM**	0.01	0.461	-0.02, 0.04	**Discharge**	-0.01	0.468	-0.04, 0.02
					**12 weeks**	-0.02	0.253	-0.05, 0.01
					**24 weeks**	-0.03	0.087	-0.05, 0.00
					**48 weeks**	-0.01	0.574	-0.04, 0.02
**Total monocyte proportions[1]**	**Healthy Controls**	*Reference*			**Baseline**	*Reference*		
	**SAM**	-0.01	0.030	-0.02, 0.00	**Discharge**	0.00	0.462	-0.01, 0.00
					**12 weeks**	0.00	0.850	-0.01, 0.01
					**24 weeks**	0.00	0.909	-0.01, 0.01
					**48 weeks**	0.00	0.647	0.00, 0.01
**Classical monocyte proportions[2]**	**Healthy Controls**	*Reference*			**Baseline**	*Reference*		
	**SAM**	-0.02	0.205	-0.06, 0.01	**Discharge**	-0.01	0.627	-0.04, 0.02
					**12 weeks**	0.00	0.990	-0.03, 0.03

					**24 weeks**	0.01	0.435	-0.02, 0.04
					**48 weeks**	0.03	0.016	0.01, 0,06
**Intermediate monocyte proportions[2]**	**Healthy Controls**	*Reference*			**Baseline**	*Reference*		
	**SAM**	0.05	<0.001	0.03, 0.07	**Discharge**	-0.03	0.001	-0.04, -0.01
					**12 weeks**	-0.02	0.010	-0.04, 0.00
					**24 weeks**	-0.03	<0.001	-0.05, -0.02
					**48 weeks**	-0.04	<0.001	-0.06, -0.03
**Non-classical monocyte proportions[2]**	**Healthy Controls**	*Reference*			**Baseline**	*Reference*		
	**SAM**	-0.04	0.040	-0.07, 0.00	**Discharge**	0.02	0.228	-0.01, 0.05
					**12 weeks**	0.06	<0.001	0.04, 0.09
					**24 weeks**	0.03	0.023	0.00, 0.06
					**48 weeks**	0.04	0.007	0.01, 0.07

[1]Proportion of total singlet leukocytes

[2]Proportion of total monocytes (Lin-CD66b-HLA-DR+)

All soluble mediators, censored at ELISA limit of detection. All variables log(n+1) transformed

Supplementary Table 9. Mixed linear regression of longitudinal monocyte surface marker expression and whole blood soluble mediator concentrations in unstimulated 24h blood cultures by SAM group and visit as fixed effects and participant identifier and SAM group as random effects (SAM group n=83, healthy controls n=73).

All soluble mediators, censored at ELISA limit of detection. All variables log(n+1) transformed

		SAM status (baseline)				SAM status*Visit		
		**Coef.**	**p**	**95% CI**		**Coef.**	**p**	**95% CI**
**Monocyte HLA-DR (median FI)**	**Healthy Controls**	*Reference*			**Baseline**	*Reference*		
	**SAM**	-0.40	<0.001	-0.61, -0.20	**Discharge**	-0.01	0.873	-0.18, 0.15
					**12 weeks**	0.19	0.015	0.04, 0.35
					**24 weeks**	0.16	0.049	0.00, 0.32
					**48 weeks**	0.66	<0.001	0.51, 0.82
**Monocyte CD86 (median FI)**	**Healthy Controls**	*Reference*			**Baseline**	*Reference*		
	**SAM**	0.13	0.046	0.00, 0.25	**Discharge**	-0.17	<0.001	-0.27, -0.08
					**12 weeks**	-0.22	<0.001	-0.31, -0.13
					**24 weeks**	-0.22	<0.001	-0.32, -0.13
					**48 weeks**	-0.08	0.085	-0.17, 0.01
**Monocyte CD40 (median FI)**	**Healthy Controls**	*Reference*			**Baseline**	*Reference*		
	**SAM**	0.11	0.064	-0.01, 0.22	**Discharge**	-0.19	<0.001	-0.29, -0.09
					**12 weeks**	-0.18	<0.001	-0.27, -0.08
					**24 weeks**	-0.14	0.004	-0.24, -0.04
					**48 weeks**	-0.03	0.495	-0.13, 0.06
**TNF (pg/mL)**	**Healthy Controls**	*Reference*			**Baseline**	*Reference*		
	**SAM**	0.10	0.363	-0.12, 0.32	**Discharge**	0.08	0.478	-0.14, 0.29
					**12 weeks**	0.02	0.877	-0.19, 0.22
					**24 weeks**	0.06	0.596	-0.15, 0.27
					**48 weeks**	0.29	0.004	0.09, 0.50
**IL-6 (pg/mL)**	**Healthy Controls**	*Reference*			**Baseline**	*Reference*		
	**SAM**	0.10	0.560	-0.23, 0.43	**Discharge**	-0.19	0.246	-0.51, 0.13
					**12 weeks**	-0.04	0.793	-0.35, 0.27
					**24 weeks**	0.00	0.996	-0.31, 0.31
					**48 weeks**	0.66	<0.001	0.36, 0.96
**IL-8 (pg/mL)**	**Healthy Controls**	*Reference*			**Baseline**	*Reference*		
	**SAM**	0.06	0.782	-0.35, 0.47	**Discharge**	0.09	0.684	-0.34, 0.52
					**12 weeks**	-0.34	0.106	-0.75, 0.07
					**24 weeks**	-0.16	0.469	-0.58, 0.27

					**48 weeks**	1.00	<0.001	0.59, 1.41
**MPO (pg/mL)**	**Healthy Controls**	*Reference*			**Baseline**	*Reference*		
	**SAM**	0.57	<0.001	0.26, 0.88	**Discharge**	0.11	0.500	-0.43, 0.21
					**12 weeks**	-0.47	0.003	-0.78, -0.16
					**24 weeks**	-0.78	<0.001	-1.10, -0.47
					**48 weeks**	0.06	0.699	-0.25, 0.37

Supplementary Table 10. Mixed linear regression of longitudinal proportions of *E. coli*+ immune cells after 1h bacterial binding assay by SAM group and visit as fixed effects and participant identifier and SAM group as random effects (SAM group n=83, healthy controls n=73).

All variables censored at 0 and log(n+1) transformed

	SAM status (baseline)				SAM status*Visit			
		**Coef.**	**p**	**95% CI**		**Coef.**	**p**	**95% CI**
***E. coli*+ % Total leukocytes**	**Healthy Controls**	*Reference*			**Baseline**	*Reference*		
	**SAM**	1.03	<0.001	0.85, 1.22	**Discharge**	-0.30	0.003	-0.50, -0.10
					**12 weeks**	-0.53	<0.001	-0.72, -0.34
					**24 weeks**	-0.69	<0.001	-0.88, -0.49
					**48 weeks**	-0.88	<0.001	-1.07, -0.69
***E. coli*+ % Total lymphocytes**	**Healthy Controls**	*Reference*			**Baseline**	*Reference*		
	**SAM**	0.58	<0.001	0.44, 0.72	**Discharge**	-0.14	0.054	-0.28, 0.00
					**12 weeks**	-0.39	<0.001	-0.52, -0.26
					**24 weeks**	-0.43	<0.001	-0.57, 0.29
					**48 weeks**	-0.46	<0.001	-0.59, -0.32
***E. coli*+ % Neutrophils**	**Healthy Controls**	*Reference*			**Baseline**	*Reference*		
	**SAM**	1.46	<0.001	1.23, 1.69	**Discharge**	-0.37	0.004	-0.62, -0.12
					**12 weeks**	-0.59	<0.001	-0.83, -0.35
					**24 weeks**	-0.97	<0.001	-1.21, -0.72
					**48 weeks**	-1.34	<0.001	-1.58, -1.10
***E. coli*+ % Total monocytes**	**Healthy Controls**	*Reference*			**Baseline**	*Reference*		
	**SAM**	1.53	<0.001	1.29, 1.77	**Discharge**	-0.35	0.009	-0.62, -0.09
					**12 weeks**	-0.69	<0.001	-0.94, -0.43
					**24 weeks**	-0.96	<0.001	-1.22, -0.70
					**48 weeks**	-1.16	<0.001	-1.42, -0.91
***E. coli*+ % Classical monocytes**	**Healthy Controls**	*Reference*			**Baseline**	*Reference*		
	**SAM**	1.47	<0.001	1.21, 1.73	**Discharge**	-0.40	0.007	-0.69, -0.11
					**12 weeks**	-0.59	<0.001	-0.87, -0.31
					**24 weeks**	-0.81	<0.001	-1.10, -0.53
					**48 weeks**	-1.09	<0.001	-1.36, -0.81
***E. coli*+ % Intermediate monocytes**	**Healthy Controls**	*Reference*			**Baseline**	*Reference*		
	**SAM**	1.55	<0.001	1.27, 1.83	**Discharge**	-0.48	0.002	-0.77, -0.18
					**12 weeks**	-0.68	<0.001	-0.96, -0.40
					**24 weeks**	-1.07	<0.001	-1.36, -0.78

					**48 weeks**	-1.33	<0.001	-1.61, -1.05
***E. coli*+ % Non-classical monocytes**	**Healthy Controls**	*Reference*			**Baseline**	*Reference*		
	**SAM**	1.60	<0.001	1.34, 1.86	**Discharge**	-0.52	<0.001	-0.81, -0.23
					**12 weeks**	-0.75	<0.001	-1.03, -0.47
					**24 weeks**	-1.08	<0.001	-1.36, -0.79
					**48 weeks**	-1.46	<0.001	-1.74, -1.18

Supplementary Table 11. Mixed linear regression of longitudinal *E. coli* mean FI by immune cell subset after 1h bacterial binding assay by SAM group and visit as fixed effects and participant identifier and SAM group as random effects (cases n=83, controls n=73).

All variables censored at 0 and log(n+1) transformed

	SAM status (baseline)				SAM status*Visit			
		Coef.	p	95% CI		Coef.	p	95% CI
**Total leukocytes (mean FI)**	**Healthy Controls**	*Reference*			**Baseline**	*Reference*		
	**SAM**	-1.18	<0.001	-1.38, -0.97	**Discharge**	-0.29	0.001	-0.47, -0.11
					**12 weeks**	0.43	<0.001	-0.25, -0.60
					**24 weeks**	0.93	<0.001	0.75, 1.10
					**48 weeks**	1.49	<0.001	1.32, 1.66
**Total lymphocytes (mean FI)**	**Healthy Controls**	*Reference*			**Baseline**	*Reference*		
	**SAM**	-1.04	<0.001	-1.40, -0.67	**Discharge**	-0.16	0.304	-0.47, 0.15
					**12 weeks**	0.27	0.077	-0.03, 0.56
					**24 weeks**	0.70	<0.001	0.39, 1.00
					**48 weeks**	1.29	<0.001	1.00, 1.59
**Neutrophils (mean FI)**	**Healthy Controls**	*Reference*			**Baseline**	*Reference*		
	**SAM**	0.64	<0.001	0.30, 0.98	**Discharge**	-0.40	0.007	-0.68, -0.11
					**12 weeks**	-0.24	0.088	-0.52, 0.04
					**24 weeks**	-0.63	<0.001	-0.91, -0.35
					**48 weeks**	-0.42	0.003	-0.69, -0.15
**Total monocytes (mean FI)**	**Healthy Controls**	*Reference*			**Baseline**	*Reference*		
	**SAM**	0.61	0.009	0.15, 1.08	**Discharge**	-0.44	0.024	-0.83, -0.06
					**12 weeks**	-0.22	0.242	-0.59, 0.15
					**24 weeks**	-0.19	0.324	-0.56, 0.19
					**48 weeks**	0.28	0.125	-0.08, 0.65
**Classical monocytes (mean FI)**	**Healthy Controls**	*Reference*			**Baseline**	*Reference*		
	**SAM**	0.97	<0.001	0.54, 1.39	**Discharge**	-0.60	0.002	-0.98, -0.22
					**12 weeks**	-0.27	0.143	-0.63, 0.09
					**24 weeks**	-0.21	0.261	-0.58, 0.16
					**48 weeks**	0.03	0.852	-0.32, 0.39
**Intermediate monocytes (mean FI)**	**Healthy Controls**	*Reference*			**Baseline**	*Reference*		
	**SAM**	1.02	<0.001	0.49, 1.55	**Discharge**	-0.54	0.021	-1.00, -0.08
					**12 weeks**	-0.07	0.740	-0.51, 0.37
					**24 weeks**	-0.42	0.066	-0.87, 0.03

					**48 weeks**	-0.03	0.886	-0.47, 0.40
**Non-classical monocytes (mean FI)**	**Healthy Controls**	*Reference*			**Baseline**	*Reference*		
	**SAM**	1.35	<0.001	0.88, 1.83	**Discharge**	-0.70	0.001	-1.11, -0.29
					**12 weeks**	-0.43	0.032	-0.82, -0.04
					**24 weeks**	-0.65	0.001	-1.05, -0.25
					**48 weeks**	-0.79	<0.001	-1.18, -0.40

Supplementary Table 12. Mixed linear regression of longitudinal monocyte surface marker expression and whole blood soluble mediator concentrations in LPS-stimulated 24h blood cultures by SAM group and visit as fixed effects and participant identifier and SAM group as random effects (cases n=83, controls n=73).

All soluble mediators, censored at ELISA limit of detection. All variables log(n+1) transformed.

[1]Difference (Δ) between median FI (HLA-DR, CD86, CD40) or pg/mL (TNF, IL-6, IL-8, MPO) in LPS-stimulated cultures and unstimulated cultures

	SAM status (baseline)				SAM status*Visit			
		Coef.	p	95% CI		Coef.	p	95% CI
**LPS-specific monocyte[1] HLA-DR**	**Healthy Controls**	*Reference*			**Baseline**	*Reference*		
	**SAM**	-1.07	<0.001	-1.65, -0.49	**Discharge**	-0.43	0.212	-1.09, 0.24
					**12 weeks**	1.17	<0.001	0.53, 1.81
					**24 weeks**	1.15	0.001	0.49, 1.80
					**48 weeks**	0.24	0.462	-0.40, 0.88
**LPS-specific monocyte CD86**	**Healthy Controls**	*Reference*			**Baseline**	*Reference*		
	**SAM**	-0.98	0.018	-1.79, -0.16	**Discharge**	0.34	0.385	-0.43, 1.12
					**12 weeks**	2.00	<0.001	1.26, 2.74
					**24 weeks**	1.42	<0.001	0.66, 2.18
					**48 weeks**	0.44	0.239	-0.29, 1.17
**LPS-specific monocyte CD40**	**Healthy Controls**	*Reference*			**Baseline**	*Reference*		
	**SAM**	-1.15	<0.001	-1.72, -0.59	**Discharge**	-0.15	0.620	-0.74, 0.44
					**12 weeks**	1.11	<0.001	0.54, 1.68
					**24 weeks**	1.05	<0.001	0.46, 1.63
					**48 weeks**	1.14	<0.001	0.57, 1.70
**LPS-specific TNF**	**Healthy Controls**	*Reference*			**Baseline**	*Reference*		
	**SAM**	-0.97	0.004	-1.62, -0.31	**Discharge**	-0.63	0.048	-1.25, -0.01
					**12 weeks**	0.24	0.421	-0.35, 0.84
					**24 weeks**	-0.01	0.984	-0.61, 0.60
					**48 weeks**	1.69	<0.001	1.11, 2.28
**LPS-specific IL-6**	**Healthy Controls**	*Reference*			**Baseline**	*Reference*		
	**SAM**	-1.06	0.018	-1.94, -0.18	**Discharge**	-0.60	0.122	-1.37, 0.16
					**12 weeks**	0.65	0.082	-0.08, 1.38
					**24 weeks**	0.58	0.127	-0.17, 1.33
					**48 weeks**	2.42	<0.001	1.70, 3.15
**LPS-specific IL-8**	**Healthy Controls**	*Reference*			**Baseline**	*Reference*		
	**SAM**	-1.34	0.001	-2.15, -0.53	**Discharge**	-0.50	0.198	-1.25, 0.26

					**12 weeks**	0.40	0.284	-0.33, 1.12
					**24 weeks**	0.57	0.128	-0.17, 1.31
					**48 weeks**	2.60	<0.001	1.88, 3.31
**LPS-specific MPO**	**Healthy Controls**	*Reference*			**Baseline**	*Reference*		
	**SAM**	-1.42	0.021	-2.63, -0.22	**Discharge**	-0.07	0.909	-1.29, 1.15
					**12 weeks**	1.58	0.008	0.41, 2.75
					**24 weeks**	2.89	<0.001	1.70, 4.09
					**48 weeks**	0.55	0.349	-0.60, 1.71

Supplementary Table 13. Mixed linear regression of longitudinal monocyte surface marker expression and whole blood soluble mediator concentrations in HKST-stimulated 24h blood cultures by SAM group and visit as fixed effects and participant identifier and SAM group as random effects (cases n=83, controls n=73).

All soluble mediators, censored at ELISA limit of detection. All variables log(n+1) transformed

[1]Difference (Δ) between median FI (HLA-DR, CD86, CD40) or pg/mL (TNF, IL-6, IL-8, MPO) in HKST-stimulated cultures and unstimulated cultures

		SAM status (baseline)				SAM status*Visit		
		Coef.	p	95% CI		Coef.	p	95% CI
**HKST-specific[1] monocyte HLA-DR**	**Healthy Controls**	*Reference*			**Baseline**	*Reference*		
	**SAM**	-0.01	0.989	-0.85, 0.84	**Discharge**	-0.81	0.051	-1.62, 0.00
					**12 weeks**	0.84	0.034	0.06, 1.61
					**24 weeks**	0.46	0.252	-0.33, 1.26
					**48 weeks**	-1.37	<0.001	-2.14, -0.61
**HKST-specific monocyte CD86**	**Healthy Controls**	*Reference*			**Baseline**	*Reference*		
	**SAM**	-1.26	0.006	-2.16, -0.36	**Discharge**	1.27	0.003	0.44, 2.09
					**12 weeks**	1.98	<0.001	1.19, 2.77
					**24 weeks**	1.76	<0.001	0.95, 2.57
					**48 weeks**	0.57	0.257	-0.22, 1.35
**HKST-specific monocyte CD40**	**Healthy Controls**	*Reference*			**Baseline**	*Reference*		
	**SAM**	-1.19	<0.001	-1.59, -0.79	**Discharge**	-0.17	0.479	-0.65, 0.31
					**12 weeks**	0.77	0.001	0.31, 1.24
					**24 weeks**	0.85	<0.001	0.38, 1.32
					**48 weeks**	0.93	<0.001	0.47, 1.39
**HKST-specific TNF**	**Healthy Controls**	*Reference*			**Baseline**	*Reference*		
	**SAM**	-1.09	<0.001	-1.46, -0.72	**Discharge**	-0.40	0.033	-0.77, -0.03
					**12 weeks**	0.44	0.016	0.08, 0.79
					**24 weeks**	0.60	0.001	0.24, 0.96
					**48 weeks**	1.20	<0.001	0.84, 1.55
**HKST-specific IL-6**	**Healthy Controls**	*Reference*			**Baseline**	*Reference*		
	**SAM**	-0.57	0.005	-0.98, -0.17	**Discharge**	-0.38	0.028	-0.72, -0.04
					**12 weeks**	0.09	0.600	-0.24, 0.41
					**24 weeks**	0.36	0.033	0.03, 0.69
					**48 weeks**	0.83	<0.001	0.51, 1.15
**HKST-specific IL-8**	**Healthy Controls**	*Reference*			**Baseline**	*Reference*		
	**SAM**	-1.56	<0.001	-2.04, -1.08	**Discharge**	-0.19	0.443	-0.66, 0.29
					**12 weeks**	0.43	0.063	-0.02, 0.88
					**24 weeks**	0.95	<0.001	0.49, 1.41

					**48 weeks**	1.68	<0.001	1.23, 2.13
**HKST-specific MPO**	**Healthy Controls**	*Reference*			**Baseline**	*Reference*		
	**SAM**	-1.36	0.005	-2.30, -0.42	**Discharge**	-0.09	0.871	-1.12, 0.95
					**12 weeks**	0.93	0.065	-0.06, 1.93
					**24 weeks**	1.65	0.001	0.64, 2.66
					**48 weeks**	0.18	0.727	-0.81, 1.16

Supplementary Table 14. Mixed linear regression of anti-bacterial innate immune cell function principal component (PC) scores by SAM group and visit as fixed effects and participant identifier and SAM group as random effects (cases n=83, controls n=73).

All variables censored at 0 and log(n+1) transformed.

Bas. – baseline, Dis. – discharge, 12wk – 12 week post-discharge visit, 24wk – 24 week post-discharge visit, 48wk – 48 week post-discharge visit

		**Unadjusted**							**Adjusted (country, sex, age group at enrolment, HIV status, cerebral palsy, baseline oedema, baseline WHZ)**							
		**SAM status (baseline)**			**SAM status*Visit**				**SAM status (baseline)**				**SAM status*Visit**			
		**Coef.**	**p**	**95% CI**		**Coef.**	**p**	**95% CI**		**Adj. Coef.**	**p**	**95% CI**		**Adj. Coef.**	**p**	**95% CI**
**PC1**	**Healthy**	*Reference*			**Bas.**	*Reference*			**Healthy**	*Reference*			**Bas.**	*Reference*		
	**SAM**	4.05	<0.001	3.30, 4.81	**Dis.**	-0.97	0.010	-1.72, -0.23	**SAM**	3.46	<0.001	2.14, 4.77	**Dis.**	-0.99	0.009	-1.73, -0.25
					**12w**	-1.78	<0.001	-2.50, -1.07					**12w**	-1.79	<0.001	-2,50, -1.07
					**24w**	-2.53	<0.001	-3.26, -1.80					**24w**	-2.50	<0.001	-3.22, -1.77
					**48w**	-2.94	<0.001	-3.64, -2.23					**48w**	-2.90	<0.001	-3.60, -2.19
**PC2**	**Healthy**	*Reference*			**Bas.**	*Reference*			**Healthy**	*Reference*			**Bas.**	*Reference*		
	**SAM**	-0.15	0.629	-0.76, 0.46	**Dis.**	-1.09	<0.001	-1.68, -0.49	**SAM**	-0.62	0.261	-1.70, 0.46	**Dis.**	-1.09	<0.001	-1.68, -0.50
					**12w**	0.32	0.272	-0.25, 0.89					**12w**	0.33	0.260	-0.24, 0.89
					**24w**	0.24	0.416	-0.34, 0.82					**24w**	0.23	0.430	-0.35, 0.81
					**48w**	1.01	<0.001	0.45, 1.58					**48w**	1.04	<0.001	0.48, 1.61
**PC3**	**Healthy**	*Reference*			**Bas.**	*Reference*			**Healthy**	*Reference*			**Bas.**	*Reference*		
	**SAM**	0.00	0.985	-0.47, 0.46	**Dis.**	0.09	0.676	-0.33, 0.51	**SAM**	0.15	0.744	-0.73, 1.02	**Dis.**	0.09	0.689	-0.33, 0.51
					**12w**	0.89	<0.001	0.49, 1.30					**12w**	0.89	<0.001	0.49, 1.30
					**24w**	0.6	0.004	0.19, 1.02					**24w**	0.60	0.004	0.19, 1.02
					**48w**	-0.62	0.002	-1.02, -0.22					**48w**	-0.63	0.002	-1.03, -0.23
**PC4**	**Healthy**	*Reference*			**Bas.**	*Reference*			**Healthy**	*Reference*			**Bas.**	*Reference*		
	**SAM**	-0.18	0.318	-0.52, 0.17	**Dis.**	-0.04	0.828	-0.40, 0.32	**SAM**	-0.09	0.771	-0.71, 0.53	**Dis.**	-0.05	0.804	-0.40, 0.31
					**12w**	0.16	0.355	-0.18, 0.50					**12w**	0.16	0.354	-0.18, 0.50

					**24w**	0.59	0.001	0.24, 0.94					**24w**	0.59	0.001	0.24, 0.94
					**48w**	-0.18	0.290	-0.52, 0.16					**48w**	-0.19	0.283	-0.53, 0.15
**PC5**	**Healthy**	*Reference*			**Bas.**	*Reference*			**Healthy**	*Reference*			**Bas.**	*Reference*		
	**SAM**	0.31	0.044	0.01, 0.61	**Dis.**	0.06	0.708	-0.25, 0.36	**SAM**	0.47	0.069	-0.04, 0.98	**Dis.**	0.08	0.599	-0.22, 0.38
					**12w**	0.04	0.769	-0.25, 0.34					**12w**	0.06	0.707	-0.23, 0.35
					**24w**	-0.19	0.208	-0.49, 0.11					**24w**	-0.20	0.186	-0.50, 0.10
					**48w**	-0.43	0.003	-0.72, -0.14					**48w**	-0.44	0.003	-0.73, -0.15

Supplementary Table 15. Mixed linear regression of anti-bacterial innate immune cell function principal component (PC) scores for children who have recovered by SAM group and visit as fixed effects and participant identifier and SAM group as random effects (recovered cases n=67, controls n=73).

All variables censored at 0 and log(n+1) transformed.

Dis. – discharge (n=38), 12wk – 12 week post-discharge visit (n=47), 24wk – 24 week post-discharge visit (n=43), 48wk – 48 week post-discharge visit (n=49)

[1]SAM>WN – all cases who were well-nourished (WHZ> -2) at each follow-up visit (recovered cases)

	Unadjusted								Adjusted (country, sex, age group at enrolment, HIV status, cerebral palsy, baseline oedema, baseline WHZ)							
	SAM status (discharge)				SAM status*Visit				SAM status (discharge)				SAM status*Visit			
		Coef.	p	95% CI		Coef.	p	95% CI		Adj. Coef.	p	95% CI		Adj. Coef.	p	95% CI
**PC1**	**Healthy**	*Reference*			**Dis.**	*Reference*			**Healthy**	*Reference*			**Dis.**	*Reference*		
	**SAM>WN**[1]	2.98	<0.001	2.03, 3.93	**12w**	-0.56	0.184	-1.39, 0.27	**SAM>WN**	2.47	0.003	0.84, 4.11	**12w**	-0.63	0.134	-1.45, 0.19
					**24w**	-1.24	0.004	-2.08, -0.41					**24w**	-1.22	0.004	-2.05, -0.39
					**48w**	-1.86	<0.001	-2.68, -1.04					**48w**	-1.84	<0.001	-2.66, -1.03
**PC2**	**Healthy**	*Reference*			**Dis.**	*Reference*			**Healthy**	*Reference*			**Dis.**	*Reference*		
	**SAM>WN**	-1.10	0.003	-1.82, -0.38	**12w**	1.57	<0.001	0.95, 2.18	**SAM>WN**	-1.88	0.004	-3.17, -0.59	**12w**	1.52	<0.001	0.91, 2.13
					**24w**	1.36	<0.001	0.74, 1.98					**24w**	1.36	<0.001	0.74, 1.97
					**48w**	1.97	<0.001	1.37, 2.58					**48w**	1.97	<0.001	1.36, 2.57
**PC3**	**Healthy**	*Reference*			**Dis.**	*Reference*			**Healthy**	*Reference*			**Dis.**	*Reference*		
	**SAM>WN**	-0.25	0.374	-0.79, 0.30	**12w**	1.35	<0.001	0.95, 1.76	**SAM>WN**	-0.17	0.741		**12w**	1.37	<0.001	0.96, 1.78
					**24w**	0.67	0.001	0.26, 1.09					**24w**	0.68	0.001	0.27, 1.09
					**48w**	-0.26	0.198	-0.67, 0.14					**48w**	-0.26	0.198	-0.67, 0.14
**PC4**	**Healthy**	*Reference*			**Dis.**	*Reference*			**Healthy**	*Reference*			**Dis.**	*Reference*		
	**SAM>WN**	0.11	0.573	-0.27, 0.49	**12w**	-0.19	0.224	-0.49, 0.11	**SAM>WN**	0.34	0.346	-0.37, 1.06	**12w**	-0.19	0.227	-0.49, 0.12
					**24w**	0.35	0.023	0.05, 0.66					**24w**	0.36	0.021	0.05, 0.67
					**48w**	-0.40	0.009	-0.70, -0.10					**48w**	-0.39	0.011	-0.69, -0.09
**PC5**	**Healthy**	*Reference*			**Dis.**	*Reference*			**Healthy**	*Reference*			**Dis.**	*Reference*		
	**SAM>WN**	0.37	0.040	0.02, 0.73	**12w**	0.01	0.973	-0.33, 0.34	**SAM>WN**	0.48	0.118	-0.12, 1.08	**12w**	0.04	0.800	-0.29, 0.37
					**24w**	-0.16	0.356	-0.49, 0.18					**24w**	-0.15	0.393	-0.48, 0.19
					**48w**	-0.42	0.012	-0.75, -0.09					**48w**	-0.41	0.014	-0.74, -0.08
