# Supplementary material for: Restoration of anti-bacterial innate immune cell function lags behind nutritional recovery among children convalescing from complicated severe acute malnutrition
Source: Nat Commun. 2026 Jul 28;17:9172. doi: 10.1038/s41467-026-75897-7 (PMC13521902; doi:10.1038/s41467-026-75897-7)
Supplement: Supplementary file 2 [file 41467_2026_75897_MOESM2_ESM.pdf]

nature portfolio

Corresponding author(s): Claire D. Bourke

Last updated by author(s): 15/06/2026

Reporting Summary

Nature Portfolio wishes to improve the reproducibility of the work that we publish. This form provides structure for consistency and transparency in reporting. For further information on Nature Portfolio policies, see our Editorial Policies and the Editorial Policy Checklist.

Statistics

For all statistical analyses, confirm that the following items are present in the figure legend, table legend, main text, or Methods section.

n/a	Confirmed	
☐	☒	The exact sample size (*n*) for each experimental group/condition, given as a discrete number and unit of measurement
☐	☒	A statement on whether measurements were taken from distinct samples or whether the same sample was measured repeatedly
☐	☒	The statistical test(s) used AND whether they are one- or two-sided<br>*Only common tests should be described solely by name; describe more complex techniques in the Methods section.*
☐	☒	A description of all covariates tested
☐	☒	A description of any assumptions or corrections, such as tests of normality and adjustment for multiple comparisons
☐	☒	A full description of the statistical parameters including central tendency (e.g. means) or other basic estimates (e.g. regression coefficient) AND variation (e.g. standard deviation) or associated estimates of uncertainty (e.g. confidence intervals)
☐	☒	For null hypothesis testing, the test statistic (e.g. *F*, *t*, *r*) with confidence intervals, effect sizes, degrees of freedom and *P* value noted<br>*Give P values as exact values whenever suitable.*
☒	☐	For Bayesian analysis, information on the choice of priors and Markov chain Monte Carlo settings
☒	☐	For hierarchical and complex designs, identification of the appropriate level for tests and full reporting of outcomes
☒	☐	Estimates of effect sizes (e.g. Cohen's *d*, Pearson's *r*), indicating how they were calculated

Our web collection on statistics for biologists contains articles on many of the points above.

Software and code

Policy information about availability of computer code

Data collection	No custom code was used.
Data analysis	All statistical analyses were performed using STATA version 18 (StataCorp LP, USA). Outcome variables were plotted using Prism version 10 (GraphPad Software Inc., USA) STATA, and R version 4. Nutritional trajectories were plotted using https://sankeymatic.com/build/.

For manuscripts utilizing custom algorithms or software that are central to the research but not yet described in published literature, software must be made available to editors and reviewers. We strongly encourage code deposition in a community repository (e.g. GitHub). See the Nature Portfolio guidelines for submitting code & software for further information.

Data

Policy information about availability of data

All manuscripts must include a data availability statement. This statement should provide the following information, where applicable:

- Accession codes, unique identifiers, or web links for publicly available datasets
- A description of any restrictions on data availability
- For clinical datasets or third party data, please ensure that the statement adheres to our policy

The dataset from the immunology sub-study of HOPE-SAM (IMMUNO-SAM) reported in this article is available at 10.6084/m9.figshare.32678214. Source Data is provided for each figure.

Research involving human participants, their data, or biological material

Policy information about studies with human participants or human data. See also policy information about sex, gender (identity/presentation), and sexual orientation and race, ethnicity and racism.

Reporting on sex and gender	The study focuses on immune function variables for children under 5 years old and all reporting refers only to sex rather than to gender. Children were not recruited or stratified on the basis of sex in the IMMUNO-SAM study design. However, as sex can influence the immune system from an early age, multivariable linear regression models adjust for sex as a covariate.
Reporting on race, ethnicity, or other socially relevant groupings	The study focuses on immune function variables of children recruited in Zambia and Zimbabwe all of whom were black Africans; study participants are referred to according to their country of recruitment throughout.
Population characteristics	The population of children included in this study were participants in the Health Outcomes and Pathogenesis of Severe Acute Malnutrition (HOPE-SAM) longitudinal observational cohort study. Children under 5 years old who were hospitalised for complicated SAM at three hospitals in Lusaka, Zambia and Harare, Zimbabwe between August 2016 and March 2018. SAM was defined per WHO definitions as WHZ<−3, MUAC<115 mm, nutritional oedema or both for children above 6 months of age, or WHZ<−3, nutritional oedema or both for those aged under 6 months.<br><br>Children aged 6-59 months who were adequately-nourished (WHZ>–1) and clinically well (no symptoms of acute illness or current infections, no underlying chronic gastrointestinal disease) were recruited from the same hospitals as the cases as a context-relevant control group; children were not excluded on the basis of their HIV status or linear growth (e.g. height-for-age Z score). Biological samples were collected from controls at enrollment (baseline) only. To be eligible for enrollment as a case or control, children needed to have a known HIV status.<br><br>For inclusion in this immunology sub-study (IMMUNO-SAM), HOPE-SAM participants needed to have enrolled into HOPE-SAM after ethical approval of IMMUNO-SAM procedures and provided a ≥2mL blood sample at any time during their hospitalisation (cases) or at enrolment (controls). Selection of the IMMUNO-SAM cohort is summarised in Figure 1; full details in Supplementary Figure 1. The IMMUN0-SAM cohort included 125 children hospitalised with SAM (mean age: 18.6 months, SD 8.6; 66 male, 59 female) and 73 adequately-nourished non-hospitalised controls (mean age: 30.3 months, SD: 15.7; 38 male, 35 female).
Recruitment	Recruitment of cases and controls was in-person by the HOPE-SAM study team at three hospitals in Lusaka (University Teaching Hospital), Zambia and Harare (Harare Central Hospital and Parirenyatwa Hospital), Zimbabwe between August 2016 and March 2018. Caregivers of eligible children were informed of the study and, where they provided written-informed consent for their child's participation in the study. Children were enrolled as soon as possible after hospital admission (baseline).<br><br>Requirement for a blood sample for inclusion in the IMMUNO-SAM sub-study meant that children who were too unwell to provide a blood sample (e.g. severe anaemia, collapsed vein) or for whom caregivers refused consent for blood sampling were not included. Children in the IMMUNO-SAM cohort experienced fewer adverse clinical outcomes than those enrolled in HOPE-SAM where a blood sample was not an eligibility criterion.
Ethics oversight	Ethical approval for HOPE-SAM was obtained from the University of Zambia Biomedical Research Ethics Committee, and the Medical Research Council of Zimbabwe. The ethics committee of the Queen Mary University of London provided an advisory review (QMUL reference: QMREC2015/17). Procedures for IMMUNO-SAM were added as an amendment to the HOPE-SAM protocol and approved on the 18th August 2017 (HOPE-SAM protocol version 3, 10/07/2017). This study adhered to all relevant ethical regulations. Caregivers provided written informed consent for the participation of their children in this study.

Note that full information on the approval of the study protocol must also be provided in the manuscript.

Field-specific reporting

Please select the one below that is the best fit for your research. If you are not sure, read the appropriate sections before making your selection.

[x] Life sciences [ ] Behavioural & social sciences [ ] Ecological, evolutionary & environmental sciences

For a reference copy of the document with all sections, see nature.com/documents/nr-reporting-summary-flat.pdf

Life sciences study design

All studies must disclose on these points even when the disclosure is negative.

Sample size	A sample size was determined for the parent observational cohort study (HOPE-SAM) based on post-discharge mortality (primary clinical outcome). The observational cohort aimed to enrol ≤800 children with complicated SAM. We estimated that a mortality of 15% and overall loss to follow-up of 15% would provide 560 evaluable children at 1 y, of whom 224 would have HIV-SAM based on an estimated prevalence of 40% in hospitalized children (17). This would provide >80% power at 5% significance to detect absolute differences of 17% in binary outcomes between HIV-positive and HIV-negative children with SAM, and of 0.33 SDs in continuous outcomes.<br><br>At the time our immunology sub-study (IMMUNO-SAM) was conducted, there was no previous study assessing the immune cell function outcomes that we were evaluating in children with SAM from which to calculate a realistic sample size. We therefore chose to include all

	children enrolled in the HOPE-SAM study who met the inclusion criteria for IMMUNO-SAM as well as all follow-up samples available from these children over the post-discharge clinical follow-up period (48 weeks).
Data exclusions	IMMUNO-SAM was conducted using longitudinal blood samples (1 mL/kg up to a maximum of 5.4 mL) collected into EDTA-treated endotoxin-free blood collection tubes at the baseline, discharge, 12, 24 and 48 week study visit. Blood was not collected from children known to have severe anaemia (haemoglobin <60 g/L). For inclusion in the IMMUNO-SAM sub-study, HOPE-SAM participants needed to have enrolled into HOPE-SAM after ethical approval of IMMUNO-SAM procedures and provided a ≥2mL blood sample at any time during their hospitalisation (cases) or at enrolment (controls). Children who died, defaulted or exited the study or for whom there was no available blood sample or no known HIV status were excluded. Selection of the IMMUNO-SAM cohort is summarised in Figure 1; full details in Supplementary Figure 1.
Replication	The study reports results from a single observational cohort study with individual children enrolled in the study as biological replicates (n=125 cases, n=73 controls).
Randomization	This was an observational study without random allocation. Full details of multivariable linear regression models, adjusting for relevant covariates/confounders, are described in the materials and methods and indicated in figure and table legends.
Blinding	This was an observational study without blinding.

Reporting for specific materials, systems and methods

We require information from authors about some types of materials, experimental systems and methods used in many studies. Here, indicate whether each material, system or method listed is relevant to your study. If you are not sure if a list item applies to your research, read the appropriate section before selecting a response.

Materials & experimental systems

n/a	Involved in the study
☐	☒ Antibodies
☐	☐ Eukaryotic cell lines
☐	☐ Palaeontology and archaeology
☐	☐ Animals and other organisms
☐	☒ Clinical data
☐	☐ Dual use research of concern
☐	☐ Plants

Methods

n/a	Involved in the study
☐	☐ ChIP-seq
☐	☒ Flow cytometry
☐	☐ MRI-based neuroimaging

Antibodies

Antibodies used	Stored bacterial binding assay cell samples were labelled with: Lin (CD3, CD19, CD20, CD56)-APC (10µL/tube, clones: clones: UCHT1, HIB19, 2H7, 5.1H11; Cat# 363601), CD66b-PerCPCy5.5 (0.5µL/tube, clone: G10F5; Cat#305108), CD16-APCCy7 (2.5µL/tube, clone: 3G8; Cat# 302018), CD14-PE (2.5µL/tube, clone: HCD14; Cat# 325606, all Biolegend, UK) and HLA-DR-PECy7 (2.5µL/tube, clone: L243 (G46-6); Cat# 560651, BD Biosciences, UK). Whole blood culture cell samples were labelled with: Lin (CD3, CD19, CD20, CD56)-APC, CD66b-APC (0.5µL/tube, clone: G10F5; Cat# 305118), CD16-APCCy7, CD14-PE, CD86-FITC (5µL/tube, clone: BU63; Cat# 374204), CD40-PerCPCy5.5 (2.5µL/tube, clone: 5C3; Cat# 334316, all Biolegend, UK) and HLA-DR-PECy7 (BD Biosciences, UK).
Validation	All antibodies used were purchased as commercial products from Biolegend and BD Biosciences; both companies provide validation statements and relevant references external to our work on their respective antibody product pages (refer to catalogue numbers provided above).

Eukaryotic cell lines

Policy information about cell lines and Sex and Gender in Research

Cell line source(s)	*State the source of each cell line used and the sex of all primary cell lines and cells derived from human participants or vertebrate models.*
Authentication	*Describe the authentication procedures for each cell line used OR declare that none of the cell lines used were authenticated.*
Mycoplasma contamination	*Confirm that all cell lines tested negative for mycoplasma contamination OR describe the results of the testing for mycoplasma contamination OR declare that the cell lines were not tested for mycoplasma contamination.*
Commonly misidentified lines (See ICLAC register)	*Name any commonly misidentified cell lines used in the study and provide a rationale for their use.*

Palaeontology and Archaeology

Specimen provenance	*Provide provenance information for specimens and describe permits that were obtained for the work (including the name of the issuing authority, the date of issue, and any identifying information). Permits should encompass collection and, where applicable, export.*
Specimen deposition	*Indicate where the specimens have been deposited to permit free access by other researchers.*
Dating methods	*If new dates are provided, describe how they were obtained (e.g. collection, storage, sample pretreatment and measurement), where they were obtained (i.e. lab name), the calibration program and the protocol for quality assurance OR state that no new dates are provided.*

☐ Tick this box to confirm that the raw and calibrated dates are available in the paper or in Supplementary Information.

Ethics oversight	*Identify the organization(s) that approved or provided guidance on the study protocol, OR state that no ethical approval or guidance was required and explain why not.*

Note that full information on the approval of the study protocol must also be provided in the manuscript.

Animals and other research organisms

Policy information about studies involving animals; ARRIVE guidelines recommended for reporting animal research, and Sex and Gender in Research

Laboratory animals	*For laboratory animals, report species, strain and age OR state that the study did not involve laboratory animals.*
Wild animals	*Provide details on animals observed in or captured in the field; report species and age where possible. Describe how animals were caught and transported and what happened to captive animals after the study (if killed, explain why and describe method; if released, say where and when) OR state that the study did not involve wild animals.*
Reporting on sex	*Indicate if findings apply to only one sex; describe whether sex was considered in study design, methods used for assigning sex. Provide data disaggregated for sex where this information has been collected in the source data as appropriate; provide overall numbers in this Reporting Summary. Please state if this information has not been collected. Report sex-based analyses where performed, justify reasons for lack of sex-based analysis.*
Field-collected samples	*For laboratory work with field-collected samples, describe all relevant parameters such as housing, maintenance, temperature, photoperiod and end-of-experiment protocol OR state that the study did not involve samples collected from the field.*
Ethics oversight	*Identify the organization(s) that approved or provided guidance on the study protocol, OR state that no ethical approval or guidance was required and explain why not.*

Note that full information on the approval of the study protocol must also be provided in the manuscript.

Clinical data

Policy information about clinical studies
All manuscripts should comply with the ICMJE guidelines for publication of clinical research and a completed CONSORT checklist must be included with all submissions.

Clinical trial registration	This study and the parent clinical study (HOPE-SAM) are observational and not clinical trials
Study protocol	The HOPE-SAM study protocol, including the immunology sub-study (IMMUNO-SAM), has been published in BMJ Open: Bwakura-Dangarembizi M, Amadi B, Bourke CD, et alHealth Outcomes, Pathogenesis and Epidemiology of Severe Acute Malnutrition (HOPE-SAM): rationale and methods of a longitudinal observational studyBMJ Open 2019;9:e023077. doi: 10.1136/bmjopen-2018-023077
Data collection	The protocol, standard operating procedures, and case report forms used for the HOPE-SAM study are available at https://osf.io/29uaw/
Outcomes	For this manuscript, primary outcomes were anti-bacterial innate immune cell function read outs from two in vitro assays: 1h Bacterial binding assay (proportions of E.coli-positive cells and mean fluorescence intensity for E.coli as assessed by flow cytometry and partitioned by immune cell type) and 24h whole blood culture (median fluorescence intensity of cell surface markers by monocyte subsets assessed by flow cytometry and concentrations (pg/mL) of anti-bacterial protein mediators in culture supernatants quantified by ELISA)

Dual use research of concern

Policy information about dual use research of concern

Hazards

Could the accidental, deliberate or reckless misuse of agents or technologies generated in the work, or the application of information presented in the manuscript, pose a threat to:

No	Yes	
☒	☐	Public health
☒	☐	National security
☒	☐	Crops and/or livestock
☒	☐	Ecosystems
☒	☐	Any other significant area

Experiments of concern

Does the work involve any of these experiments of concern:

No	Yes	
☒	☐	Demonstrate how to render a vaccine ineffective
☒	☐	Confer resistance to therapeutically useful antibiotics or antiviral agents
☒	☐	Enhance the virulence of a pathogen or render a nonpathogen virulent
☒	☐	Increase transmissibility of a pathogen
☒	☐	Alter the host range of a pathogen
☒	☐	Enable evasion of diagnostic/detection modalities
☒	☐	Enable the weaponization of a biological agent or toxin
☒	☐	Any other potentially harmful combination of experiments and agents

Plants

Seed stocks	*Report on the source of all seed stocks or other plant material used. If applicable, state the seed stock centre and catalogue number. If plant specimens were collected from the field, describe the collection location, date and sampling procedures.*
Novel plant genotypes	*Describe the methods by which all novel plant genotypes were produced. This includes those generated by transgenic approaches, gene editing, chemical/radiation-based mutagenesis and hybridization. For transgenic lines, describe the transformation method, the number of independent lines analyzed and the generation upon which experiments were performed. For gene-edited lines, describe the editor used, the endogenous sequence targeted for editing, the targeting guide RNA sequence (if applicable) and how the editor was applied.*
Authentication	*Describe any authentication procedures for each seed stock used or novel genotype generated. Describe any experiments used to assess the effect of a mutation and, where applicable, how potential secondary effects (e.g. second site T-DNA insertions, mosiacism, off-target gene editing) were examined.*

ChIP-seq

Data deposition

☐ Confirm that both raw and final processed data have been deposited in a public database such as GEO.

☐ Confirm that you have deposited or provided access to graph files (e.g. BED files) for the called peaks.

Data access links<br>*May remain private before publication.*	*For "Initial submission" or "Revised version" documents, provide reviewer access links. For your "Final submission" document, provide a link to the deposited data.*
Files in database submission	*Provide a list of all files available in the database submission.*
Genome browser session<br>(e.g. UCSC)	*Provide a link to an anonymized genome browser session for "Initial submission" and "Revised version" documents only, to enable peer review. Write "no longer applicable" for "Final submission" documents.*

Methodology

Replicates	*Describe the experimental replicates, specifying number, type and replicate agreement.*
Sequencing depth	*Describe the sequencing depth for each experiment, providing the total number of reads, uniquely mapped reads, length of reads and whether they were paired- or single-end.*
Antibodies	*Describe the antibodies used for the ChIP-seq experiments; as applicable, provide supplier name, catalog number, clone name, and lot number.*
Peak calling parameters	*Specify the command line program and parameters used for read mapping and peak calling, including the ChIP, control and index files used.*

Data quality	*Describe the methods used to ensure data quality in full detail, including how many peaks are at FDR 5% and above 5-fold enrichment.*
Software	*Describe the software used to collect and analyze the ChIP-seq data. For custom code that has been deposited into a community repository, provide accession details.*

Flow Cytometry

Plots

Confirm that:

- [x] The axis labels state the marker and fluorochrome used (e.g. CD4-FITC).
- [x] The axis scales are clearly visible. Include numbers along axes only for bottom left plot of group (a 'group' is an analysis of identical markers).
- [x] All plots are contour plots with outliers or pseudocolor plots.
- [x] A numerical value for number of cells or percentage (with statistics) is provided.

Methodology

Sample preparation	IMMUNO-SAM was conducted using longitudinal blood samples from children with SAM (1mL/kg up to a maximum of 5.4mL) collected into EDTA-treated endotoxin-free blood collection tubes at the baseline, discharge, 12, 24 and 48 week study visit. A single blood sample was collected in the same way from controls at baseline. Blood samples were processed for immune cell function assays (bacterial binding assay and whole blood culture assay) immediately upon receipt into the laboratory. Cultured cells were then treated with 1x FACS lysing and fixative buffer (BD Biosciences, UK) for 10mins, washed in phosphate-buffered saline (PBS), resuspended in PBS with 10% v/v dimethyl sulfoxide (DMSO) 40% v/v heat-inactivated foetal bovine serum (FBS), and gradually frozen at 1oC/min to -80°C using a Mr. Frosty freezing container.
Instrument	Cells were analysed on a 6-colour BD Biosciences FACSVerse flow cytometer (488nm and 633nm lasers).
Software	FlowJo version 10.8.1 (FlowJo LLC, USA)
Cell population abundance	Cells were not sorted prior to immunophenotyping.
Gating strategy	Full gating strategies for IMMUNO-SAM have been reported in a previous publication which is Open Access and signposted in the Materials and Methods:<br>Phiri et al. Severe acute malnutrition promotes bacterial binding over proinflammatory cytokine secretion by circulating innate immune cells. Sci Adv. 2023 Nov 3;9(44):eadh2284. doi: 10.1126/sciadv.adh2284

- [x] Tick this box to confirm that a figure exemplifying the gating strategy is provided in the Supplementary Information.

Magnetic resonance imaging

Experimental design

Design type	*Indicate task or resting state; event-related or block design.*
Design specifications	*Specify the number of blocks, trials or experimental units per session and/or subject, and specify the length of each trial or block (if trials are blocked) and interval between trials.*
Behavioral performance measures	*State number and/or type of variables recorded (e.g. correct button press, response time) and what statistics were used to establish that the subjects were performing the task as expected (e.g. mean, range, and/or standard deviation across subjects).*

Acquisition

Imaging type(s)	*Specify: functional, structural, diffusion, perfusion.*
Field strength	*Specify in Tesla*
Sequence & imaging parameters	*Specify the pulse sequence type (gradient echo, spin echo, etc.), imaging type (EPI, spiral, etc.), field of view, matrix size, slice thickness, orientation and TE/TR/flip angle.*
Area of acquisition	*State whether a whole brain scan was used OR define the area of acquisition, describing how the region was determined.*

Diffusion MRI ☐ Used ☐ Not used

Preprocessing

Preprocessing software	*Provide detail on software version and revision number and on specific parameters (model/functions, brain extraction, segmentation, smoothing kernel size, etc.).*

Normalization

If data were normalized/standardized, describe the approach(es): specify linear or non-linear and define image types used for transformation OR indicate that data were not normalized and explain rationale for lack of normalization.

Normalization template

Describe the template used for normalization/transformation, specifying subject space or group standardized space (e.g. original Talairach, MNI305, ICBM152) OR indicate that the data were not normalized.

Noise and artifact removal

Describe your procedure(s) for artifact and structured noise removal, specifying motion parameters, tissue signals and physiological signals (heart rate, respiration).

Volume censoring

Define your software and/or method and criteria for volume censoring, and state the extent of such censoring.

Statistical modeling & inference

Model type and settings

Specify type (mass univariate, multivariate, RSA, predictive, etc.) and describe essential details of the model at the first and second levels (e.g. fixed, random or mixed effects; drift or auto-correlation).

Effect(s) tested

Define precise effect in terms of the task or stimulus conditions instead of psychological concepts and indicate whether ANOVA or factorial designs were used.

Specify type of analysis: ☐ Whole brain ☐ ROI-based ☐ Both

Statistic type for inference

(See Eklund et al. 2016)

Specify voxel-wise or cluster-wise and report all relevant parameters for cluster-wise methods.

Correction

Describe the type of correction and how it is obtained for multiple comparisons (e.g. FWE, FDR, permutation or Monte Carlo).

Models & analysis

n/a	Involved in the study
☐	☐ Functional and/or effective connectivity
☐	☐ Graph analysis
☐	☐ Multivariate modeling or predictive analysis

Functional and/or effective connectivity

Report the measures of dependence used and the model details (e.g. Pearson correlation, partial correlation, mutual information).

Graph analysis

Report the dependent variable and connectivity measure, specifying weighted graph or binarized graph, subject- or group-level, and the global and/or node summaries used (e.g. clustering coefficient, efficiency, etc.).

Multivariate modeling and predictive analysis

Specify independent variables, features extraction and dimension reduction, model, training and evaluation metrics.
