# Supplementary material for: Restoration of anti-bacterial innate immune cell function lags behind nutritional recovery among children convalescing from complicated severe acute malnutrition
Source: Nat Commun. 2026 Jul 28;17:9172. doi: 10.1038/s41467-026-75897-7 (PMC13521902; doi:10.1038/s41467-026-75897-7)
Supplement: Supplementary file 3 [file 41467_2026_75897_MOESM3_ESM.pdf]

nature portfolio

Peer Review File

Restoration of anti-bacterial innate immune cell function lags behind nutritional recovery among children convalescing from complicated severe acute malnutrition

Corresponding Author: Dr Claire Bourke

This file contains all reviewer reports in order by version, followed by all author rebuttals in order by version.

Version 0:

Reviewer comments:

Reviewer #1

(Remarks to the Author)
● What are the noteworthy results?
The noteworthy results of this paper are that children hospitalized with acute SAM have increased innate immune cell sensitivity to bacterial binding, decreased pro-inflammatory cytokine response, and impaired monocyte marker expression as compared to healthy controls from their communities. Over the course of 48 weeks of followup, this dysregulated profile generally approached a more recovered profile more similar to the controls. However, even after nutritional recovery, several metrics of innate immune function remained distinct in cases vs. controls, suggesting that full immune recovery takes longer than nutritional recovery. A stronger pro-inflammatory cytokine response was associated with better nutritional recovery, but variation in innate immune function in SAM patients was not associated with adverse clinical outcomes.
● Will the work be of significance to the field and related fields? How does it compare to the established literature? If the work is not original, please provide relevant references.
Yes, this work highlights the extended vulnerability of children with complicated SAM to infection beyond their nutritional recovery, which has direct clinical implications. While SAM-associated vulnerability to infection has long been recognized, this study provides more specific mechanistic insight into SAM-associated immune system changes, and their connections to clinical outcomes. This study also explores the question of whether adverse clinical outcomes in a subset of SAM patients are associated with differences in innate immune function, finding that they were not.
● Does the work support the conclusions and claims, or is additional evidence needed?
The work does support the conclusions and claims.
● Are there any flaws in the data analysis, interpretation and conclusions? Do these prohibit publication or require revision?
No apparent flaws in data analysis. A more thorough interpretation of the differences observed in bacterial binding sensitivity, and a consideration/refuation of potential seasonal effects would strengthen the interpretation/limitations section.
● Is the methodology sound? Does the work meet the expected standards in your field?
The methodology appears sound.
● Is there enough detail provided in the methods for the work to be reproduced?
Yes
Major Comments:
Line 7: Describe and explicitly define "complicated SAM" and the common clinical complications associated with SAM for the purposes of being included in this cohort. While many complications are mentioned, it is unclear whether these fall under the category of "complicated SAM" for the purposes of this study. Perhaps these complications could also be listed in Table 1.

Line 27-28: Clarify meaning of phrase: "implicate a role for the immune system in SAM pathogenesis." Is the intended meaning that the immune system might play a causal role in SAM? Or is the intended meaning that SAM-induced immune system changes play a role in SAM-related mortality and morbidity? Current wording - esp. "pathogenesis" implies the former, but it seems like the latter fits more with the findings of the study.

Introduction, Results, Discussion: When discussing findings of this study and related studies, reconsider repeated use of vague nouns like " shifts in anti-bacterial innate immune cell function" (Line 43), or "sustained differences in anti-bacterial innate immune cell function" (Line 436-437). When possible, specify the specific changes referenced – perhaps increased

sensitivity to bacterial binding, or decreased pro-inflammatory cytokine response, or impaired monocyte marker expression. While the instinct to use words like “shifts”, “dysregulated”, or “differences” is understandable given the range of changes observed, adding brief elements of specificity when appropriate would contribute to improved clarity and usefulness to readers. Similarly, outlining the “dysregulated” immune status vs. the “recovered” immune status with more clarity would serve a similar purpose. In Results section headings and figure titles, a few more directional words to clarify “normalizes” would also be helpful.

Discussion: While the altered intermediate/classical/non-classical ratios of monocytes were well-explained and a possible mechanism was provided, such an explanation for the observed increase in bacterial binding capacity was not provided in the discussion. Any insights into the potential mechanisms or implications of this change, especially if any new ideas have been generated since the previous work showing this finding, would be a valuable addition to the paper.

Line 357-369: This crucial insight would be better served at the conclusion of the Discussion section. In its current placement at the end of a paragraph about bacterial binding capacity, it seems out of place and its impact is lessened.

Limitations: Consider the potential influence of seasonality on the immune metrics measured in this study, and whether they were accounted for in study enrollment. Seasonal variations in innate immune response have been observed in other populations. The collection of a singular control blood sample at baseline leaves open the possibility that some fraction of changes attributed to recovery in this study could also be influenced by seasonality, unless the timing of collection varied enough to preclude this.

Figure 4: This figure has too many panels and is hard to read. Panels J and N overlap with the adjacent panels, making them look messy. Consider pruning this group to just LPS or just HKST, or else reducing to some other representative group and including the full group in supplemental figures.

Figure 5: Reconsider overlap with Figures 3, 4, and 6 in main figures. Figure 5 has good parallelism with the corresponding section in Results, but only the first two panels are relevant to the narrative function of this figure as it is currently presented. The information in this figure’s title also seems to be already provided in figures 3 and 4. It is also remarkably similar in structure to Figure 6 – is it possible to combine them in some way (like using color coding of “recovered” children + listing of respective statistics in a table format)? Given the abundance of figures in the supplement, and the relative importance and explanatory power of some of them (especially supplementary figure 10), perhaps the space is better given to another figure.

Minor Comments:
Line 3-6: Adjust wording/rearrange clauses to improve readability and reduce nested parentheticals. “Affecting nearly twenty million children globally, severe acute malnutrition (SAM) is a form of undernutrition identified by severe wasting, nutritional oedema, or both. Clinically, severe wasting is defined by weight-for-height Z-scores (WHZ) <-3 and/or mid-upper arm circumference (MUAC) <115mm.

Line 191-193: change wording to clarify meaning and add period. Suggest changing to “Thus, dysregulated circulating monocyte phenotypes and pro-inflammatory cytokine secretion in cases at hospital admission appear to normalise with time post-discharge.

Line 371-375: “Total monocytes…” sentence is hard to follow. Consider splitting into two sentences. Phrase “that decreased and increased respectively during follow-up” appears to be a fragment – consider removing “that”

Line 403: Missed word, add “children recovering from SAM”

Line 436 - 438: Sentence is hard to follow; recommend changing to make the subject more clear e.g. “In children hospitalized with complicated SAM, we found sustained differences in anti-bacterial innate immune cell function over a 48-week post-discharge follow-up, as compared with healthy controls”

Line 982: Extra “innate” in title

Reviewer #2

(Remarks to the Author)
In the manuscript, “Restoration of anti-bacterial innate immune function lags behind nutritional recovery among children convalescing from complicated severe acute malnutrition” authors Rukobo et al study a disease of global importance, namely severe acute malnutrition which is associated with considerable post-discharge/recovery mortality. The manuscript uses a cross sectional and longitudinal study design to assess changes in anti-bacterial innate immune function in children with SAM relative to their clinical outcomes and subsequent ponderal growth changes. The study subject is important as it provides insights into what immune vulnerabilities children with SAM may have and may keep in their recovery trajectory. The authors find that bacterial binding capacity decrease and pro-inflammatory cytokines increase with normalization of growth, but that these disruption persists despite recovery from SAM.

General comments

In this manuscript the authors are extending findings they made in a previous publication (Phiri TN, et al. Severe acute malnutrition promotes bacterial binding over proinflammatory cytokine secretion by circulating innate immune cells. Sci Adv,

2023) where they found that clinically admitted children with SAM had high bacterial binding capacity that associated with persistent SAM at time of hospital discharge. They now report on follow-up and testing of a subset of these children, assessing associations with clinical outcomes, ponderal growth over time and in comparison to a community control group. In this way, the study provides incremental gains but do deepen understanding of earlier reported findings.

The major questions/weaknesses for the manuscript are on the study design and implementation, and statistical analyses.

In their first analyses (Line 102-3), the authors assess whether inpatient immune function differs between children with and without adverse clinical outcomes post-discharge. However, methodology for collecting clinical events data during follow up is of low quality, precluding meaningful assessment of these outcomes. For example, recall is used with study visits spaced more than 12 and 20 week intervals, making it highly unlikely that there is accurate capture of clinical events. In addition, there is no differentiation in the analyses between infections/possibly infections and non-infectious clinical events, which would be relevant to these immune outcomes. In addition, the study has very poor follow up (only 60-70%) with minimal criteria (> 1 follow-up visit) for inclusion in the longitudinal cohort. These major methodologic limitations preclude any meaningful interpretation of the clinical outcomes data as reported in Figure 2). These same issues should be addressed for the immune assessments in the longitudinal cohort, where follow-up was similarly poor for immune analyses. How did the authors deal with these issues in their analyses?

Another major weakness in the study design is that there were no follow ups for the control arm children, complicating comparisons with cases by agen and over time. This is important as there are likely age-dependent changes in immune function in healthy children. Could the authors assess age within the control group and whether there are any alterations in immune function by age that would be confounding results over time in the case group.

In their analyses, the authors employ PCA and use variation on the PC axes to further assess changes over time. The biologic relevance of these axes is sometimes questionable. The authors could better address and/or explain this.

Finally, it would be helpful if the authors clarified some of their hypotheses. There are two related but ultimately separate hypotheses in the paper – first that immune dysfunction could help explain post discharge clinical outcomes, second, that immune dysfunction observed at the time of admission for SAM may persist despite nutritional recovery. These are not the same pathophysiologically or mechanistically. Suggest that the authors clarify the two in text.

Specific comments below
Table 1
There are significant differences between the community controls and the SAM inpatients, most importantly, age and HIV status which could cause significant confounding in their results. Did the authors consider age matching cases to controls. What was the rationale ror combining (line 946) the < 6 months and 6-11 month age groups?

This sentence is confusing: "number of re-admissions among re-admitted children"
The N's for each of the clinical outcomes is also unclear. For example clinic visit for reasons outside normal schedule, is n the population that had an extra clinic visit, or the total number of children that were evaluated for these outcomes. Why does this differ from the total cohort? What was the loss to follow up? Harmonize reporting for clinical outcomes to clarify (e.g. caregiver reported symptom of infection I n/N, suggest the other values also be reported as n/N

How were caregiver reported symptoms collected? Please address the significant risk of recall bias given the long periods between post-discharge visits (2/4/12/24/48 weeks). How were caregiver symptom reports validated?

Line 86, given the risk of selection bias, please provide supplementary information describing how the selected cases and controls (with blood samples and immune function read outs) relate to the total case numbers for IMMUNO-SAM in terms of demographic and illness characteristics

Figure 1A
- Please provide details on what the the sub-study approval was for in August 2017, given blood sample seems to have been collected prior inclusion.
- Please explain the very high loss to follow up (only 60-69%) of children evaluated at each time point. How was survival assessed with followup of only 68.7% at 48 weeks?

Figure 1B, provide a graphic legend for colors. How does the underlying data for this figure differ from what was presented in the article, Phiri et al, Science Advances, 2023 ?

Figure 2D and Line 146, although 4/5 multivariable linear regressions show no association between PC scores by change in MUAC and 5/5 no associations WHZ, the authors conclude that cases who have a heightened blood immune cell capacity to secrete pro-inflammatory cytokines have a fuller nutritional recovery post-discharge. The reviewer would make the opposite conclusion, namely that the preponderance of evidence supports no association between baseline PC immune scores and growth outcomes. Could the authors address this discrepancy?

The second issue is that the PC axes have questionable biologic or interpretable meaning, not necessarily reflecting 'capacity to secrete pro-inflammatory cytokines', they just reflect a variance in predictors. Can authors address this?

Can the authors conduct sensitivity analyses for the monocyte differences at baseline? Are there linear associations with

anthropometric scores at baseline and at follow up? How do changes in immune phenotypes associate with ponderal growth changes, rather than simply SAM status at baseline?

Across analyses, the authors should clarify if changes in expression or secretion is relative to baseline and by SAM/anthropemtric status over for each immune analysis.
- Line 184 in cases only?
- Line 189, please clarify, were proinflammatory cytokines higher in cases as compared to controls at 48 weeks?

Line 261, age and time post discharge are colinear, how did the authors account for this?
Figure 5, again the biologic relevance of the PC axes is unclear to the reviewer, could the authors expand on how they derived PC2 to be 'pro-inflammatory' and PC1 'bacterial binding' scores

Minor comments
How did the authors deal with repeat measurements in their assessments of significance
The abstract is unclearly written for someone who does not go on to read the rest of the text
- It is unclear how the longitudinal cohort is related to cross-sectional cohort,
- It is unclear at which time point the results "upregulation… are dysregulated" refers
- referring to.
- It is unclear what 'inpatient immune function' and 'hospitalisation' refers to as relates to the two cohorts
Line 43, anti-bacterial innate immune function may contribute; suggest to use 'may associate' as directionality of association not shown by preceding data
Table 1: Nutritional status, Baseline MUAC missing parentheses before 11.2
Supplementary figure 5 should have an F in legend
Figure 4, Supplementary figure 6/7/8 font is very small/ineligible on figures, suggest to increase font size
Line 208, is the reference to Supplementary Figure 8 correct?

Reviewer #3

(Remarks to the Author)
This manuscript addresses an important and understudied area - the relationship between immune recovery and nutritional rehabilitation among children hospitalized with complicated severe acute malnutrition (SAM). The authors hypothesize that immune restoration lags behind nutritional recovery and explore whether there is an association between the rehabilitation of innate immune function and anthropometric recovery up to 48 weeks post-discharge. The longitudinal design, incorporation of immune phenotyping, and long-term follow-up make this work relevant to understanding post-SAM vulnerability. However, several issues require clarification and reorganisation to strengthen the manuscript and improve interpretability.
Major Comments
1. Study Design and Clarity of Cohort Flow
• The description of the study design needs clearer articulation. It appears to be a prospective cohort study with hospitalised SAM children as the exposed group and community children as the comparison (unexposed) group. However, in several places, the text refers to 'cases' and 'controls', which can be confusing as this terminology implies a case–control design.
• The manuscript would benefit from a clear schematic (or an improved version of Figure 1A) showing participant flow, inclusion/exclusion criteria, follow-up visits, and numbers at each stage. The current flow diagram contains inconsistencies in the numbers, which should be cross-checked and corrected.
• Please clarify whether the control group comprised hospitalised or community children. If hospitalised, specify the indication for admission and confirm that they were free of acute infection, as this affects comparability.
2. Presentation of Baseline and Follow-up Data
• Table 1 should typically present baseline characteristics of the study population at enrolment. The current version includes follow-up information, which could be moved to a separate table or supplementary material.
• The authors should ensure that comparisons in the text (e.g., Lines 105–106) specify the reference group-whether comparisons are being made between SAM children with vs. without adverse outcomes, or between SAM children and healthy controls.
3. Causal interpretation and phrasing
• The manuscript occasionally implies causality (e.g., "immune restoration lags behind nutritional recovery" or "deficient innate immunity may underlie persistent morbidity") although this is an observational study with limited capacity for causal inference. The text should more consistently use cautious, associative language (e.g., "was associated with" rather than "led to").
• In the Abstract and Discussion, consider clarifying that the data are consistent with, but do not establish, a causal relationship between immune recovery and nutritional rehabilitation.
4. Integration of Previously Published Data
• Sections of the Results (e.g., Lines 130–131) intermingle findings from the current analysis with results already reported from the HOPE-SAM study. These should be clearly separated: previously published findings should be referenced succinctly in the Introduction or Discussion to provide context, while the Results should focus only on new analyses presented here.
5. Adjustment for multiple testing
• Numerous regression models are fitted (e.g., Supplementary Tables 8–15). Please clarify whether any correction for multiple comparisons was applied. If not, discuss the potential for type I error inflation, particularly in exploratory analyses.
6. Organisation of the Results Section

• Lines 153–160 include material that belongs in the Methods section, describing analytical approaches rather than findings.
• Similarly, Lines 175–177 include interpretative statements that would be more appropriately placed in the Discussion. The Results should remain descriptive and focused on presenting data without interpretation.
7. Quantification of Measures
• Clarify (Line 188) whether the measure being described is quantitative, categorical, or derived from a composite index. If it is a derived measure (e.g., immune restoration score), specify how it was calculated and validated.
8. Discussion
• The Discussion would benefit from a clear opening paragraph summarising the principal findings of this study before interpreting them in the context of prior evidence. Currently, context and background at the start of the Discussion slow the presentation of key results—reverse this order for improved readability.
• Interpretation appears interspersed throughout; restructuring it to flow from summary → interpretation → implications → limitations → conclusions would improve readability.
9. Selection bias and representativeness
• As acknowledged (lines 407–411), children able to provide ≥2 mL of blood likely represent a healthier subset of the SAM population. The discussion could elaborate further on how this might bias findings (e.g., underestimating the magnitude of immune dysfunction or the association with adverse outcomes).
10. Clinical translation
• The conclusion suggests that delayed immune recovery may warrant adjunctive interventions to prevent infection. This is a strong and important implication but would benefit from referencing potential candidate interventions (e.g., anti-inflammatory or microbiota-directed approaches) or current evidence gaps. Otherwise, the recommendation seems somewhat speculative.
11. Timing of early follow-up
• The 12-week gap between discharge and the next immune assessment is a major limitation given that most adverse events occur in this window. It would be helpful to explicitly note this as the period where immunological and clinical trajectories may diverge most, reinforcing the need for finer-grained sampling in future studies.
12. Statistical power and missingness
• The low event rates (e.g., 14 deaths, 22 readmissions) limit the ability to detect associations between immune parameters and clinical outcomes. The authors might quantify power (post hoc) or provide confidence intervals for null findings to emphasize interpretive caution.
Minor Comments
1. Terminology
• Use consistent terminology for ‘complicated SAM’ vs. ‘hospitalised with SAM’ throughout. Occasionally both terms are interchanged without definition'.
• Replace ‘nutritionally cured’ (p.4) with ‘met discharge nutritional criteria’ or ‘achieved nutritional recovery’ to avoid ambiguity.
2. Methods
• Clarify the timing of the ‘baseline’ sample (admission day vs. during hospitalization). It seems to vary (lines 520–522).
• Provide justification for using PCA-derived components rather than mixed models on individual immune variables—particularly since interpretation of PCs over time may shift.
3. Figures
• Figures 3–6 are data-rich but crowded. Adding inset legends explaining what positive/negative changes mean biologically (e.g., ↑PC1 = higher bacterial binding) would improve accessibility for non-specialist readers.
• Figure 2D could include correlation coefficients or trend lines to visualize the strength of associations with ΔMUAC.
4. Discussion
• The discussion effectively integrates murine evidence but could more clearly distinguish which mechanisms are speculative versus supported by human data.
• The statement that “bacterial binding capacity remained abnormal in recovered children” (p.17, line 347) could be tempered by acknowledging that the differences, while statistically significant, may or may not be clinically meaningful.
5. Others
• Line 80: Clarify the characteristics of the control group — were they hospitalised, or community-based?
• Line 84: Replace ‘cases and controls’ with ‘SAM and comparison groups’ or equivalent terminology consistent with a cohort study design.
• Line 146: Rephrase to avoid switching between ‘our cohort’ and ‘cases’ in the same sentence. Suggestion: “Our cohort data indicated that hospitalised children with SAM…”
• Line 353: Typographical error — replace where with were.
• Figure 1A: Verify participant numbers for consistency throughout.
• Please include the statistical comparison methods used for between-group analyses in the Methods (e.g., regression models, mixed models, or non-parametric tests).
Overall Assessment
This is a valuable and well-conceived study that makes a meaningful contribution to understanding the immunological recovery trajectory following SAM. The analysis is novel, but the manuscript would benefit substantially from clearer description of the design, improved data presentation, and stricter separation of Methods, Results, and Discussion. Addressing these points will improve study clarity, strengthen the reporting, and support the impact and reproducibility of your findings.

Version 1:

Reviewer comments:

Reviewer #1

(Remarks to the Author)
Comments were addressed adequately.

Minor revision:
Figure 3 panels B and C remain too small to read, even with significant magnification.

Reviewer #4

(Remarks to the Author)
I have been asked to review this manuscript following a round of revisions as one of the original reviewers was unable to follow-up their review.

This manuscript describes work that is novel, important, and methodologically sound. The noteworthy results and importance to the field have been outlined by the prior reviewers, and I concur. The manuscript is well written, the interpretation and conclusions are appropriately phrased and are in line with the data presented. The manuscript has been improved by the round of revisions, and I consider that all the prior reviewers' comments have been appropriately addressed. There are no flaws in the data analysis or other aspects of the manuscript that would require a further round of revisions. Data and methodology are provided adequate to support reproduction.

I am in favour of the manuscript now moving forward to publication without further delay.

Reviewer #5

(Remarks to the Author)
I was asked to examine author responses to Reviewer 2 and find that the authors have satisfactorily addressed most of the issues raised by Reviewer 2 in an unbiased and transparent way.

Open Access This Peer Review File is licensed under a Creative Commons Attribution 4.0 International License, which permits use, sharing, adaptation, distribution and reproduction in any medium or format, as long as you give appropriate credit to the original author(s) and the source, provide a link to the Creative Commons license, and indicate if changes were made.
In cases where reviewers are anonymous, credit should be given to 'Anonymous Referee' and the source.
The images or other third party material in this Peer Review File are included in the article’s Creative Commons license, unless indicated otherwise in a credit line to the material. If material is not included in the article’s Creative Commons license and your intended use is not permitted by statutory regulation or exceeds the permitted use, you will need to obtain permission directly from the copyright holder.
To view a copy of this license, visit https://creativecommons.org/licenses/by/4.0/

RESPONSES TO REVIEWER COMMENTS

Thank you to all 3 Reviewers for their time and consideration of our manuscript. Your comments are reproduced in full below (black text) and our responses to each are provided below the relevant comment (red text). We indicate where a comment has been addressed or our response to your comment has been modified in response to another Reviewer.

Reviewer #1 (Remarks to the Author):

● What are the noteworthy results?
The noteworthy results of this paper are that children hospitalized with acute SAM have increased innate immune cell sensitivity to bacterial binding, decreased pro-inflammatory cytokine response, and impaired monocyte marker expression as compared to healthy controls from their communities. Over the course of 48 weeks of followup, this dysregulated profile generally approached a more recovered profile more similar to the controls. However, even after nutritional recovery, several metrics of innate immune function remained distinct in cases vs. controls, suggesting that full immune recovery takes longer than nutritional recovery. A stronger pro-inflammatory cytokine response was associated with better nutritional recovery, but variation in innate immune function in SAM patients was not associated with adverse clinical outcomes.
● Will the work be of significance to the field and related fields? How does it compare to the established literature? If the work is not original, please provide relevant references.
Yes, this work highlights the extended vulnerability of children with complicated SAM to infection beyond their nutritional recovery, which has direct clinical implications. While SAM-associated vulnerability to infection has long been recognized, this study provides more specific mechanistic insight into SAM-associated immune system changes, and their connections to clinical outcomes. This study also explores the question of whether adverse clinical outcomes in a subset of SAM patients are associated with differences in innate immune function, finding that they were not.
● Does the work support the conclusions and claims, or is additional evidence needed?
The work does support the conclusions and claims.
● Are there any flaws in the data analysis, interpretation and conclusions? Do these prohibit publication or require revision?
No apparent flaws in data analysis. A more thorough interpretation of the differences observed in bacterial binding sensitivity, and a consideration/refuation of potential seasonal effects would strengthen the interpretation/limitations section.
● Is the methodology sound? Does the work meet the expected standards in your field?
The methodology appears sound.

● Is there enough detail provided in the methods for the work to be reproduced?
Yes

We appreciate your considered review and address your key concerns around interpretation of our results and the role of seasonality in the revised version; we outline how we have responded to each of your specific comments below.

Major Comments:
Line 7: Describe and explicitly define "complicated SAM" and the common clinical complications associated with SAM for the purposes of being included in this cohort. While many complications are mentioned, it is unclear whether these fall under the category of "complicated SAM" for the purposes of this study. Perhaps these complications could also be listed in Table 1.

We have now added the following statement to clarify on this point: "*Complicated SAM is a heterogeneous condition with complications requiring hospitalisation defined as Integrated Management of Childhood Illness clinical danger signs, acute infection, severe oedema, or feeding problems*[1]."

We have previously reported all complications present in our cohort and have now signposted to this information in the Table 1 legend so that readers can access this more readily: "[3]*Individual clinical complications present alongside SAM at hospital admission in this cohort are reported elsewhere*[21]."

Line 27-28: Clarify meaning of phrase: "implicate a role for the immune system in SAM pathogenesis." Is the intended meaning that the immune system might play a causal role in SAM? Or is the intended meaning that SAM-induced immune system changes play a role in SAM-related mortality and morbidity? Current wording - esp. "pathogenesis" implies the former, but it seems like the latter fits more with the findings of the study.

We have now replaced '*pathogenesis*' with '*mortality*' for clarity in the context of this statement.

Introduction, Results, Discussion: When discussing findings of this study and related studies, reconsider repeated use of vague nouns like " shifts in anti-bacterial innate immune cell function" (Line 43), or "sustained differences in anti-bacterial innate immune cell function" (Line 436-437). When possible, specify the specific changes referenced – perhaps increased sensitivity to bacterial binding, or decreased pro-inflammatory cytokine response, or impaired monocyte marker expression. While the instinct to use words like "shifts", "dysregulated", or "differences" is understandable given the range of changes observed, adding brief elements of specificity when appropriate would contribute to improved clarity and usefulness to readers.

Thank you for this suggestion. We agree that specificity is important and have revisited the manuscript to add more specificity were referring to individual outcome measures. For example:

"These data suggest that children with SAM have a distinct combination of subsets within the circulating monocyte pool, coinciding with lower abundance of total monocytes than that of healthy controls up to 48 weeks post-discharge."

"We have previously observed that cases within the IMMUNO-SAM cross-sectional cohort have higher basal bacterial binding capacity than controls but lower monocyte activation and pro-inflammatory cytokine responses to bacterial antigens in vitro[21]…"

However, where a result reflects a composite outcome measures (Principal Components) and reciprocal changes (e.g. increase in PC2 scores relative to decrease in PC1 scores over time), we have retained the more general terminology alongside providing full details of the individual outcome measures loaded onto each PC in Figure 1B. We hope that this approach satisfies your comment.

Similarly, outlining the "dysregulated" immune status vs. the "recovered" immune status with more clarity would serve a similar purpose. In Results section headings and figure titles, a few more directional words to clarify "normalizes" would also be helpful.

We have now added a statement to the first results section reporting on longitudinal changes in immune function to clarify this and put our reference to 'recovered'/'normalises' into context:

"In our longitudinal mixed effects linear regression of immune function variables by visit and case/control status, we refer to 'immune restoration' where cases had distinct scores for an immune function variable compared to controls at baseline, but these scores subsequently converged to be within control ranges during post-discharge follow-up. 'Normalisation' was indicated by scores becoming more like those of the control group, even they it remained significantly different at post-discharge visits."

We also include mean and confidence intervals for healthy controls at baseline on all spaghetti plots and coefficient values in all tables to provide transparency on actual change relative to baseline/controls (unchanged from original version of manuscript).

Discussion: While the altered intermediate/classical/non-classical ratios of monocytes were well-explained and a possible mechanism was provided, such an explanation for the observed increase in bacterial binding capacity was not provided in the discussion. Any insights into the potential mechanisms or implications of this change, especially if any new ideas have been generated since the previous work showing this finding, would be a valuable addition to the paper.

Thank you for this comment and we agree that further discussion of the patterns seen in individual functional traits are warranted. Ours is the first study to evaluate bacterial binding capacity in SAM and there is therefore a very limited literature to make evidence-based speculation on the underlying mechanism. However, some recent murine studies and a small study in human sepsis may be pertinent; we have now added some additional discussion to address your suggestion. We have also added a new publication from James Njunge's research team in Kenya on associations between immune mediators and longer term post-discharge mortality among children recovering from complicated SAM, published last week.

Line 357-369: This crucial insight would be better served at the conclusion of the Discussion section. In its current placement at the end of a paragraph about bacterial binding capacity, it seems out of place and its impact is lessened.

Thank you for this suggestion. We have now significantly rephrased the first and last paragraphs of the discussion so that our conclusion is more impactful.

Limitations: Consider the potential influence of seasonality on the immune metrics measured in this study, and whether they were accounted for in study enrollment. Seasonal variations in innate immune response have been observed in other populations. The collection of a singular control blood sample at baseline leaves open the possibility that some fraction of changes attributed to recovery in this study could also be influenced by seasonality, unless the timing of collection varied enough to preclude this.

Although controls and cases were recruited across the calendar year throughout the study, it is true that resource and ethical constraints precluded collecting longitudinal samples from healthy controls. We have therefore added the following statement and supporting references to the Limitations section (note: age is also included in response to Reviewer 2):

"Although both the SAM and healthy control groups were recruited across a range of ages and seasons, reducing the likelihood that observed differences were driven by systematic age or seasonal bias, we recognise that a potential small effect of age and seasonal variations on healthy immune function cannot be fully excluded[50, 51]."

Figure 4: This figure has too many panels and is hard to read. Panels J and N overlap with the adjacent panels, making them look messy. Consider pruning this group to just LPS or just HKST, or else reducing to some other representative group and including the full group in supplemental figures.

Response to Reviewer comments

We apologise for the small text size and overlapping panels in the in-line version of our manuscript; this was an artefact of compression into the combined pdf. To simplify this figure and improve legibility, we have separated it into 2 figures presenting data on 1) monocyte activation (new Figure 4) and 2) cytokine responses to bacterial antigens (new Figure 5). Figure numbering has been updated throughout the text accordingly.

Figure 5: Reconsider overlap with Figures 3, 4, and 6 in main figures. Figure 5 has good parallelism with the corresponding section in Results, but only the first two panels are relevant to the narrative function of this figure as it is currently presented. The information in this figure's title also seems to be already provided in figures 3 and 4. It is also remarkably similar in structure to Figure 6 – is it possible to combine them in some way (like using color coding of "recovered" children + listing of respective statistics in a table format)? Given the abundance of figures in the supplement, and the relative importance and explanatory power of some of them (especially supplementary figure 10), perhaps the space is better given to another figure.

Thank you for this comment; we appreciate that there is some overlap between these figures. However, we have respectfully chosen to retain these Figures as we believe they present our data with greatest transparency: Figure 3 and 4 (Figure 4 now split into new Figures 4 and 5) to present novel patterns of individual immune function analytes, for which there are no existing data in the field; Figure 5 and Figure 6 (now Figures 6 and 7 in the revised version) address 2 separate hypotheses and associated analyses, which is why we have retained them as stand-alone figures.

We agree that Supplementary Figures 10 and 11 have explanatory power; they present the same analyses as indicated in Figure 5 and Figure 6 (now Figures 6 and 7 in the revised version) but as the summary predicted model values rather than the raw individualised data per participant. We recognise that some readers may prefer the former presentation over the latter and vice versa, so provide both for completeness.

Minor Comments:

Line 3-6: Adjust wording/rearrange clauses to improve readability and reduce nested parentheticals. "Affecting nearly twenty million children globally, severe acute malnutrition (SAM) is a form of undernutrition identified by severe wasting, nutritional oedema, or both. Clinically, severe wasting is defined by weight-for-height Z-scores (WHZ) <-3 and/or mid-upper arm circumference (MUAC) <115mm.

Thank you. We have now rearranged this statement as suggested.

Line 191-193: change wording to clarify meaning and add period. Suggest changing to "Thus, dysregulated circulating monocyte phenotypes and pro-inflammatory cytokine secretion in cases at hospital admission appear to normalise with time post-discharge.

We have rearranged this statement as suggested.

Line 371-375: “Total monocytes…” sentence is hard to follow. Consider splitting into two sentences. Phrase “that decreased and increased respectively during follow-up” appears to be a fragment – consider removing “that”

We have rearranged this statement as suggested.

Line 403: Missed word, add “children recovering from SAM”

We could not spot the missing word as ‘children recovering from SAM’ is already part of this sentence but please signpost us if we have missed this in the revised version.

Line 436 - 438: Sentence is hard to follow; recommend changing to make the subject more clear e.g. “In children hospitalized with complicated SAM, we found sustained differences in anti-bacterial innate immune cell function over a 48-week post-discharge follow-up, as compared with healthy controls”

We have rephrased this paragraph in response to your earlier suggestion about impactful conclusions.

Line 982: Extra “innate” in title

We have removed the additional ‘innate’ here and where it was also duplicated in the title of other supplementary figures.

Reviewer #2 (Remarks to the Author):

In the manuscript, “Restoration of anti-bacterial innate immune function lags behind nutritional recovery among children convalescing from complicated severe acute malnutrition” authors Rukobo et al study a disease of global importance, namely severe acute malnutrition which is associated with considerable post-discharge/recovery mortality. The manuscript uses a cross sectional and longitudinal study design to assess changes in anti-bacterial innate immune function in children with SAM relative to their clinical outcomes and subsequent ponderal growth changes. The study subject is important as it provides insights into what immune vulnerabilities children with SAM may have and may keep in their recovery trajectory. The authors find that bacterial binding capacity decrease and pro-inflammatory cytokines increase with normalization of growth, but that these disruption persists despite recovery from SAM.

General comments:

In this manuscript the authors are extending findings they made in a previous publication (Phiri TN, et al. Severe acute malnutrition promotes bacterial binding over proinflammatory cytokine secretion by circulating innate immune cells. Sci Adv, 2023) where they found that clinically admitted children with SAM had high bacterial binding capacity that associated with persistent SAM at time of hospital discharge. They now report on follow-up and testing of a subset of these children, assessing associations with clinical outcomes, ponderal growth over time and in comparison to a community control group. In this way, the study provides incremental gains but do deepen understanding of earlier reported findings.

We appreciate your thoughtful evaluation of our manuscript and that you consider that it will deepen understanding within the field. We recognise the constructive criticism offered and outline how we have addressed each of your concerns below.

The major questions/weaknesses for the manuscript are on the study design and implementation, and statistical analyses. In their first analyses (Line 102-3), the authors assess whether inpatient immune function differs between children with and without adverse clinical outcomes post-discharge. However, methodology for collecting clinical events data during follow up is of low quality, precluding meaningful assessment of these outcomes. For example, recall is used with study visits spaced more than 12 and 20 week intervals, making it highly unlikely that there is accurate capture of clinical events.

We agree that infectious symptom data post-discharge reported in Table 1 was predominantly reliant on caregiver recall, which is not the highest quality method. We report details of symptoms solely for completeness/transparency in Table 1/text and as supplementary information, but our inference is based on our more robust clinical outcome data (clinic visits, hospital readmissions, nutritional relapse and mortality; Figure 1), which were verified by direct measurement (nutritional status at 48 week visit), from caregivers' hand-held child health cards (clinic visits and readmissions) and with a dedicated case report form (mortality).

We have retained reporting of caregiver-reported symptoms so that all available data is presented in the manuscript.

In addition, there is no differentiation in the analyses between infections/possibly infections and non-infectious clinical events, which would be relevant to these immune outcomes.

Thank you for the comment; we agree that we could have been clearer that our analyses do differentiate between the two types of event. To sign- post our approach more openly we have duplicated the information under “Clinical outcomes” in the methods section

that, though the reasons for the clinic visit and hospitalisation were not always known, all were confirmed to be outside the routine clinic visit schedule (included in the methods section). Research nurses made every effort to review written information in child health booklets, which are carried by the caregiver and used by clinic staff to document consultations. Furthermore, hospital admissions solely for pre-existing congenital or developmental conditions (e.g. congenital heart disease) or due to traumatic injury (e.g. burns, fractures) were not included, as indicated in Table 1 legend. Full details of all-cause re-admissions have previously been reported (reference #2, Bwakura-Dangarembizi et al, 2022).

In addition, the study has very poor follow up (only 60-70%) with minimal criteria (> 1 follow-up visit) for inclusion in the longitudinal cohort. These major methodologic limitations preclude any meaningful interpretation of the clinical outcomes data as reported in Figure 2). These same issues should be addressed for the immune assessments in the longitudinal cohort, where follow-up was similarly poor for immune analyses. How did the authors deal with these issues in their analyses?

We agree that a higher percentage follow-up rate would have been ideal. However, given the recognised challenges of following up critically ill children after hospital discharge in low income settings and within a very disadvantaged caregiver group among whom mobility is very high, our follow up rate is higher than most previously reported longitudinal studies among children affected by complicated SAM (e.g. high loss to follow-up in all studies included in a 2021 systematic review of mortality outcomes for studies among children discharged after hospital admission for SAM; Noble et al, Am J Clin Nut, 2021). We acknowledge this as a limitation in the 'Limitations' section of the discussion as follows:

"As is typical for hospital-based longitudinal follow-up studies of children recovering from SAM[4, 48], not all cases provided a sample at all visits, and some were lost to follow-up. Such missing data is unlikely to be at random and this bias likely reflects recognised socio-economic challenges faced by affected families, as reported by caregivers of HOPE-SAM participants[49]. Our results are therefore most generalisable to easier to reach children who are likely less affected by barriers to accessing healthcare."

To address the missingness in our longitudinal data, we use a mixed model approach, including participant identifier as a random effect. Such models use Maximum Likelihood to analyse all available data, not only participants with complete data, and thus help mitigate bias due to loss of participants across timepoints, which we agree are unlikely to be missing completely at random. While imperfect, this represents a pragmatic approach for our valuable dataset, moreover, the assumption of missing at random (MAR) is supported by the variables included in the model; accordingly, although some statistical power is lost due to incomplete data, this should not

introduce substantial bias. To address this concern directly, we had already included the following statement in the 'Limitations' section of the Discussion:

"Whilst inclusion of relevant confounders as fixed effects and individual participant as a random effect in our mixed effects models go some way to addressing the influence of inter-individual heterogeneity within our cohort, a larger sample size with less missingness would have given greater power to detect longitudinal patterns of immune function and explore previously identified clinical risk groups in more depth[2, 3, 13]."

We have now also added the following to the 'Statistical methods' section in the Methods to further highlight our approach:

"Such models use Maximum Likelihood to analyse all available data from cases rather than only participants with complete follow-up, thereby reducing potential bias from missing data at one or more visits."

Another major weakness in the study design is that there were no follow ups for the control arm children, complicating comparisons with cases by agen and over time. This is important as there are likely age-dependent changes in immune function in healthy children. Could the authors assess age within the control group and whether there are any alterations in immune function by age that would be confounding results over time in the case group.

Although controls and cases were recruited across a range of ages, it is true that resource and ethical constraints precluded collecting longitudinal samples from healthy controls. We have therefore added the following statement and supporting references to the Limitations section (note: seasonality is also included in response to Reviewer 1):

"Although both the SAM and healthy control groups were recruited across a range of ages and seasons, reducing the likelihood that observed differences were driven by systematic age or seasonal bias, we recognise that a potential small effect of age and seasonal variations on healthy immune function cannot be fully excluded[50, 51]."

Our longitudinal cohort is not powered to analyse the specific effects of age group however we fully agree that age can be an influential factor for immune function and found that this was the case for SAM inpatients during hospitalisation with PC4 (myeloperoxidase release) (Phiri et al, Sci Adv, 2023). To adjust for the potential confounding effect of age on longitudinal patterns of immune function in cases and controls, age group at enrolment is included in all adjusted linear regression and mixed effects regression models and we report both the unadjusted and adjusted (for baseline age group and other confounders) in all relevant supplementary results tables. We have now specified 'at enrolment' at all instances where we describe adjustment of a model

by age group.

In their analyses, the authors employ PCA and use variation on the PC axes to further assess changes over time. The biologic relevance of these axes is sometimes questionable. The authors could better address and/or explain this.

Thank you for this comment. We agree that we could have explained our approach better, particularly since the absence of studies evaluating the relationship between immune function outcomes and clinical outcomes among children with SAM means that our PC scores are not directly translatable to commonly used clinical/biologic scales. To address this more clearly in the main text, we have removed an early reference to the PCA without the full explanation and expanded on how the PCA was conducted in the main text reporting of data in Figure 1 as follows:

"An effective cellular immune response to bacterial challenge reflects a balance of different innate immune cell functions, as has been reported for monocyte/macrophage responses in human sepsis[32, 33, 34]. We have previously reported Principal Component Analysis (PCA) of all individual anti-bacterial innate immune function read-outs from whole blood culture and bacterial binding assays for IMMUNO-SAM cases during their hospitalisation and controls at baseline, identifying 5 key domains of combined innate anti-bacterial immune function (Principal Components (PC); factor loadings for all individual variables included in the PCA are shown in ***Figure 1B****)[21]. These composite variables have the advantage of reducing the number of variables for statistical analyses and better reflect the interacting nature of anti-bacterial immune responses in vivo than evaluating individual immune functions one at a time. PC scores correspond to patterns of the immune function variables with which they are loaded (e.g. PC2 is positively loaded with tumour necrosis factor (TNF)α, interleukin (IL)-6 and IL-8) in response to bacterial antigens (LPS and HKST), therefore higher scores for PC2 reflect higher pro-inflammatory cytokine responses to bacterial antigens)."*

And rephrased our reporting of the data in Figure 5 (now Figure 6 in the revised version) as follows:

*"We hypothesised that these factors may also influence post-discharge patterns of rehabilitation in the 5 key domains of anti-bacterial innate immune function identified by PCA of cross-sectional immune function data (****Figure 1B****). We therefore applied the same PCA approach to generate scores for PC1-5 to longitudinal immune function at discharge, 12, 24 and 48 weeks post discharge using post-estimation."*

Finally, it would be helpful if the authors clarified some of their hypotheses. There are two related but ultimately separate hypotheses in the paper – first that immune dysfunction could help explain post discharge clinical outcomes, second, that immune dysfunction observed at the time of admission for SAM may persist despite nutritional

recovery. These are not the same pathophysiologically or mechanistically. Suggest that the authors clarify the two in text.

We agree that we are addressing multiple hypotheses which are separate and likely driven by distinct mechanisms. We have rephrased the last paragraph of the introduction to frame our aim and the 3 key hypotheses we are addressing more clearly.

"In this study we explored whether rehabilitation of anti-bacterial innate immune cell function ('immune restoration') is associated with anthropometric recovery ('nutritional recovery') using longitudinal blood samples collected from our existing IMMUNO-SAM cohort[21]. We tested 3 key hypotheses: 1) immune function during hospitalisation is associated with subsequent adverse clinical outcomes and/or ponderal growth gains post-discharge (ΔMUAC and ΔWHZ); 2) anti-bacterial innate immune cell function changes at discharge, 12, 24 and 48 weeks relative to their baseline inpatient immune function and compared to heathy controls; and, 3) anti-bacterial immune function of children who have made a full nutritional recovery from SAM continues to differ from that of healthy controls."

We have also clarified which hypothesis we are addressing in the relevant sections of the Results.

Specific comments:

Table 1

There are significant differences between the community controls and the SAM inpatients, most importantly, age and HIV status which could cause significant confounding in their results. Did the authors consider age matching cases to controls.

IMMUNO-SAM is not a matched study but was nested within a wider observational cohort study for which the primary outcome measure was post-discharge mortality at 48 weeks (HOPE-SAM study previously published and cited in text). We conducted the immunology analyses on every sample/participant from the parent study that met our inclusion criteria to maximise sample size. To emphasise this, we have added the following statement to the Clinical study design section of the methods:

*"Cases and controls were not matched for the IMMUNO-SAM study; statistical analyses included adjustment for characteristics that differed between the two groups at baseline (i.e. age group at enrolment and HIV status; **Table 1**). Selection of the IMMUNO-SAM cohort is summarised in **Figure 1A**. All eligible participants were included in statistical analyses to maximise sample size."*

To reflect the potential confounding effects of differences in age and HIV status between cases and controls at baseline, we include these variables alongside other relevant confounders in all adjusted linear regression models. We report this in the relevant figure legends and in the statistical methods and have been transparent about

the characteristics of case and control groups in Table 1. To further address your comment, we have added the following statements to the Clinical study design and Statistical analyses sections of the methods:

*"Fixed effects were selected for inclusion in adjusted models to reflect imbalance between cases and controls at baseline (age group at enrolment and HIV status; **Table 1**) and variables that we have previously been found to influence immune function (age group at enrolment, HIV status[21]) and/or clinical outcomes (sex, age group at enrolment, HIV status, baseline oedema, cerebral palsy, country and baseline WHZ[2, 3, 13, 36]) in children with SAM."*

What was the rationale for combining (line 946) the < 6 months and 6-11 month age groups?

Thank you for this question. We combined the 2 groups as there were only n=7 participants in the <6mo age group for cases; we used the same groupings in our previous published work on cross-sectional inpatient immune function. We have now added these details to the Table 1 legend as follows:

"...the <6mo (cases n=7; controls n=0) and 6-11 (cases n=16; controls n=10) month age groups were combined for IMMUNO-SAM analyses."

This sentence is confusing: "number of re-admissions among re-admitted children"

We have rephrased the table to address the confusion.

The N's for each of the clinical outcomes is also unclear. For example clinic visit for reasons outside normal schedule, is n the population that had an extra clinic visit, or the total number of children that were evaluated for these outcomes. Why does this differ from the total cohort? What was the loss to follow up? Harmonize reporting for clinical outcomes to clarify (e.g. caregiver reported symptom of infection I n/N, suggest the other values also be reported as n/N

Thank you for this opportunity to clarify. As is typical for longitudinal clinical studies with multiple outcome measures, some longitudinal variables (including non-primary clinical variables) do not have complete data for follow-up (e.g. where vital status data was collected but a participant's caregiver declined to complete the full study visit), which is why the N differs by variable. We have opted to report all data available rather than limit reporting to only participants with complete data on all variables to minimise bias. Where the N for a variable differs from the total cohort, the difference is the loss to follow up for this variable. Figure 1A summarises cohort selection/loss to follow-up relative to the parent study (HOPE-SAM).

To address your comment more directly, we have added more details of how the sub-study data collection relates to the parent study (supporting details in the materials and methods section and Figure 1A flow diagram) to the first section of the Results. We have also added the following to the Table 1 legend to clarify:

“[1]Total number of children included in the IMMUNO-SAM cohort with complete immune function data. Where data on a specific variable is missing, n refers to the number of participants positive for that variable and N to the total number of participants with available data for that variable in controls, cases.”

How were caregiver reported symptoms collected? Please address the significant risk of recall bias given the long periods between post-discharge visits (2/4/12/24/48 weeks). How were caregiver symptom reports validated?

We have provided additional details in the methods section to supplement the information in the Table legend, as follows:

“Caregiver reported symptoms, hospital admissions, and clinic visits solely for pre-existing congenital or developmental conditions (e.g. congenital heart disease) or due to traumatic injury not plausibly influenced by preceding immunological differences (e.g. burns, fractures) were excluded from analyses for the IMMUNO-SAM study. Full details of all-cause re-admissions have been previously reported[2].”

Line 86, given the risk of selection bias, please provide supplementary information describing how the selected cases and controls (with blood samples and immune function read outs) relate to the total case numbers for IMMUNO-SAM in terms of demographic and illness characteristics

To address your comment, we have added a paragraph to the Results section providing an overview of clinical outcomes previously published for the parent clinical study (HOPE-SAM) and signposting to the relevant HOPE-SAM publications for full details:

“Full details of clinical outcomes from the full HOPE-SAM cohort (n=745 cases) have been previously reported[2, 3]. Briefly, mortality among cases was 9.4% during hospitalisation and 9.1% over the post-discharge follow-up period[2, 3]; of the 649 children discharged from hospital, vital status at the end of follow-up was ascertained for 93.1%. Post-discharge morbidity was high with 48.8% of cases having ≥1 caregiver reported symptom by week 4 post-discharge and 15.4% of cases re-admitted to hospital[2]. SAM was still present in 43.9% of cases at discharge, 12.4% at week 12, 11.9% at week 24, and 4.5% by the end of follow-up[2].”

We have previously published a full table comparing the IMMUNO-SAM and HOPE-SAM cases by their demographic characteristics during hospitalisation. We therefore provide a sentence summary and citation as follows:

“Given the requirement for an inpatient blood sample, cases included in the IMMUNO-SAM cohort tended to be healthier at hospital admission (i.e., lower mortality during hospitalisation, slightly older, with less severe wasting, and a lower percentage with

SAM at discharge) than the HOPE-SAM cohort as a whole; a full comparison of clinical and demographic characteristics during the inpatient period has been previously reported[21].”

Figure 1A

- Please provide details on what the the sub-study approval was for in August 2017, given blood sample seems to have been collected prior inclusion.

The approval was for evaluation of immune function assessment within the IMMUNO-SAM sub-study which required real time assays to be conducted on fresh blood samples that had not been cryopreserved. We have added ‘*freshly collected blood’* to the relevant methods sections to clarify this requirement. Blood samples for clinical tests (e.g. HIV testing) were collected throughout HOPE-SAM but these did not have a volume requirement and were not suitable for immune function assays prior to August 2017.

- Please explain the very high loss to follow up (only 60-69%) of children evaluated at each time point. How was survival assessed with followup of only 68.7% at 48 weeks?

Clinical follow up was part of the HOPE-SAM parent study and was not affected by missing or low volume blood sampling for the longitudinal cohort of the IMMUNO-SAM study. We have added the paragraph on published HOPE-SAM clinical outcome to the results section, to provide context on what is already known about survival in the wider cohort in response to your earlier comment.

To specifically address this comment, we have added the follow up rate for vital status within the HOPE-SAM study:

“…of the 649 children discharged from hospital, vital status was ascertained for 93.1%.”

Figure 1B, provide a graphic legend for colors. How does the underlying data for this figure differ from what was presented in the article, Phiri et al, Science Advances, 2023 ?

Thank you for this suggestion. We have now added a graphical legend to support the description of the colour coding in the legend as requested.

Figure 1B reports the same PCA as we have previously reported in Phiri et al, Science Advances, 2023 for the cross-sectional cohort, as indicated in the figure legend and main text. We considered it essential to reproduce this figure as part of Fig 1 for this study as we apply post-estimation from the original PCA to generate scores for the follow-up samples from the longitudinal cohort.

Figure 2D and Line 146, although 4/5 multivariable linear regressions show no association between PC scores by change in MUAC and 5/5 no associations WHZ, the authors conclude that cases who have a heightened blood immune cell capacity to secrete pro-inflammatory cytokines have a fuller nutritional recovery post-discharge. The reviewer would make the opposite conclusion, namely that the preponderance of

evidence supports no association between baseline PC immune scores and growth outcomes. Could the authors address this discrepancy?

We appreciate your comment but respectfully disagree with your interpretation that there is a discrepancy in our data and reported inference. PC2 reflects pro-inflammatory cytokine secretion (strong factor loadings (>=0.3 in PCA) for LPS-specific IL-6, TNFa and IL-8 and HKST-specific IL-6 and TNFa; factor loadings indicated in Figure 1B) and is significantly positively associated with change in MUAC in our multivariable linear regression model (Figure 1D, top row, middle panel). We also demonstrate a significant positive association between HKST-induced TNFα and change in MUAC; shown in Supplementary Figure 3 where we present associations for each of the individual pro-inflammatory cytokine variables loaded onto PC2. We find a similar pattern of associations between PC2 and gains in WHZ, albeit not statistically significant per our reporting (Adj. coefficient: 0.10, p=0.065), and between LPS-specific IL-8 and WHZ gains (Adj. coefficient: 0.07, p=0.030; Supplementary Figure 4).

We agree that we find no significant associations for the other domains of immune function that we assessed (PC1, PC3, PC4 and PC5) and report no association for these functions with growth gains both the figures and main text.

The second issue is that the PC axes have questionable biologic or interpretable meaning, not necessarily reflecting 'capacity to secrete pro-inflammatory cytokines', they just reflect a variance in predictors. Can authors address this?

Thank you for this comment. We agree that we could have explained our approach better and have addressed this in the main text in response to a similar comment from Reviewer# 1 (above). PCA is a factor reduction method and therefore groups of variables with similar variance patterns, as you say. All cytokine variables included in the PCA reflect cytokine response to bacterial antigens after subtraction of background levels without stimulus (i.e. the activated cytokine response) and these are positively loaded onto PC2 from the PCA suggesting that positive scores for PC2 reflect higher levels of the pro-inflammatory cytokine responses. PC2 is a good example where we see a suite of pro-inflammatory cytokines loading positively together, which increases our confidence in the biological interpretation of the component. We therefore stand by our interpretation of PC2 as a composite variable reflecting the capacity to secrete pro-inflammatory cytokines.

We have added the following statement to clarify on how we are interpreting each PC using PC2 as an example, to address your comment directly in the main text:

"PC scores correspond to patterns of the immune function variables with which they are loaded (e.g. PC2 is positively loaded with tumour necrosis factor (TNF)α, interleukin (IL)-6 and IL-8) in response to bacterial antigens (LPS and HKST), therefore higher scores for PC2 reflect higher pro-inflammatory cytokine responses to bacterial antigens)."

Can the authors conduct sensitivity analyses for the monocyte differences at baseline? Are there linear associations with anthropometric scores at baseline and at follow up?

How do changes in immune phenotypes associate with ponderal growth changes, rather than simply SAM status at baseline?

Thank you for this suggestion for a new analysis focused on monocyte phenotype and growth gains to supplement our primary analyses/research questions on monocyte and neutrophil function. We have conducted the suggested analyses, focusing on gains in MUAC where we have the best evidence for associations with anti-bacterial function, and added the following to text to the results section:

"Monocytes are a major source of pro-inflammatory cytokines in response to bacterial antigens in the blood and differentiate along a continuum from the classical (CD14+CD16-) to intermediate (CD14+CD16+) to non-classical (CD14^{lo}CD16+) phenotypes with distinct capacities to respond to pathogen antigens[35]. We therefore undertook supplementary unadjusted linear regression of the relationship between proportions of total monocytes (Lineage- (i.e. CD3-CD19-CD20-CD56-) CD66b- HLA-DR+) within all blood leukocytes and monocyte subsets within the total monocyte population and gains in MUAC from baseline to 48 weeks among the cases. We did not find evidence to support a significant association between log-transformed proportions of total monocytes (Coef: 53.72, 95%CI: -191.09, 298.52, p=0.664) or monocyte subsets (classical Coef: 25.32, 95%CI: -10.63, 61.28, p=0.165; intermediate Coef: -31.15, 95%CI: -99.20, 36.90, p=0.365; non-classical Coef: -17.04, 95%CI: -57.21, 23.13, p=0.401) and gains in MUAC. Collectively, our data indicate that cases who have a heightened blood immune cell capacity to secrete pro-inflammatory cytokines in response to bacterial antigens during hospitalisation may go on to have a fuller nutritional recovery post-discharge. This pattern was not solely due to differences in monocyte composition."

Across analyses, the authors should clarify if changes in expression or secretion is relative to baseline and by SAM/anthropemtric status over for each immune analysis.
- Line 184 in cases only?

Thank you for this opportunity to clarify. We only collected longitudinal samples from cases; therefore, all temporal comparisons are for cases only. We have added "*in the cases*" to this line for clarity and emphasised that controls were only sampled at a single timepoint (baseline) as follows in the opening paragraph of the Results section as follows:

*"A group of adequately nourished non-hospitalised children who were not acutely unwell and had a known HIV status were recruited from the same sites for all HOPE-SAM sub-studies and evaluated at a single timepoint (baseline); those who provided ≥2mL blood samples were included as healthy controls for the IMMUNO-SAM study (controls, n=73; **Figure 1A**)."*

- Line 189, please clarify, were proinflammatory cytokines higher in cases as compared to controls at 48 weeks?

In this section, we already state "*within the cases*" for the temporal comparisons and hope that the additional text added to emphasise sampling from controls at a single timepoint in response to your comment above helps to clarify the comparisons for this section.

Line 261, age and time post discharge are colinear, how did the authors account for this?
In the multivariable mixed regression models, we include age group at enrolment as a fixed effect to adjust for confounding effect of age at hospitalisation, which was found to be associated with adverse clinical outcomes in the HOPE-SAM cohort clinical data analyses and with PC4 scores in our cross-sectional data. To clarify this, we have added "*at enrolment*" to all in text references to this adjustment.

Figure 5, again the biologic relevance of the PC axes is unclear to the reviewer, could the authors expand on how they derived PC2 to be 'pro-inflammatory' and PC1 'bacterial binding' scores
Figure 1B provides a full overview of the individual variables reflected in each composite variable (PC). We interpret PC2 as reflecting pro-inflammatory cytokine responses because variance in LPS-specific IL-6, TNFa and IL-8 and HKST-specific IL-6 and TNFa are most strongly loaded onto this PC (factor loadings >= +0.3) and PC1 as associated with bacterial binding because variance in proportions of E.coli+ innate immune cells and mean fluorescence intensity for E.coli binding are most strongly loaded onto this PC.

We have added text to further explain the PCA approach and scoring in the main text in response to Reviewer#1, which we hope also addresses your helpful comment.

Minor comments:
How did the authors deal with repeat measurements in their assessments of significance
We have used a mixed linear regression model with participant included as a fixed effect to account for repeated measures. We believe that we have reported this approach sufficiently in the manuscript.

The abstract is unclearly written for someone who does not go on to read the rest of the text
- It is unclear how the longitudinal cohort is related to cross-sectional cohort,
- It is unclear at which time point the results "upregulation… are dysregulated" refers
- referring to.
- It is unclear what 'inpatient immune function' and 'hospitalisation' refers to as relates to the two cohorts
We appreciate this comment and have now extensively rephrased the abstract to reflect your suggestions.

Line 43, anti-bacterial innate immune function may contribute; suggest to use 'may associate' as directionality of association not shown by preceding data
We have now rephrased this line.

Table 1: Nutritional status, Baseline MUAC missing parentheses before 11.2
Thank you for spotting this. We have now added the missing parentheses.

Supplementary figure 5 should have an F in legend
Thank you for spotting this. We have now corrected this typo for panel F in the figure legend.

Figure 4, Supplementary figure 6/7/8 font is very small/ineligible on figures, suggest to increase font size
We apologise for the small text size in the in-line version of our manuscript; we have now split Figure 4 into 2 separate figures for monocyte surface markers (new Figure 4) and cytokine responses (new Figure 5) for legibility. We have updated the Figure numbering in the main text accordingly.

Line 208, is the reference to Supplementary Figure 8 correct?
Thank you for spotting this. We have now corrected this typo which should have referred to Supplementary Figure 6.

Reviewer #3 (Remarks to the Author):

This manuscript addresses an important and understudied area - the relationship between immune recovery and nutritional rehabilitation among children hospitalized with complicated severe acute malnutrition (SAM). The authors hypothesize that immune restoration lags behind nutritional recovery and explore whether there is an association between the rehabilitation of innate immune function and anthropometric recovery up to 48 weeks post-discharge. The longitudinal design, incorporation of immune phenotyping, and long-term follow-up make this work relevant to understanding post-SAM vulnerability. However, several issues require clarification and reorganisation to strengthen the manuscript and improve interpretability.

Thank you for identifying our work as addressing important and understudied hypotheses. We outline how we have addressed the issues that you have raised point-by-point below.

Major Comments
1. Study Design and Clarity of Cohort Flow
• The description of the study design needs clearer articulation. It appears to be a prospective cohort study with hospitalised SAM children as the exposed group and community children as the comparison (unexposed) group. However, in several places, the text refers to ‘cases’ and ‘controls’, which can be confusing as this terminology implies a case–control design.

We agree that we could have been clearer in the main text; we have now re-written the first paragraph of the results section to clarify on our study design as it relates to the parent observational cohort study (HOPE-SAM). For this study and other sub-studies within the HOPE-SAM parent cohort, a group of controls were recruited for primary comparisons between children with SAM and adequately nourished non-hospitalised children. We use case/control terminology to align with our published study protocol for HOPE-SAM (Bwakura-Dangarembizi et al, BMJ Open, 2019). We hope that these edits address your concerns.

• The manuscript would benefit from a clear schematic (or an improved version of Figure 1A) showing participant flow, inclusion/exclusion criteria, follow-up visits, and numbers at each stage.

Thank you for this comment. To clarify on participant flow and more fully reflect inclusion/exclusion criteria in Figure 1A, we have added details of the 36 children in the cross-sectional IMMUNO-SAM cohort who were excluded because they provided blood samples <2mL during follow-up and therefore were not eligible for the longitudinal cohort. Children included in the longitudinal cohort (n=83) who missed a blood sample at follow-up visit/s were not excluded from analyses if they had available data at other follow-up visits, therefore numbers reflect how many participants had available immune function data for analysis at each visit; we have added this detail to the figure legend as follows:

"All IMMUNO-SAM cases and controls with complete immune function assessment were included in this study as the cross-sectional cohort (cases n=125, controls n=73) and all cases in the cross-sectional cohort who had complete immune function data at ≥1 follow-up visit were included in the longitudinal cohort (n=83); number and percentage of cases from the longitudinal cohort with available immune function data are indicated for each follow-up visit."

The current flow diagram contains inconsistencies in the numbers, which should be cross-checked and corrected.

Thank you for spotting this. We have checked Figure 1A and corrected the typo indicating that 15 cases had incomplete immune function data; this should have been 16 cases.

• Please clarify whether the control group comprised hospitalised or community children. If hospitalised, specify the indication for admission and confirm that they were free of acute infection, as this affects comparability.

Controls were not hospitalised. We have added '*non-hospitalised*' to the description of controls in the results and methods sections to clarify this.

2. Presentation of Baseline and Follow-up Data
• Table 1 should typically present baseline characteristics of the study population at enrolment. The current version includes follow-up information, which could be moved to a separate table or supplementary material.

We are happy to present the tables in a more typical format for the field and have now split the table into 2 tables as requested and added reference to the relevant table in the text.

• The authors should ensure that comparisons in the text (e.g., Lines 105–106) specify the reference group-whether comparisons are being made between SAM children with vs. without adverse outcomes, or between SAM children and healthy controls.

An important comment; we have now been through the manuscript to double check that we are specifying the reference group at each juncture and updated the text where this was not initially clear.

3. Causal interpretation and phrasing
• The manuscript occasionally implies causality (e.g., "immune restoration lags behind nutritional recovery" or "deficient innate immunity may underlie persistent morbidity") although this is an observational study with limited capacity for causal inference. The text should more consistently use cautious, associative language (e.g., "was associated with" rather than "led to").
• In the Abstract and Discussion, consider clarifying that the data are consistent with, but do not establish, a causal relationship between immune recovery and nutritional rehabilitation.

We appreciate these comments, recognise that this is an observational study and do not wish to overinterpret out data. We have now gone through the manuscript to replace instances where we have used 'led to' (or similar) with more cautious language ("may have led to", "associated with" or similar) throughout.

We stand by our use of 'lags behind', which is based on observations within the time window (i.e. hospital admission to 48 weeks post-discharge) where we have available data (changes in immune function and nutritional status) for this relative comparison. However, we have added the following statement to clarify our use of 'immune restoration' and 'normalisation' in our interpretation:

"For our longitudinal mixed effects linear regression of immune function variables by visit and case/control status, we refer to 'immune restoration' where cases had distinct scores for an immune function variable compared to controls at baseline, but these scores subsequently converged to be within control ranges during post-discharge follow-up. 'Normalisation' was indicated by scores becoming more like those of the control group, even they it remained significantly different at post-discharge visits."

4. Integration of Previously Published Data
• Sections of the Results (e.g., Lines 130–131) intermingle findings from the current analysis with results already reported from the HOPE-SAM study. These should be clearly separated: previously published findings should be referenced succinctly in the Introduction or Discussion to provide context, while the Results should focus only on new analyses presented here.

We have now more clearly separated reference to our previously published findings throughout the manuscript, particularly in the first section of the results where there is the most overlap. We have retained some information from our published work in the relevant sections of the results as Reviewer# 2 highlighted that more information was

needed to show how the clinical outcomes for our cohort relate to those of the parent cohort. However, we hope that this distinction is now clearer than the original version of the manuscript.

5. Adjustment for multiple testing
• Numerous regression models are fitted (e.g., Supplementary Tables 8–15). Please clarify whether any correction for multiple comparisons was applied. If not, discuss the potential for type I error inflation, particularly in exploratory analyses.

We have not adjusted p-values for multiple comparisons and instead present model coefficients alongside raw p-values. This approach preserves sensitivity while using a pre-specified significance threshold of $p<0.05$. Strict control of the family-wise error rate was not applied because our primary inference is focused on a small set of key hypotheses and composite variables (principal components) derived from factor reduction (PCA), rather than on the full set of exploratory tests. Analyses were planned around the minimal number of tests required to address our hypotheses, reducing the potential for type I error.

To clarify on this approach and address your comment directly in the text, we have added the following statements to the statistical methods section:

"Statistical analyses were planned to address our 3 key hypotheses with primary inference based on coefficients and p-values from adjusted models for PCs. Statistical significance is reported at uncorrected p-values <0.05."

6. Organisation of the Results Section
• Lines 153–160 include material that belongs in the Methods section, describing analytical approaches rather than findings.

Thank you for highlighting this. We have now removed this methodological detail from the Results section.

• Similarly, Lines 175–177 include interpretative statements that would be more appropriately placed in the Discussion. The Results should remain descriptive and focused on presenting data without interpretation.

We have rephrased this statement and others to ensure that we are providing summary statements to cover the collective result/s presented per each section of the Results rather than anticipating interpretation presented in the Discussion. Our edits reflect comments from yourself and the other Reviewers.

7. Quantification of Measures
• Clarify (Line 188) whether the measure being described is quantitative, categorical, or derived from a composite index. If it is a derived measure (e.g., immune restoration score), specify how it was calculated and validated.

Apologies for any confusion here, we are referring to concentrations of mediators present in unstimulated cultures and have clarified this here and in the title of the associated Supplementary Figure 9.

8. Discussion
• The Discussion would benefit from a clear opening paragraph summarising the principal findings of this study before interpreting them in the context of prior evidence. Currently, context and background at the start of the Discussion slow the presentation of key results—reverse this order for improved readability.

We have now re-ordered this paragraph accordingly.

• Interpretation appears interspersed throughout; restructuring it to flow from summary → interpretation → implications → limitations → conclusions would improve readability.

Thank you for these suggestions; we hope that our combined responses to your comments and those of Reviewers#1 and 2 have significantly improved the structure and readability of the manuscript in response to your comments whilst retaining pertinent context where needed/requested.

9. Selection bias and representativeness
• As acknowledged (lines 407–411), children able to provide ≥2 mL of blood likely represent a healthier subset of the SAM population. The discussion could elaborate further on how this might bias findings (e.g., underestimating the magnitude of immune dysfunction or the association with adverse outcomes).

Thank you for this suggestion, we have expanded on our limitations section of the discussion to include the following statement:

"Furthermore, the longitudinal changes we observe in immune function may underestimate those seen in more extreme cases of complicated SAM."

10. Clinical translation
• The conclusion suggests that delayed immune recovery may warrant adjunctive interventions to prevent infection. This is a strong and important implication but would benefit from referencing potential candidate interventions (e.g., anti-inflammatory or microbiota-directed approaches) or current evidence gaps. Otherwise, the recommendation seems somewhat speculative.

Thank you for this comment which raises an important topic for future directions in this feel. We do not believe that there is sufficient evidence currently to inform the 'right' intervention to take based on our data or the wider literature (where we believe assessment of immune function is sorely lacking) so would prefer to keep this as an open/speculative statement. To address your comment and this knowledge gap more explicitly, we have added the following statement to the conclusion:

"Further studies are needed to identify whether interventions targeting innate immune cell function could contribute to a fuller recovery of children after hospitalisation with complicated SAM."

11. Timing of early follow-up
• The 12-week gap between discharge and the next immune assessment is a major limitation given that most adverse events occur in this window. It would be helpful to explicitly note this as the period where immunological and clinical trajectories may diverge most, reinforcing the need for finer-grained sampling in future studies.

We agree that it would have been desirable to have finer granularity on immune function outcomes however the timings of blood samples were pre-specified according to requirements by our study ethics committee. We acknowledge this as a limitation of our dataset in the discussion as follows:

"For example, pre-specified blood sampling regimens meant that we did not evaluate immune function between the discharge and 12 week visit, during which the majority of adverse clinical outcomes occurred during HOPE-SAM follow-up[2, 3]."

12. Statistical power and missingness
• The low event rates (e.g., 14 deaths, 22 readmissions) limit the ability to detect associations between immune parameters and clinical outcomes. The authors might quantify power (post hoc) or provide confidence intervals for null findings to emphasize interpretive caution.

Whilst we agree that we are reporting on a low event rate, we respectfully disagree that post-hoc power calculations would be appropriate here as they can themselves be misleading. To reflect your comment and to be transparent about our data throughout, we report our sample sizes in all Tables, text and Figure legends and explicitly highlight the low event rate as a limitation in our Discussion as follows:

"As such, incidence of adverse clinical outcomes was low; evaluation of their associations with immune cell function have low statistical power and should be considered exploratory."

Minor Comments

1. Terminology
   • Use consistent terminology for 'complicated SAM' vs. 'hospitalised with SAM' throughout. Occasionally both terms are interchanged without definition'.

We have now ensured that we specify '*children hospitalised with complicated SAM'* throughout the manuscript so that these terms do not appear interchangeable.

• Replace 'nutritionally cured' (p.4) with 'met discharge nutritional criteria' or 'achieved nutritional recovery' to avoid ambiguity.

Response to Reviewer comments

We agree that this term is broad, however we have opted to retain the terminology as is in quotation marks as this was the term used by the authors of the systematic review we are reporting here which summarises studies with heterogenous definitions of nutritional 'cure'.

2. Methods

• Clarify the timing of the 'baseline' sample (admission day vs. during hospitalization). It seems to vary (lines 520–522).

We have added the following statement to clarify this:

"For cases, it was not always possible to collect blood samples on the day of hospital admission due to critical illness, therefore baseline immune function assessment was conducted on the first blood sample ≥2mL collected from a participant which could be at any time during their hospital stay, as previously reported[21]."

• Provide justification for using PCA-derived components rather than mixed models on individual immune variables—particularly since interpretation of PCs over time may shift.

We have provided fuller details of the PCA approach to the second section of the Results, including a statement which specifically addresses your comment as follows:

"These composite variables have the advantage of reducing the number of variables for statistical analyses and better reflect the interacting nature of anti-bacterial immune responses in vivo than evaluating individual immune functions one at a time."

3. Figures

• Figures 3–6 are data-rich but crowded. Adding inset legends explaining what positive/negative changes mean biologically (e.g., ↑PC1 = higher bacterial binding) would improve accessibility for non-specialist readers.

We have now added the following statement to the Results section to clarify on interpretation of PC scores for Figures 3-6 (now Figures 3-7 in the revised version):

"PC scores correspond to patterns of the immune function variables with which they are loaded (e.g. PC2 is positively loaded with tumour necrosis factor (TNF)α, interleukin (IL)-6 and IL-8) in response to bacterial antigens (LPS and HKST), therefore higher scores for PC2 reflect higher pro-inflammatory cytokine responses to bacterial antigens)."

• Figure 2D could include correlation coefficients or trend lines to visualize the strength of associations with ΔMUAC.

We fully agree and had included trend lines and coefficients in our original version of Figure 2D; we apologise if these were not visible in the version that you received and have re-exported the figure for the revised version of the manuscript.

4. Discussion

• The discussion effectively integrates murine evidence but could more clearly distinguish which mechanisms are speculative versus supported by human data.

We have now added statements in the discussion to clarify where mechanisms presented in murine data or human cohorts affected by other conditions (e.g. sepsis) remain to be determined in children with SAM.

• The statement that "bacterial binding capacity remained abnormal in recovered children" (p.17, line 347) could be tempered by acknowledging that the differences, while statistically significant, may or may not be clinically meaningful.

We have added a more thorough overview of what our findings regarding change in bacterial binding capacity may/may not mean clinically in the Discussion section; as suggested by Reviewer#1 (above).

5. Others

• Line 80: Clarify the characteristics of the control group — were they hospitalised, or community-based?

We have clarified on this throughout the text, as outlined in response to your earlier comment.

• Line 84: Replace 'cases and controls' with 'SAM and comparison groups' or equivalent terminology consistent with a cohort study design.

We have retained the case/control terminology as justified in response to your comment above. We believe this to reflect the study design and to be clearer than SAM/controls since we compare children with SAM (the case group) both cross-sectionally (e.g. with versus without adverse event) and longitudinally (e.g. follow-up visits versus baseline). We hope that the revised version of the manuscript sufficiently addresses your concern.

• Line 146: Rephrase to avoid switching between 'our cohort' and 'cases' in the same sentence. Suggestion: "Our cohort data indicated that hospitalised children with SAM..."

We have rephrased this sentence accordingly.

• Line 353: Typographical error — replace where with were.

Thank you for spotting this. We have now corrected relevant instances in the text.

• Figure 1A: Verify participant numbers for consistency throughout.

Thank you for this comment, we have now done this and responded to your earlier comment on this above.

Response to Reviewer comments

• Please include the statistical comparison methods used for between-group analyses in the Methods (e.g., regression models, mixed models, or non-parametric tests).

We have now rephrased our statistical methods section to clarify the dependent and independent variables for each regression model.

Overall Assessment

This is a valuable and well-conceived study that makes a meaningful contribution to understanding the immunological recovery trajectory following SAM. The analysis is novel, but the manuscript would benefit substantially from clearer description of the design, improved data presentation, and stricter separation of Methods, Results, and Discussion. Addressing these points will improve study clarity, strengthen the reporting, and support the impact and reproducibility of your findings.

Thank you for this assessment of the value of our work. We agree that your suggestions have improved the clarity of our manuscript.

RESPONSES TO REVIEWER COMMENTS

Thank you to Reviewers 1, 4 and 5 for their time and consideration of our resubmitted manuscript. Your comments are reproduced in full below (black text) and our responses to each are provided below the relevant comment (red text). We indicate where a comment has been addressed or our response to your comment has been modified in response to another Reviewer.

Reviewer #1 (Remarks to the Author):

Comments were addressed adequately.

Thank you for confirming that we have addressed your comments. We believe that out manuscript is much improved by your input.

Minor revision:
Figure 3 panels B and C remain too small to read, even with significant magnification.

Thank you for flagging this. We have reconfigured the layout of Figure 3 to expand the size of panels B and C for legibility and are now submitting all Figures as vector files to preserve legibility for the final article.

Reviewer #4 (Remarks to the Author):

I have been asked to review this manuscript following a round of revisions as one of the original reviewers was unable to follow-up their review.

This manuscript describes work that is novel, important, and methodologically sound. The noteworthy results and importance to the field have been outlined by the prior reviewers, and I concur. The manuscript is well written, the interpretation and conclusions are appropriately phrased and are in line with the data presented. The manuscript has been improved by the round of revisions, and I consider that all the prior reviewers' comments have been appropriately addressed. There are no flaws in the data analysis or other aspects of the manuscript that would require a further round of revisions. Data and methodology are provided adequate to support reproduction.

I am in favour of the manuscript now moving forward to publication without further delay.

Thank you for reviewing our revised manuscript and responses to the original round of reviewers. We appreciate your support for our work and recommendation for publication without further review.

Reviewer #5 (Remarks to the Author):

I was asked to examine author responses to Reviewer 2 and find that the authors have satisfactorily addressed most of the issues raised by Reviewer 2 in an unbiased and transparent way.

Thank you for reviewing our revised manuscript and responses to the original round of reviewers.
